# Supplementary material for: Mutant Ras and inflammation-driven skin tumorigenesis is suppressed via a JNK-iASPP-AP1 axis
Source: Cell Rep. 2022 Oct 18;41(3):111503. doi: 10.1016/j.celrep.2022.111503 (PMC9597577; doi:10.1016/j.celrep.2022.111503)
Supplement: Document S1. Figures S1–S7 and Tables S1 and S2 [file mmc1.pdf]

Cell Reports, Volume *41*

Supplemental information

Mutant Ras and inflammation-driven skin tumorigenesis is suppressed via a JNK-iASPP-AP1 axis

Khatoun Al Moussawi, Kathryn Chung, Thomas M. Carroll, Christian Osterburg, Artem Smirnov, Rebecca Lotz, Paul Miller, Zinaida Dedeić, Shan Zhong, Martin Oti, Evelyn N. Kouwenhoven, Ruth Asher, Robert Goldin, Michael Tellier, Shona Murphy, Huiqing Zhou, Volker Dötsch, and Xin Lu

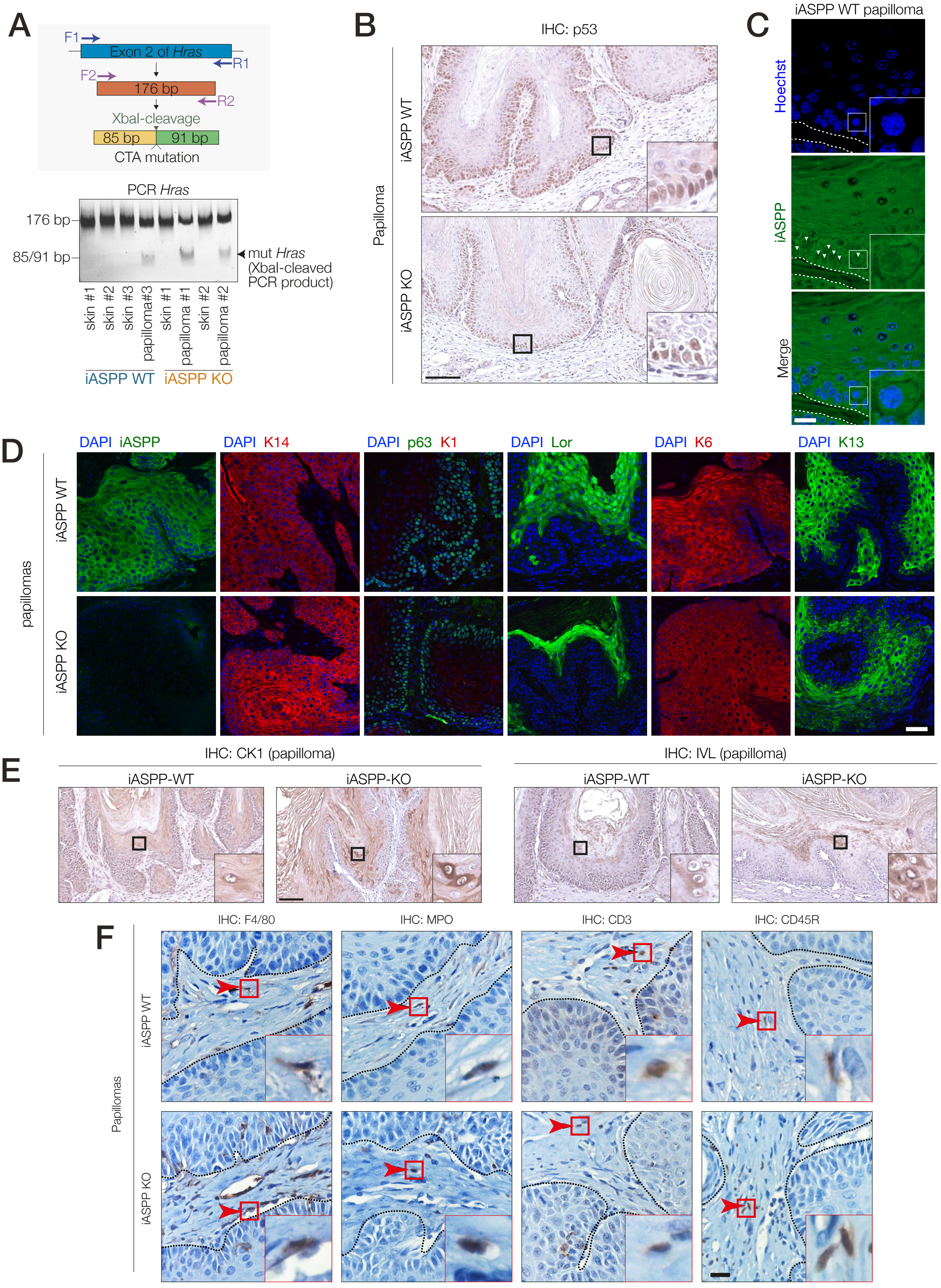

Figure S1. iASPP suppresses skin tumorigenesis driven by chemically-induced mutant Ras and inflammation. Related to Figure 1 **(A)** (Top) Scheme showing the location of primers for detection of Hras mutation. (Bottom) PCR detection of Hras mutation in skin and papilloma samples from WT and iASPP KO mice. **(B)** IHC analysis of p53 expression in WT and iASPP-KO papilloma samples. Scale bar 100 μm. **(C)** IF staining of iASPP expression in a WT papilloma sample. Scale bar 25 μm. **(D)** Immunofluorescence staining of iASPP, cytokeratin 14, p63, cytokeratin 1, loricrin, cytokeratin 6, and cytokeratin 13 in papillomas from WT and KO mice. Scale bar 25 μm. **(E)** IHC analysis of keratin 1 (CK1) and involucrin (IVL) expression in WT and iASPP-KO papilloma samples. Scale bar 100 μ m. **(F)** Immunohistochemical analysis of expression of F4/80 (macrophages), MPO (neutrophils), CD3 (T cells), and CD45R (B cells) in papillomas from WT and KO mice. Arrowheads indicate examples of stained immune cells. Dotted line shows boundary between epidermis and stroma. Scale bar 25 μm.

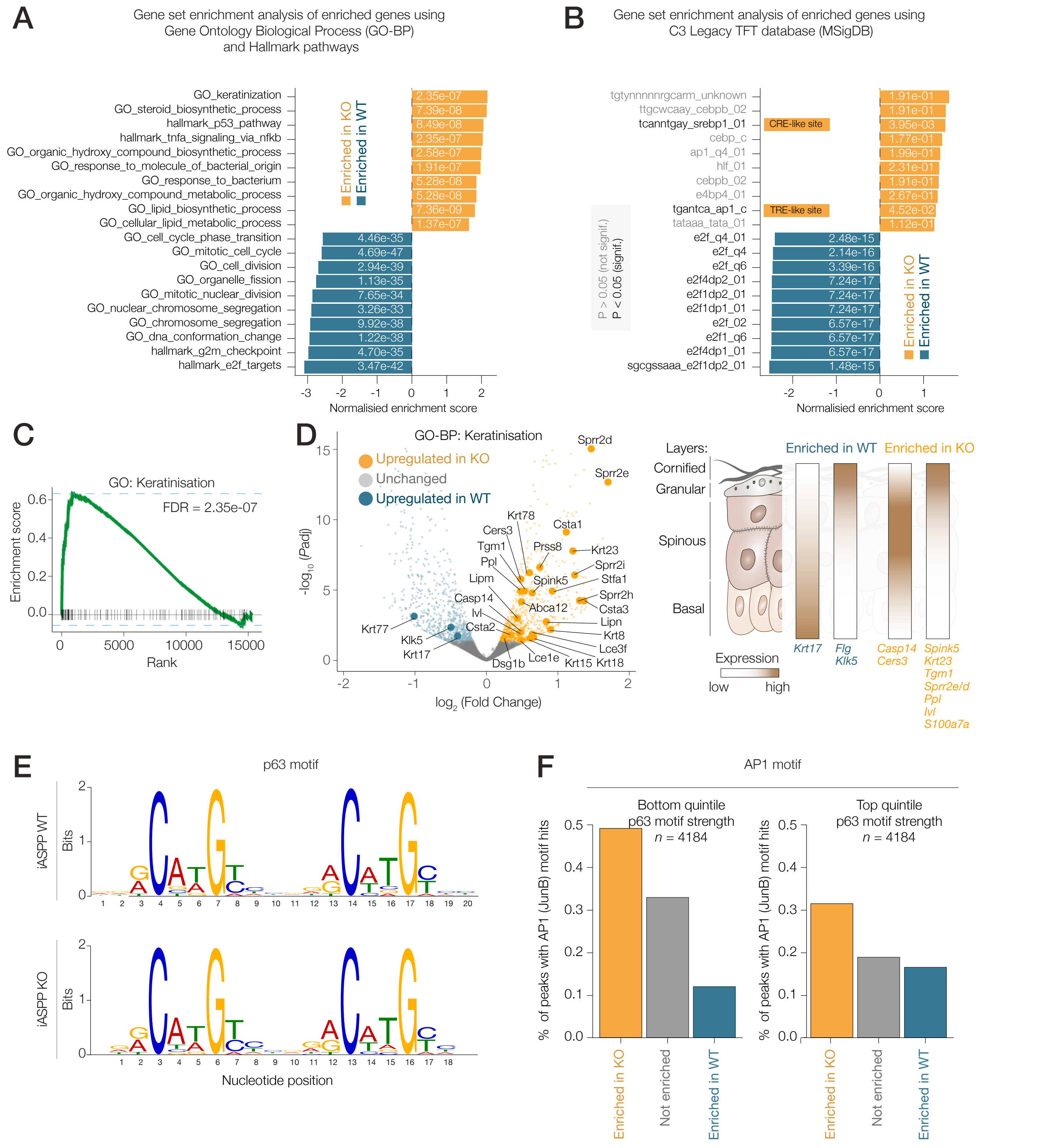

Figure S2. Loss of iASPP selectively enhances p63 binding to regions that often contain AP1 motifs. Related to Figure 2 **(A)** FGSEA pathway analysis using moderated fold changes to identify significantly enriched pathways within the Hallmark and Gene Ontology Biological Process pathway collections. The top 10 significant pathways in iASPP KO and WT were identified by the adjusted p-value, with the magnitude of the normalized enrichment score (NES) as tiebreaker if needed. For each pathway, the NES is represented by the length of the bar along the x-axis, and the adjusted p-value is displayed on top of the bar. **(B)** FGSEA pathway analysis as in A but using the C3 Legacy TFT database (MSigDB), which lists genes that have highly conserved instances of TF motifs in their promoter regions. Only two pathways reach significant enrichment in iASPP KO following p-value adjustment; these are in bold for emphasis. **(C)** FGSEA enrichment plot for GO-BP Keratinization pathway. **(D)** (Left) Genes from the GO Keratinization pathway with an adjusted p-value < 0.05 in differential expression analysis are highlighted on the RNA-seq plot. (Right) Schematic showing expression levels of modulated genes from GO keratinization pathway across the layers of human epidermis. **(E)** Consensus p63 motifs identified in iASPP WT-enriched and KO-enriched p63 peak regions (with MAnorm $p < 5e-3$), as determined by STREME analysis. **(F)** Peak sequences were scored for the presence or absence of the AP1 (JUNB) motif using an FDR threshold of 1e-3 with the representative MA0490.1/JUNB motif. The percentages of peaks containing this AP1 motif were calculated for the peak enrichment categories defined in (A), and the results are plotted in these barplots. The figure is faceted depending on the quintiles of the score assigned by FIMO to the top hit for a p63 motif in each sequence; on the left facet, the bottom quintile (weakest/absent p63 motifs) subset is shown, while the right facet shows the top quintile of peaks, with the highest similarity to the canonical p63 motif in JASPAR.

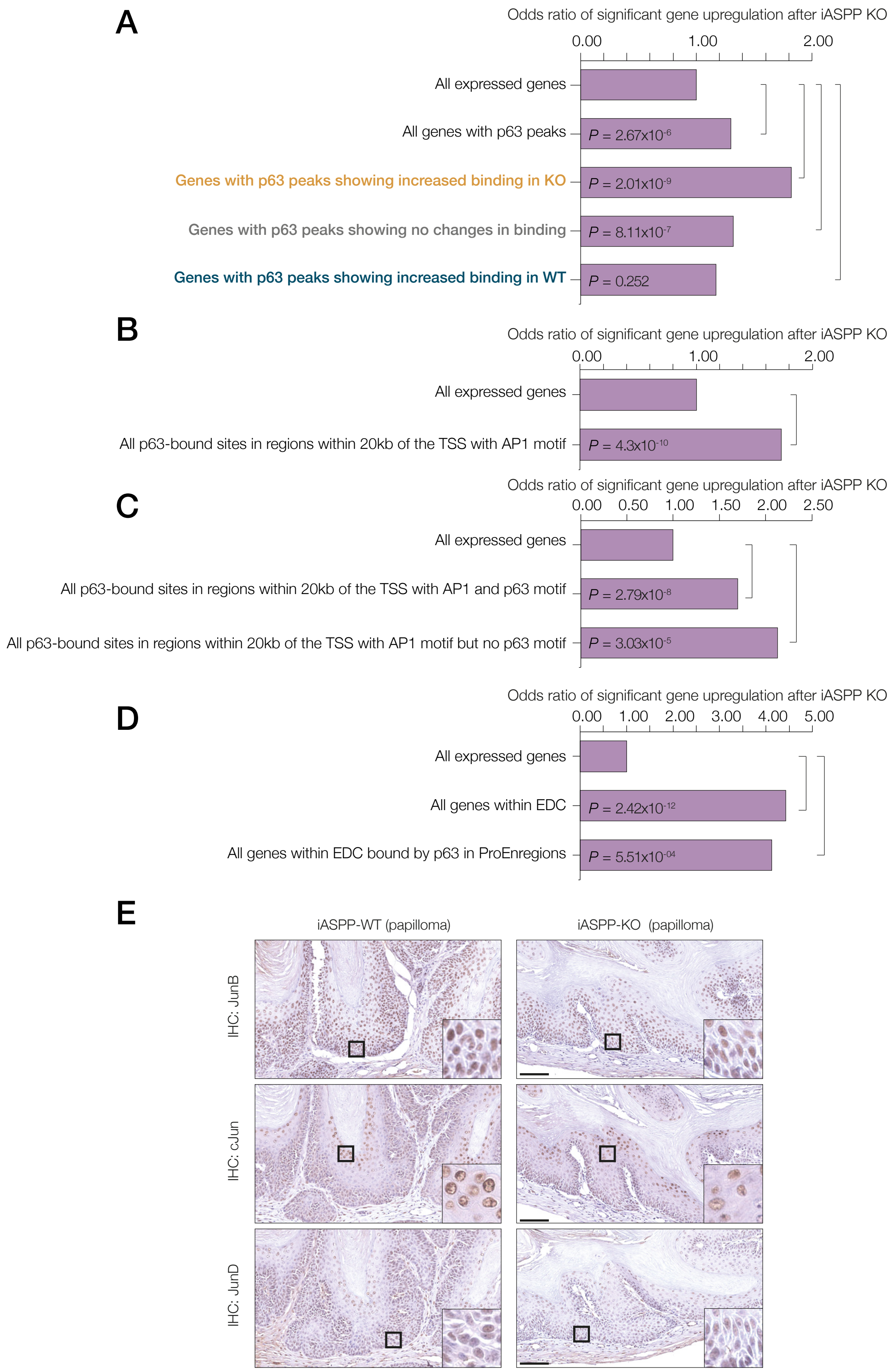

Figure S3. iASPP depletion affects expression of epidermal differentiation genes. Related to Figure 3 **(A)** Barplots show the odds of significant gene upregulation after iASPP KO based on membership in categories defined from ChIP-seq data. In each case, we investigate the defined subsets in relation to the full reference set of 15,320 expressed genes, of which 969 (6.3%) are upregulated. For each bar, we provide the odds ratio, defined as the proportion of upregulated genes in the subset divided by the same proportion in the reference set, and the associated value as calculated by a Pearson's chi-squared test. In the top subplot, subcategories are defined by the ChIP-seq enrichment class defined in 2B; in the bottom, the subcategories are defined by the presence or absence of AP1 or p63 motifs, with a FIMO FDR of 0.001 used as a threshold to call the presence of motifs in each peak region sequence. **(B)** Barplots as in A defined by the presence of AP1 motif. **(C)** Barplots as in A defined by the presence of AP1 motif and presence or absence of p63 motifs. **(D)** Odds ratios for significant gene upregulation and the associated p-value are shown for comparisons between all expressed genes in the EDC (n=68, 27.9% significantly upregulated) and the reference set of all expressed genes (n=15,320, 6.3% significantly upregulated). A comparison is also done with a subset of expressed EDC genes with a p63 binding site within 20kb of the TSS (n=23, 26.1% significantly upregulated). **(E)** IHC analysis of JunB, cJun, and JunD expression in WT and iASPP-KO papilloma samples. Scale bar 100 μm.

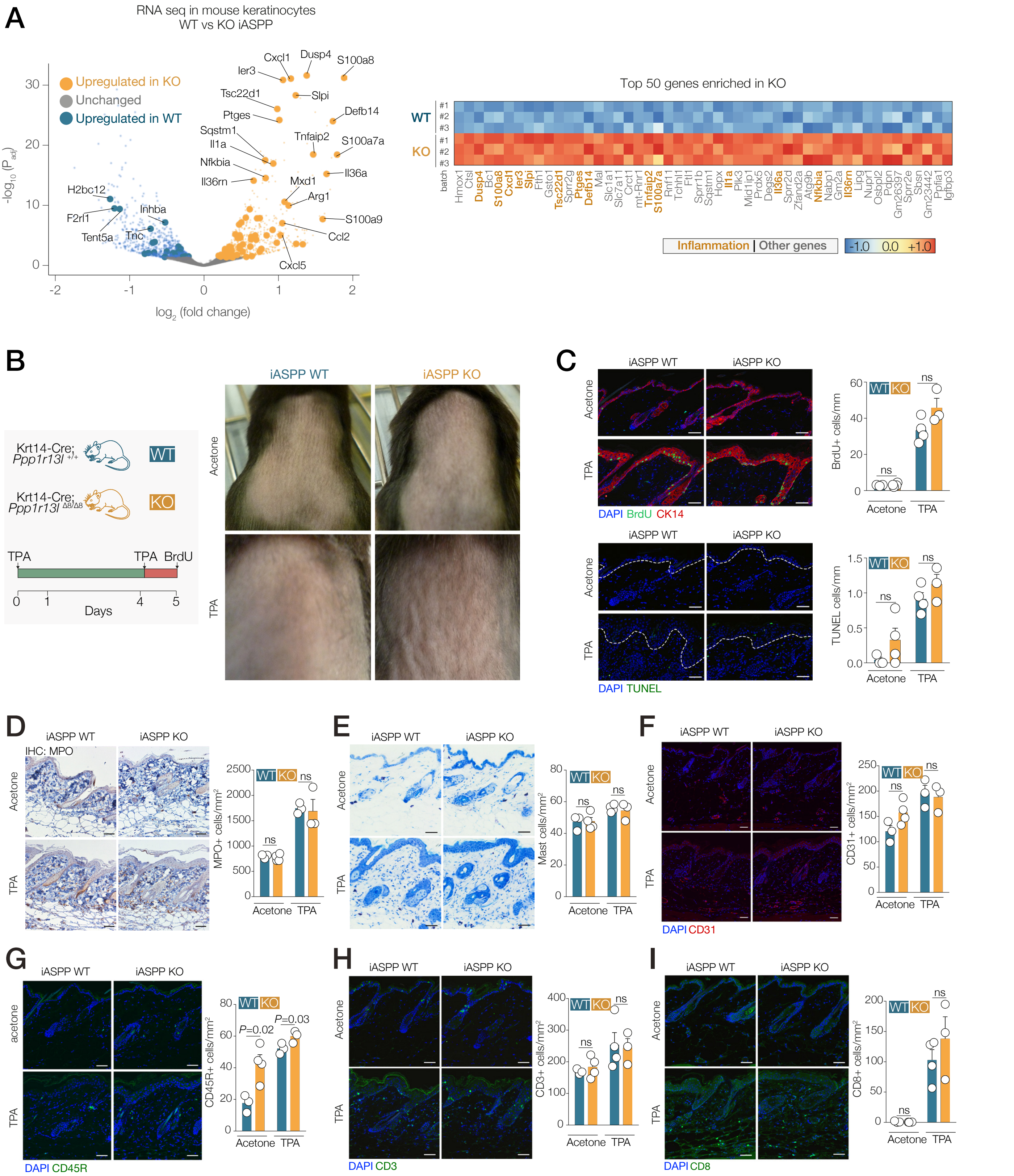

Figure S4. iASPP deficient keratinocytes induce pro-inflammatory gene expression in vitro and attract macrophages in vivo. Related to Figure 4 **(A)** Volcano plot shows the moderated log fold changes and associated adjusted p-value after iASPP knockout. Genes are assigned a colour depending on whether they are determined to be as significant (adjusted p-value < 0.05) and in which condition they are significantly enriched in. Significantly differentially expressed genes in two inflammatory pathways (Hallmark: TNFA_SIGNALING_VIA_NFKB and GO-BP: RESPONSE_TO_BACTERIUM) are labelled. On the right, heatmap showing the 50 genes most significantly enriched in iASPP KO. Genes belonging to these same two pathways are highlighted. **(B)** (Left) Schematic illustration of the TPA inflammation induction protocol. (Right) Representative images of treated skin in WT and iASPP KO mice. **(C)** Immunofluorescence analysis (left) and quantification (right) of BrdU-positive and TUNEL-positive cells in skin samples from WT and iASPP KO mice upon acetone or TPA treatment. Dashed line shows boundary between epidermis and dermis. n=3 (WT, acetone cohort), n=4 (KO, acetone cohort), n=4 (WT, TPA cohort), n=3 (KO, TPA cohort). **(D)** Immunohistochemical analysis (left) and quantification (right) of MPO-positive cells in skin samples from WT and iASPP KO mice upon acetone or TPA treatment. **(E)** Immunohistochemical analysis (left) and quantification (right) of mast cells in skin samples from WT and KO mice upon TPA treatment. **(F)** Immunofluorescence analysis (left) and quantification (right) of CD31-positive cells in skin samples from WT and KO mice upon TPA treatment. **(G)** Immunofluorescence analysis (left) and quantification (right) of CD45R-positive cells in skin samples from WT and KO mice upon TPA treatment. **(H)** Immunofluorescence analysis (left) and quantification (right) of CD3-positive cells in skin samples from WT and KO mice upon TPA treatment. **(I)** Immunofluorescence analysis (left) and quantification (right) of CD8-positive cells in skin samples from WT and KO mice upon TPA treatment. For C-I, *P* value was calculated by t-test with false discovery (BKY) correction for multiple testing. Scale bar 100 μm.

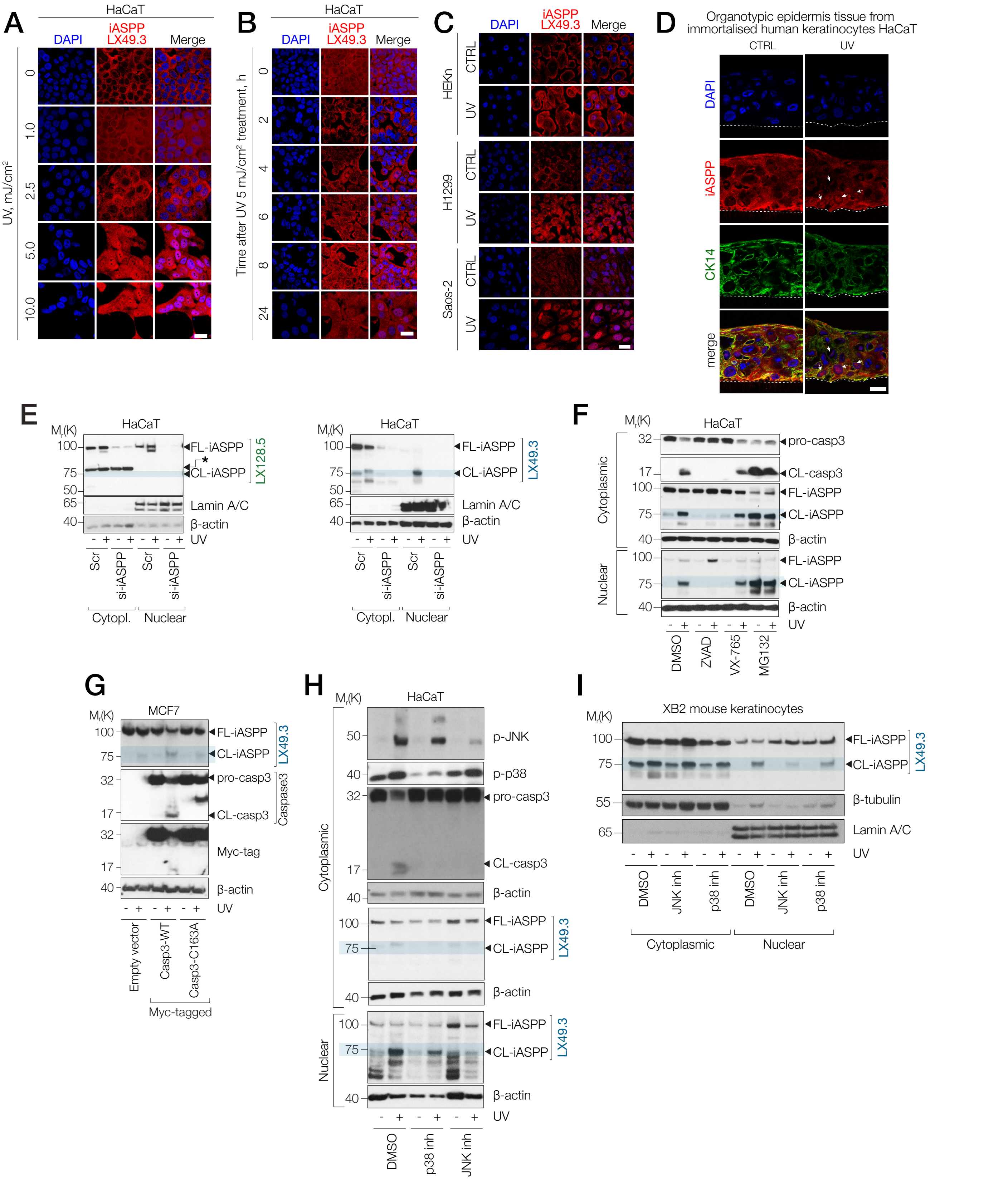

Figure S5. Cross regulation between iASPP and JNK/AP1. Related to Figure 5 **(A)** Immunofluorescence analysis of iASPP colocalisation in HaCaT cells irradiated with increasing doses of UV. Cells were subjected to immunostaining 24h after UV irradiation. Scale bar 10 µm. **(B)** Immunofluorescence analysis of iASPP localisation in HaCaT cells irradiated with 5 mJ/cm² UV. Cells were subjected to immunostaining at 2h, 4h, 6h, 8h, and 24h after UV irradiation. Scale bar 10 µm. **(C)** Immunofluorescence analysis of iASPP localisation in HEKn, H1299, and Saos-2 cells irradiated with 5 mJ/cm² UV. Cells were subjected to immunostaining 24h after UV irradiation. Scale bar 10 µm. **(D)** Immunofluorescence analysis of iASPP (LX49.3) and cytokeratin 14 (CK14) expression levels in organotypic epidermis tissue from HaCaT cells. Tissues were irradiated with 150 mJ/cm² UV. Sections were collected and subjected to immunofluorescence analysis 24h after UV irradiation. Scale bar 25 µm. **(E)** Western blot analysis of iASPP expression levels in cytoplasmic and nuclear fractions of HaCaT cells upon 5 mJ/cm² UV irradiation after iASPP knock-down. Lamin A/C was used as a marker of nuclear fraction. iASPP was detected by either LX49.3 or LX128.5 anti-iASPP antibodies. * refers to a non-specific band recognized by LX128.5 at around 78 kDa in the cytoplasmic fraction in the left panel of S5E. **(F)** Western blot analysis of iASPP and caspase-3 expression in cytoplasmic and nuclear fractions of HaCaT cells pre-treated with either pan-caspase inhibitor (ZVAD), caspase-1/4 inhibitor (VX-765) or proteasome inhibitor (MG132) for 1h and exposed to 5 mJ/cm² UV irradiation. Cells were collected 24h after UV irradiation. **(G)** Western blot analysis of iASPP, caspase-3, and myc-tag expression levels in MCF7 cells transfected with either wild-type (WT) or mutant (C163A) myc-tagged caspase-3 for 24h and then exposed to 5 mJ/cm² UV irradiation. Cells were collected 24h after UV irradiation. **(H)** Western blot analysis of iASPP, p-JNK, p-38, and caspase 3 expression levels in the samples from Figure 5H after subcellular fractionation. **(I)** Western blot analysis of iASPP expression levels in the same treatments after subcellular fractionation of XB2 mouse keratinocytes. β-tubulin and lamin A/C as fractionation controls.

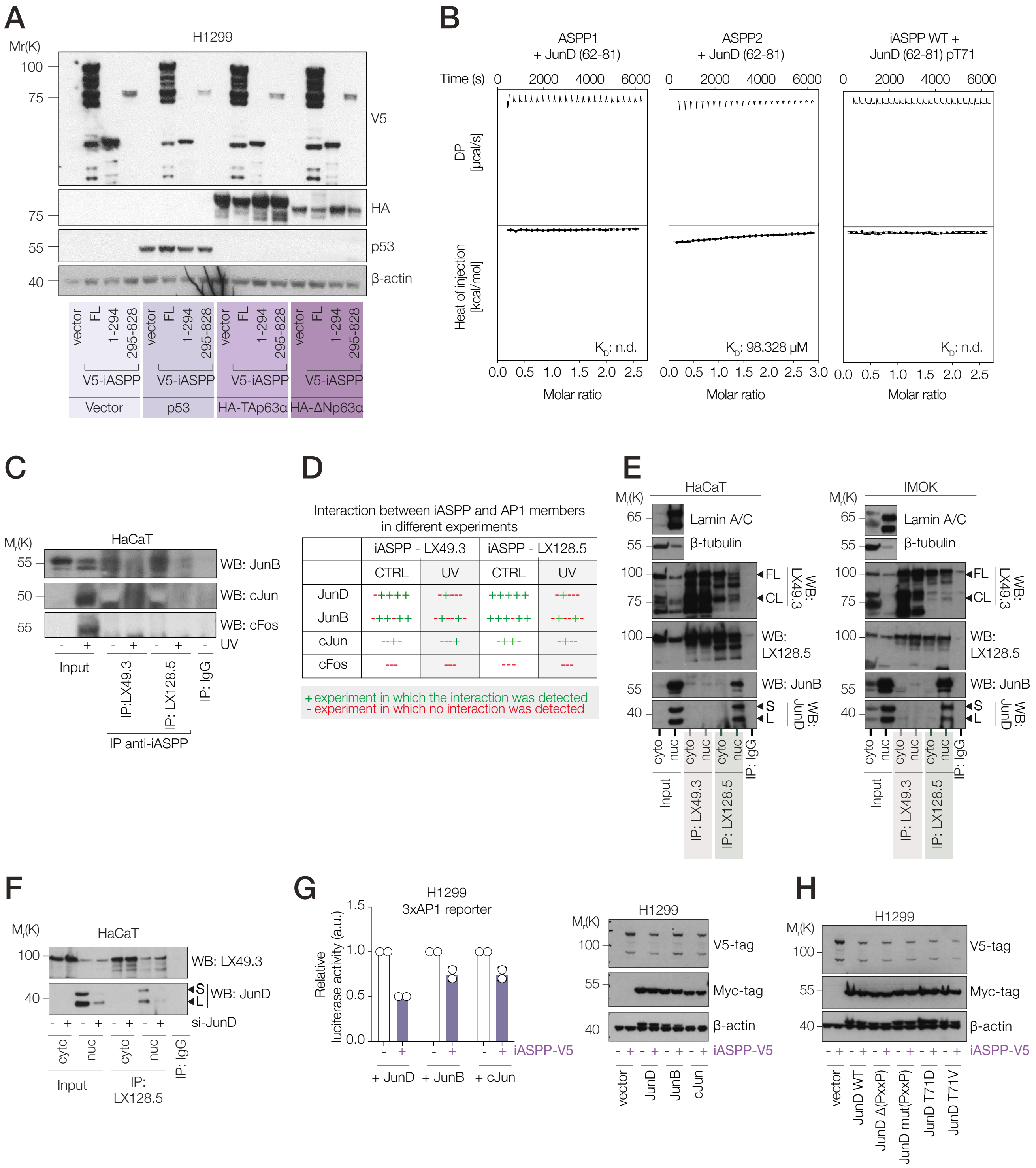

Figure S6. iASPP SH3 domain interacts with JunD N-terminal PxxP motif to inhibit AP1 transcriptional activity. Related to Figure 6 **(A)** Western blot analysis of expression of p63, p53 and iASPP mutants in samples from Figure 6A. **(B)** ITC results of JunD peptide binding to either ASPP1 (left) or ASPP2 CTD (middle) and JunD pT71 peptide binding to iASPP CTD (right). Raw titration profiles are displayed in the top diagrams and integrated heat in the bottom diagram. Best fit of single-site binding model is shown as a solid black line with the resulting equilibrium binding constant (KD). All other fit parameters are provided in Table S2. **(C)** Co-immunoprecipitation using anti-iASPP antibodies in lysates from Figure 6F. Western blot for JunB, c-Jun, and c-Fos. **(D)** Table showing whether AP1 members were co-immunoprecipitated with iASPP using LX49.3 or LX128.5 anti-iASPP antibodies in different experiments (biological replicates). "+" (green) indicates that interaction was detected, while "-" (red) indicates that no interaction was detected. **(E)** Co-immunoprecipitation using anti-iASPP antibodies in lysates of HaCaT or IMOK cells subjected to subcellular fractionation. Western blot for JunB and JunD. β-tubulin and lamin A/C as fractionation controls. **(F)** Co-immunoprecipitation using anti-iASPP antibody LX128.5 in lysates of HaCaT cells subjected to subcellular fractionation after JunD knock-down (si-JunD). Western blot for JunD and iASPP. **(G)** (Left) Luciferase activity assay in H1299 cells using 3xAP1 reporter after overexpression of V5-tagged iASPP combined with Myc-tagged JunD, JunB or c-Jun. Luciferase luminescence was normalised over renilla luminescence. Values shown are mean fold change over empty vector ± SD, n = 2 (biological replicates). (Right) Western blot confirming the overexpression of V5-tagged and Myc-tagged proteins. **(H)** Western blot confirming the overexpression of V5-tagged and Myc-tagged proteins in the samples from the Figure 6H.

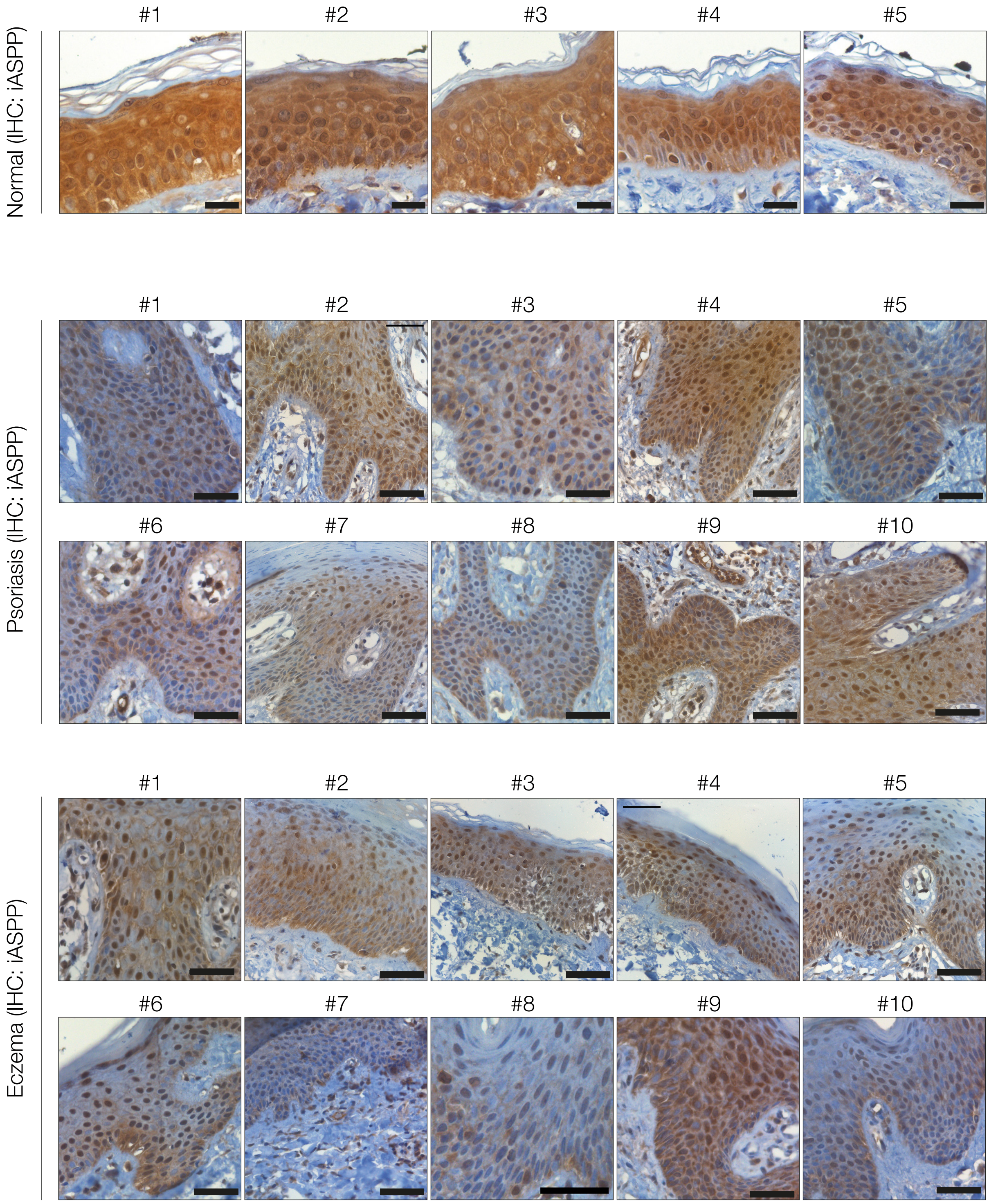

Figure S7. iASPP expression in human skin samples. Related to Figure 5. IHC analysis of iASPP expression on samples of normal human skin or skin from patients affected by psoriasis or eczema. Scale bar 100 µm.

Table S1. List of the primers used in the study, Related to "*Mouse Experiments and Genotyping*", "*mRNA extraction and qPCR*" and "*Cloning and mutagenesis*" sections of the STAR Methods

**qPCR primers**	
Name	Sequence
Ppp1r13l - For	AGACTCCAGCACCTCAACAGA
Ppp1r13l - Rev	GACCGTGCCTTCATCTGC
S100a8 - For	CCTTTGTCAGCTCCGTCTTC
S100a8 - Rev	TAGAGGGCATGGTGATTTCC
S100a9 - For	GCTGCATGAGAACAACCCAC
S100a9 - Rev	TCCCTTTAGACTTGGTTGGGC
IL1a - For	ATCAGCAACGTCAAGCAACG
IL1a - Rev	AGGTGCTGATCTGGGTTGGA
Il36a - For	CCCACTCATTCTGACCCAAG
Il36a - Rev	GAGAGAGTGCCACAGAGCAA
Il36b - For	TGCCTGCTGTCATAACTTCG
Il36b - Rev	CAAGATTGTGGAGGGAAAGC
IL6 - For	AGAAGGAGTGGCTAAGGACCA
IL6 - Rev	AACGCACTAGGTTTGCCGA
IL1b - For	CATCTCGGAGCCTGTAGTGC
IL1b - Rev	CGTGGACCTTCCAGGATGAG
TNF - For	GGCAGGTCTACTTTGGAGTCATTGC
TNF - Rev	ACATTCGAGGCTCCAGTGAATTCGG
CXCL1 - For	CCAGAGCTTGAAGGTGTTGC
CXCL1 - Rev	TCTCCGTTACTTGGGGACAC
GAPDH - For	CAGCAAGGACACTGAGCAAGA
GAPDH - Rev	GGCCCCTCCTGTTATTATGG
**PCR primers for mice genotyping**	
Name	Sequence
iASPP exon8 - FLP2	CCGAATTGGAGAAGTGAAGC
iASPP exon8 - I8-2	CCGAATTGGAGAAGTGAAGC
iASPP exon8 - E8-2	AGAGCAGCCTCAGAGCATGG
loxP - FLP2	CCGAATTGGAGAAGTGAAGC
loxP - FRANT9	GGGTAGGAAAAAGGGCTGAG
K14-Cre - R	ATTCTCCCACCGTCAGTACG
K14-Cre - 1	GCTCTCTGTCACCCTGGCTA
Actin - For	GGTGTCATGGTAGGTATGGGT
Actin - Rev	CGCACAATCTCACGTTCAG
Cre+ERT - F	CATTTGGGCCAGCTAAACAT
Cre+ERT - B	ATTCTCCCACCGTCAGTACG

Table S1. List of the primers used in the study (continued)

**PCR primers for detection of mutant Ras**	
Name	Sequence
Ras-Mut-1 - For	CTGTGAATTCTCTGGTCTGAGGAG
Ras-Mut-1 - Rev	TAGGTGGCTCACCTGTACTG
Ras-Mut-2 - For	CTAAGCCTGTTGTTTTGCAGGAC
Ras-Mut-2 - Rev	GGAACTTGGTGTTGTTGATGGC
**PCR primers for cloning**	
Name	Sequence
ΔNp63 AA2 BamHI - For	TACTTCCAGGGATCCTTGTACCTGGAAAACAATGCCCAG
p63α Stop XhoI - Rev	TACTTCCAGCTCGAGTCACTCCCCCTCCTCTTTGATGC
c-Fos AA3 BamHI - For	TACTTCCAGGGATCCTTCTCGGGCTTCAACGCAGAC
c-Fos Stop XhoI - Rev	TACTTCCAGCTCGAGTCACAGGGCCAGCAGCGTGG
c-Jun AA2 BamHI - For	TACTTCCAGGGATCCACTGCAAAGATGGAAACGACCTTC
c-Jun Stop XhoI - Rev	TACTTCCAGCTCGAGTCAAAATGTTTGCAACTGCTGCGTTAGC
JunB AA2 BamHI - For	TACTTCCAGGGATCCTGCACTAAAATGGAACAGCCCTTC
JunB Stop XhoI - Rev	TACTTCCAGCTCGAGTCAGAAGGCGTGTCCCTTGACC
JunD AA2 BamHI - For	TACTTCCAGGGATCCGAAACACCCTTCTACGGCGATG
JunD Stop XbaI - Rev	TACTTCCAGTCTAGATCAGTACGCGGGCACCTGGTG
JunD ΔPxxP - For	CTGCGGCCGCGCCGGCCGACGGCGCCCC
JunD ΔPxxP - Rev	GGGGCGCCGTCGGCCGGCGCGGCCGCAG
JunD mutPxxP - For	CTGCGGCCGCGCCGGCCCCTACCGCCCTGGCCGCCGACGGCGCCCC
JunD mutPxxP - Rev	GGGGCGCCGTCGGCGGCCAGGGCGGTAGGGGCCGGCGCGGCCGCAG
JunD T71D - For	GGCCGCGCCGCCTCCTGACCCCCTGCGCGCCGACG
JunD T71D - Rev	CGTCGGCGCGCAGGGGGTCAGGAGGCGGCGCGGCC
JunD T71V - For	GGCCGCGCCGCCTCCTGTGCCCCTGCGCGCCGACG
JunD T71V - Rev	CGTCGGCGCGCAGGGGCACAGGAGGCGGCGCGGCC
HisX Halo NcoI - For	TACTTCCAGCCATGGGCCATCATCATCATCATCACCATCACCATCACGGC AGCGCAGAAATCGGTACTGGCTTTC
Halo TEV 3xGS BamHI Stop XhoI - Rev	TACTTCCAGCTCGAGTTAGGATCCGGAACCAGAGCCGGAACCCTGGAAGT ACAGGTTTTCGCTGCCAGTGGTTGGCTCGCCGGAAATCTCCAGCGTCGACAG CCAGCGCG
ASPP1 CTD BamHI - For	TACTTCCAGGGATCCAACCCCCCTGGCACTGCTC
ASPP1 CTD Stop XhoI - Rev	TACTTCCAGCTCGAGTTAGGCGAGTGTTCGCTGTCG
ASPP2 CTD BamHI - For	TACTTCCAGGGATCCAACCCCCCTTGCTTTACTGCTAG
ASPP2 CTD Stop XhoI - Rev	TACTTCCAGCTCGAGTTAGGCCAAGCTCCTTTGTCTTG
iASPP CTD BamHI - For	TACTTCCAGGGATCCCTGAACCCGCTGGTTCTG
iASPP CTD Stop XhoI - Rev	TACTTCCAGCTCGAGTTACTAAACTTTAGAACGCTGCGG
iASPP N813A Y814A - For	GGCTACGTGCCGCGGGCCGCCTTCGGGCTGTTCCCCAGG
iASPP N813A Y814A - Rev	CCTGGGGAACAGCCCGAAGGCGGCCCGCGGCACGTAGCC

Table S2. ITC fit parameters. Related to Figure 6

Component A (cell)	Component B (syringe)	K_D (95% CI) [μM]	ΔH (95% CI) [kcal/mol]	IncfA (95% CI)	IncfB (95% CI)
iASPP CTD	JunD peptide	4.575 (4.353 to 4.808)	-9.277 (-9.443 to -9.120)	0.079 (0.068 to 0.090)	0
ASPP1 CTD	JunD peptide	n.d.	n.d.	n.d.	n.d.
ASPP2 CTD	JunD peptide	98.328 (87.078 to 111.517)	-4.334 (-4.674 to -4.041)	0	0
iASPP CTD N813A Y814A	JunD peptide	n.d.	n.d.	n.d.	n.d.
iASPP CTD	JunD pT71 peptide	n.d.	n.d.	n.d.	n.d.
