# Supplementary material for: Mutant Ras and inflammation-driven skin tumorigenesis is suppressed via a JNK-iASPP-AP1 axis
Source: Cell Rep. 2022 Oct 18;41(3):111503. doi: 10.1016/j.celrep.2022.111503 (PMC9597577; doi:10.1016/j.celrep.2022.111503)
Supplement: Document S2. Article plus supplemental information [file mmc2.pdf]

Cell Reports

Article

Mutant Ras and inflammation-driven skin tumorigenesis is suppressed via a JNK-iASPP-AP1 axis

Graphical abstract

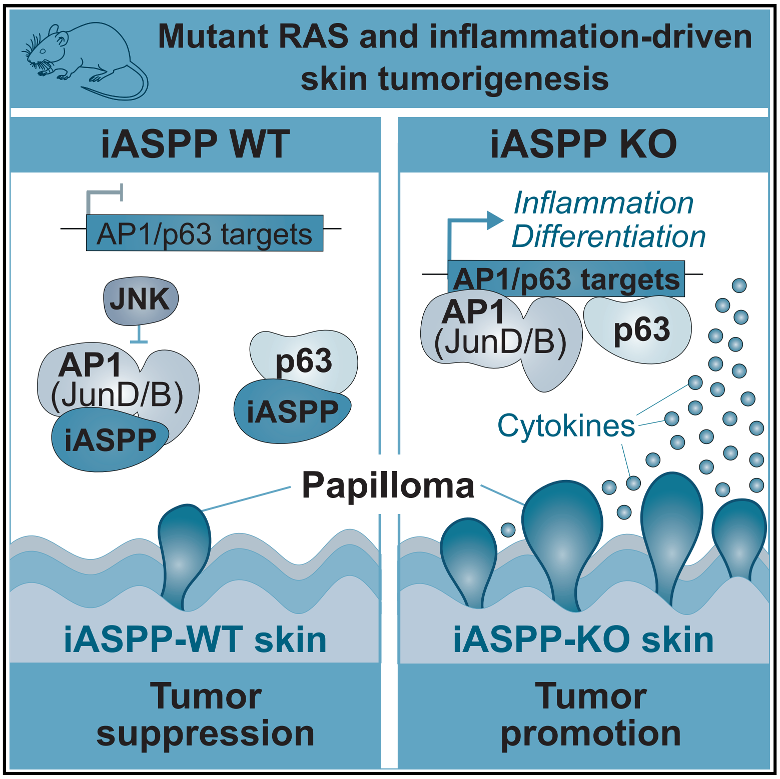

Highlights

- iASPP is a tumor suppressor of DMBA/TPA-driven mouse skin tumorigenesis
- iASPP controls selective transcription of AP1 and p63 target genes in keratinocytes
- JNK activation disrupts iASPP-JunD complexes that are mediated by PxxP/SH3 binding
- iASPP is a regulator of the AP1 signaling pathway

Authors

Khatoun Al Moussawi, Kathryn Chung, Thomas M. Carroll, ..., Huiqing Zhou, Volker Dötsch, Xin Lu

Correspondence

xin.lu@ludwig.ox.ac.uk

In brief

Al Moussawi et al. show that iASPP, a known p53 inhibitor, suppresses inflammation-driven skin tumorigenesis *in vivo*. Mechanistically, iASPP binds JunD via PxxP/SH3 interaction and forms a JNK-iASPP-AP1 axis that selectively represses the expression of a subset of AP1 and p63 target genes involved in skin differentiation and inflammation.

Al Moussawi et al., 2022, Cell Reports *41*, 111503
October 18, 2022 © 2022 The Authors.
https://doi.org/10.1016/j.celrep.2022.111503

CellPress

Cell Reports

CellPress
OPEN ACCESS

Article

Mutant Ras and inflammation-driven skin tumorigenesis is suppressed via a JNK-iASPP-AP1 axis

Khatoun Al Moussawi,[1,10] Kathryn Chung,[1,10] Thomas M. Carroll,[1,10] Christian Osterburg,[2,10] Artem Smirnov,[1,10] Rebecca Lotz,[2] Paul Miller,[1] Zinaida Dedeić,[1,9] Shan Zhong,[1] Martin Oti,[3] Evelyn N. Kouwenhoven,[3] Ruth Asher,[4,5] Robert Goldin,[6] Michael Tellier,[7] Shona Murphy,[7] Huiqing Zhou,[3,8] Volker Dötsch,[2] and Xin Lu[1,11,*]

[1]Ludwig Institute for Cancer Research, Nuffield Department of Clinical Medicine, University of Oxford, Oxford OX3 7DQ, UK
[2]Institute of Biophysical Chemistry and Center for Biomolecular Magnetic Resonance, Goethe University, Frankfurt, Germany
[3]Radboud University, Department of Molecular Developmental Biology, Faculty of Science, Radboud Institute for Molecular Life Sciences, Nijmegen, the Netherlands
[4]Cellular Pathology, John Radcliffe Hospital, Oxford OX3 9DU, UK
[5]Department of Histopathology, University Hospital Wales, Cardiff CF14 4XW, UK
[6]Department of Pathology, Imperial College London, Faculty of Medicine at St Mary's, Norfolk Place, London W2 1PG, UK
[7]Sir William Dunn School of Pathology, University of Oxford, Oxford OX1 3RE, UK
[8]Radboud University Medical Centre, Department of Human Genetics, Radboud Institute for Molecular Life Sciences, 6500 Nijmegen, the Netherlands
[9]Present address: Department of Oncology, University of Oxford, Oxford OX3 7DQ, UK
[10]These authors contributed equally
[11]Lead contact
*Correspondence: xin.lu@ludwig.ox.ac.uk
https://doi.org/10.1016/j.celrep.2022.111503

SUMMARY

Concurrent mutation of a RAS oncogene and the tumor suppressor p53 is common in tumorigenesis, and inflammation can promote RAS-driven tumorigenesis without the need to mutate p53. Here, we show, using a well-established mutant RAS and an inflammation-driven mouse skin tumor model, that loss of the p53 inhibitor iASPP facilitates tumorigenesis. Specifically, iASPP regulates expression of a subset of p63 and AP1 targets, including genes involved in skin differentiation and inflammation, suggesting that loss of iASPP in keratinocytes supports a tumor-promoting inflammatory microenvironment. Mechanistically, JNK-mediated phosphorylation regulates iASPP function and inhibits iASPP binding with AP1 components, such as JUND, via PXXP/SH3 domain-mediated interaction. Our results uncover a JNK-iASPP-AP1 regulatory axis that is crucial for tissue homeostasis. We show that iASPP is a tumor suppressor and an AP1 coregulator.

INTRODUCTION

Acquisition of an activating RAS signaling pathway (EGFR, BRAF, KRAS) mutation and loss of tumor suppression, commonly caused by p53 mutation or deletion, is a central paradigm in carcinogenesis. Inflammation enables mutant RAS to bypass the requirement for loss of tumor suppression to drive tumorigenesis. In a well-established mouse skin tumor model, the chemical carcinogen 7,12-dimethylbenz(a)anthracene (DMBA) induces H-Ras mutation in keratinocytes, and the tumor-promoting agent TPA (12-O-tetradecanoylphorbol-13-acetate) induces inflammation to facilitate tumorigenesis. In this model, papillomas often occur without genetic alteration of the *TP53* (p53) gene. Surprisingly, wild-type (WT) p53 facilitates papilloma initiation, although loss of p53 promotes papilloma-to-carcinoma conversion (Kemp et al., 1993). The underlying mechanisms remain largely unknown.

The master transcription factor of squamous epithelium is p63, a member of p53 family, mainly expressed in the proliferative basal and supra-basal epithelial cells. Inherited point mutations in the p63 DNA binding domain cause human congenital disorders, such as ectrodactyly ectodermal dysplasia clefting and ankyloblepharon-ectodermal dysplasia-cleft lip/palate syndrome due to deregulated keratinocyte differentiation and abnormal squamous epithelium stratification (Brunner et al., 2002). Overexpression of ΔNp63, an N-terminal truncated isoform of p63, often occurs in squamous epithelial cancers of the skin, head, and neck (Lo Muzio et al., 2005; Smirnov et al., 2019). Unlike p53, p63 mutations are a rare event in human cancers; the molecular cause for this remains unknown.

Although p63 shares high sequence similarity with p53 in the DNA binding domain, binding a similar consensus sequence (De Laurenzi et al., 2000), only a small group of genes (such as *CDKN1A* (p21)) are transcriptionally regulated by all p53 family

This is an open access article under the CC BY license (http://creativecommons.org/licenses/by/4.0/).

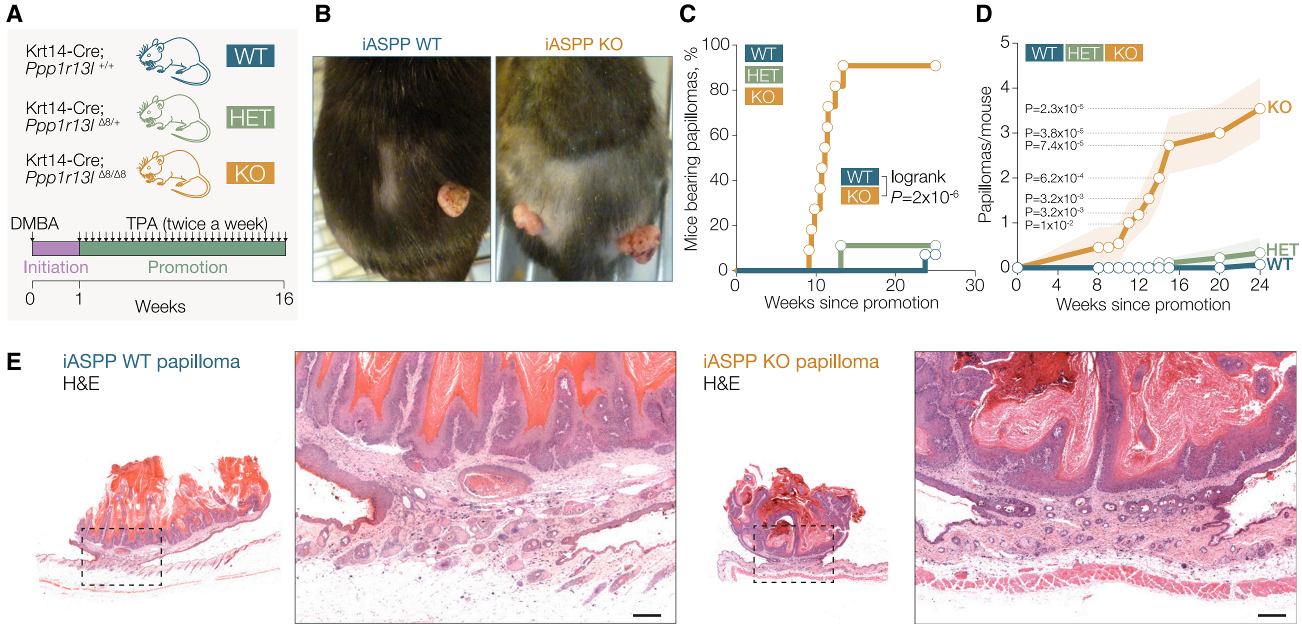

Figure 1. iASPP suppresses skin tumorigenesis driven by chemically induced mutant Ras and inflammation

(A) Diagram of the DMBA/TPA two-stage tumor induction protocol. *Krt14*-Cre;*Ppp1r13l*$^{+/+}$, *Krt14*-Cre;*Ppp1r13l*$^{\Delta8/+}$, and *Krt14*-Cre;*Ppp1r13l*$^{\Delta8/\Delta8}$ mice are abbreviated as WT, HET, and KO, respectively. Arrows indicate treatment time points.

(B) Representative images showing a dorsal view of papillomas on WT and KO mice.

(C) Papilloma incidence represented as a percentage of mice bearing papillomas in iASPP WT (7%, n = 14), HET (11%, n = 9), and KO (91%, n = 11) mice.

(D) Mean number of papillomas per mouse in WT, HET, and KO mice during DMBA/TPA treatment. Line plot shows mean values, shading represents SEM.

(E) H&E staining of papillomas in iASPP WT and KO mice. Absence of invasion into dermis is shown on enlarged images. Scale bar, 200 μm.

For p calculations for (C) and (D), see STAR Methods.

members in cells and *in vivo*, with most targets specific to individual p53 family members (Osada et al., 2005; Testoni et al., 2006). Like many transcription factors influencing cell fate, transcriptional target selection by p53 family members is regulated by cellular factors that are cell context dependent. Many p53 binding proteins exist, and the evolutionarily conserved ASPP protein family (ASPP1, ASPP2, and iASPP) was identified as the first family able to regulate p53 target selection (Bergamaschi et al., 2003, 2004; Samuels-Lev et al., 2001). Due to its ability to bind the DNA binding domains of p63 and p73, iASPP is also one of the few identified common regulators of p53 family members (Robinson et al., 2008). Experimentally, iASPP deletion causes cellular senescence *in vitro* (Chikh et al., 2011; Notari et al., 2011). The importance of the iASPP/p63 complex is supported by *in vivo* results in animals and humans. iASPP deletion causes abnormal hair growth in mice and cattle, and skin abnormalities in humans (Dedeić et al., 2018; Falik-Zaccai et al., 2017; Herron et al., 2005; Simpson et al., 2009). *In vivo*, iASPP deletion in keratin 14-expressing keratinocytes causes mouse squamous epithelium abnormalities, and phenotypes such as open eyelids at birth and delayed wound healing (Dedeić et al., 2018). In normal human skin, nuclear iASPP co-localizes with p63 in human basal epithelial cells and abnormal skin develops in patients carrying defective *PPP1R13L* (encoding iASPP) (Falik-Zaccai et al., 2017; Notari et al., 2011; Robinson et al., 2019). We hypothesized that part of iASPP's function in skin may be mediated through its ability to bind p63 and regulate transcriptional target selection.

iASPP is an inhibitor of NF-κB (Ge et al., 2021; Herron et al., 2005; Hu et al., 2015; Yang et al., 1999); a key driver of inflammatory signaling pathways and a suppressor of skin tumorigenesis (Dajee et al., 2003). As iASPP is a known p53 inhibitor, an important regulator of p63 in skin homeostasis, and an inhibitor of NF-κB, we hypothesized that iASPP activity may affect how mutant RAS and inflammation drives tumorigenesis. Here, we identify a crucial role of iASPP in regulating selective transcription of p63 and AP1 targets, and show that iASPP coordinates crosstalk between TGFα/EGFR/JNK and p53/p63 pathways to maintain tissue homeostasis, leading to pathogenesis when dysregulated.

RESULTS

iASPP suppresses skin tumorigenesis driven by chemically induced mutant Ras and inflammation

iASPP was specifically deleted in keratinocytes by crossing *Ppp1r13l*$^{flox\Delta8/flox\Delta8}$ mice with cytokeratin 14 promoter-linked Cre-expressing mice (*Krt14*-Cre), as described previously (Dedeić et al., 2018). The resulting mutant mice, *Krt14*-Cre;*Ppp1r13l*$^{+/+}$, *Krt14*-Cre;*Ppp1r13l*$^{\Delta8/+}$, and *Krt14*-Cre;*Ppp1r13l*$^{\Delta8/\Delta8}$, are referred to as iASPP-WT, iASPP-HET, and iASPP-KO, respectively. These mice were treated with topical DMBA, followed by twice-weekly doses of TPA for 15 weeks (Figure 1A). DMBA induces specific and irreversible activating mutations in the *Hras* proto-oncogene, and TPA, an activator of protein kinase C (Arcoleo and Weinstein, 1985),

CellPress
OPEN ACCESS

induces inflammation, promoting tumorigenesis. After treatment, we observed significantly more papillomas in iASPP-KO than in iASPP-WT animals (Figures 1B–1D) and *Hras* mutations were confirmed (Figure S1A). Almost all iASPP-KO mice developed exophytic papillomas, with higher incidence and earlier onset than iASPP-WT or heterozygous animals (Figures 1C and 1D). DMBA/TPA treatment induced nuclear p53 and iASPP at basal layers of papillomas (Figures S1B and S1C). All examined papillomas were benign, with no signs of epidermal invasion. Underlying muscle layers (Figure 1E) were well differentiated, expressing differentiation markers, such as loricrin, and were covered with extensive layers of keratin (Figure S1D). Of note, iASPP-KO papillomas showed increased numbers of cells strongly positive for differentiation markers keratin 1 (CK1) and involucrin (Figure S1E). Papillomas also showed infiltration of immune cells, such as macrophages (F4/80), neutrophils (MPO), T cells (CD3), and B cells (CD45R) (Figure S1F). These results show that, in contrast to its oncogenic property, iASPP is a paradoxical suppressor of tumorigenesis driven by mutant Ras and inflammation.

Loss of iASPP selectively enhances p63 binding to regions containing AP1 motifs

To gain insight into how iASPP suppresses papilloma formation, we used primary mouse keratinocytes with tamoxifen-inducible iASPP deletion, derived from *Ppp1r13l*$^{flox/flox}$;Cre^{+}ER^{T} mice. Absence of iASPP expression after tamoxifen treatment was confirmed by IF staining (Figure 2A). Bulk RNA-sequencing was conducted from mouse primary keratinocytes with or without tamoxifen treatment. Using an adjusted p value threshold of 0.05, we identified 867 and 969 genes with significantly higher expression in iASPP-WT and in iASPP-KO keratinocytes, respectively, while expression of 13,484 genes remained unchanged (Figure 2B).

Gene set enrichment analysis (GSEA) using the gene ontology-biological process (GO-BP) and Hallmark collections showed that gene sets enriched in iASPP-KO cells were strongly associated with keratinization, inflammation, and metabolism, whereas gene sets related to cell division and E2F targets were among the top pathways enriched in iASPP-WT cells (Figure S2A). Given the potential link with E2F signaling, we also conducted GSEA using the C3 Legacy TFT database, which lists genes with highly conserved TF motifs in promoter regions (Figure S2B). Genes with E2F motifs in promoter regions were strongly enriched among iASPP-WT cells. Genes with AP1/bZip motifs, including a TRE (TPA responsive element)-like site (TGANTCA) and a CRE (cAMP response element)-like site (TCANNTGAY), were the only motifs significantly enriched among upregulated genes in iASPP KO. Genes with promoter motifs related to NF-κB, a transcription factor shown to be inhibited by iASPP (Yang et al., 1999), did not show significant upregulation following iASPP KO in this analysis.

The GO-BP keratinization gene set, and its parent ontology keratinocyte differentiation, contain many genes expressed in the upper epidermal layers. Many of these keratinocyte differentiation genes had significantly higher expression in iASPP-KO cells, including upper epidermal genes, such as *Sppr2d*, *Sprr2e*, and *S100a7a*, which were among the top 50 upregulated genes in iASPP-KO cells (Figures 2B, S2C, and S2D). These findings emphasize iASPP's key role in negatively regulating the transcription of keratinization-related genes, consistent with previous studies showing that iASPP deficiency facilitates cell-cycle arrest and cellular senescence *in vitro* and enhances squamous differentiation *in vivo* (Chikh et al., 2011; Notari et al., 2011).

As the TF p63 is the master regulator of epidermal proliferation, differentiation, and homeostasis, and iASPP binds the DNA binding region of p63, we examined how iASPP may influence p63 genome binding and target regulation by performing p63 ChIP-seq in iASPP-WT and iASPP-KO keratinocytes (Figure 2A). We identified 20,922 p63-bound loci across both samples, most of which (17,160) were not affected by the presence or absence of iASPP (Figure 2C). However, using MAnorm differential binding analysis with a significance threshold of 5×10^{-3}, we identified 1,877 and 1,885 loci with significantly enriched p63 binding in iASPP-WT and iASPP-KO cells, respectively (Figure 2C).

The p63 motifs found in iASPP-WT- and iASPP-KO-enriched peaks were highly similar (Figure S2E), suggesting that iASPP did not directly alter p63 sequence selectivity. We therefore tested whether iASPP affects p63 target selectivity by modulating interactions with other TFs. We extracted the DNA sequence from each p63-bound peak region and scored them for the presence of all 579 TF motifs in the JASPAR CORE 2018 database, based on the maximum similarity of each sequence to the canonical motifs in this database. Examining the association between TF motif scores and the changes in p63 binding signal for these same peak regions in iASPP KO cells showed that motif scores from the AP1 family had the greatest correlation with increased p63 binding in iASPP KO (Figure 2D). Motif scores from p53 family members and NF-κB were not strongly correlated with changes in p63 binding (Figure 2D). This suggested that p63-bound sequences containing AP1 motifs were most likely to show increased p63 binding following iASPP depletion.

We assessed whether the link between AP1 and p63 binding enrichment in iASPP KO keratinocytes was dependent on p63 motif strength (Figure S2F). Among peaks containing a weak underlying p63 motif (i.e., bottom quintile of p63 motif scores), iASPP-KO-enriched peaks were more likely than iASPP-WT-enriched peaks to have a significant AP1 (JUNB) motif (49% versus 12%). A similar trend was noted for peaks with p63 motifs scoring in the top quintile, but with substantially less difference between groups (31% of iASPP-KO peaks versus 17% of iASPP-WT peaks) (Figure S2F). Thus, AP1 may be important to help mediate p63 binding to target sequences containing weaker p63 motifs, and this mode of binding at weak p63 sites containing AP1 motifs is particularly sensitive to the presence or absence of iASPP.

iASPP depletion affects expression of epidermal differentiation genes

To integrate the ChIP-seq and transcriptomic findings, we assigned each p63 peak to the nearest gene and examined the subset of peaks annotated to genes expressed in our mouse keratinocyte RNA-seq data. A small proportion of p63-bound peaks (12.4%) were located within 2 kb of a transcription start site

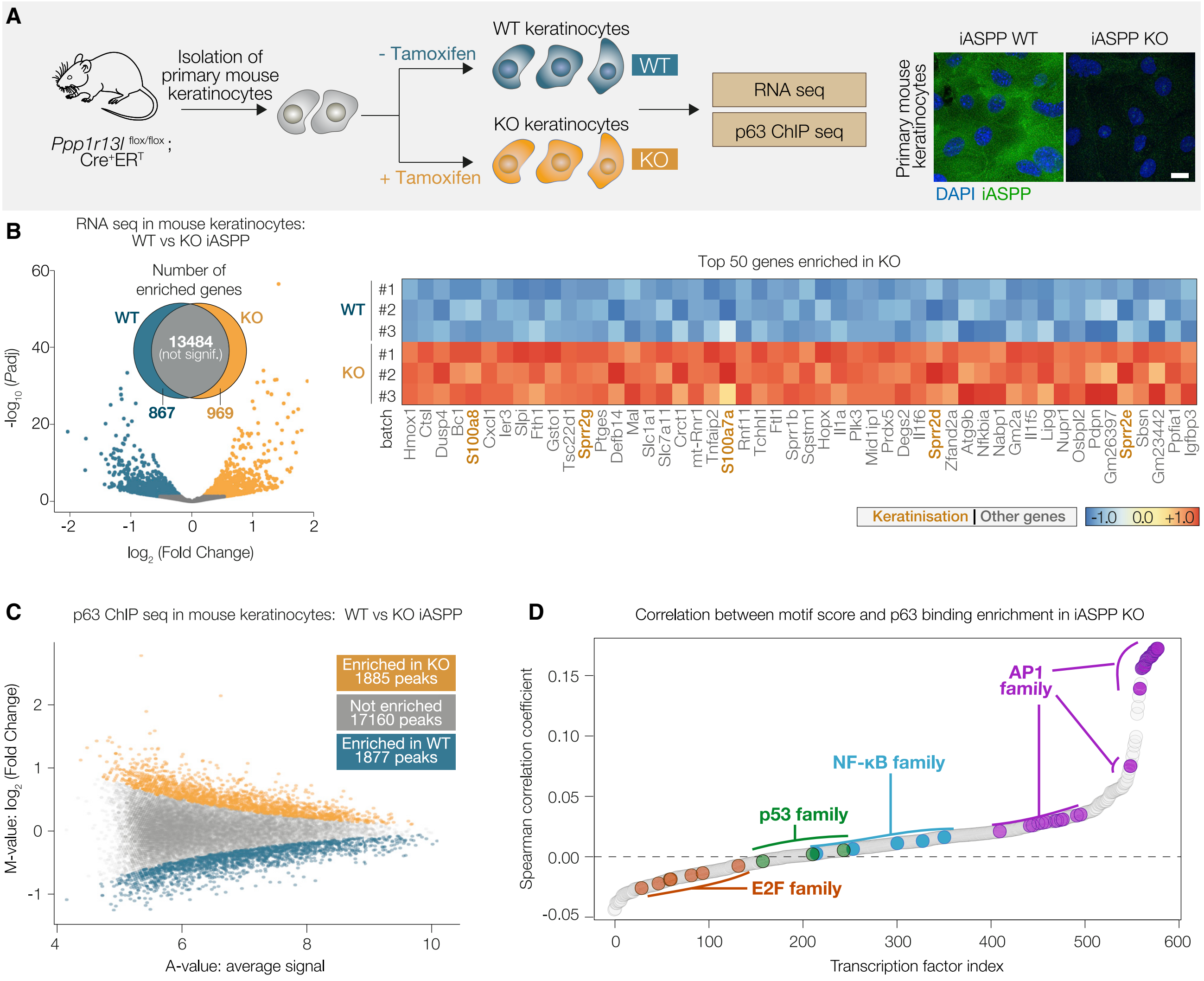

Figure 2. Loss of iASPP selectively enhances p63 binding to regions containing AP1 motifs

(A) Left, study workflow diagram. Right, IF staining of iASPP in WT and KO primary mouse keratinocytes. Scale bar, 10 μm.

(B) Left, in the volcano plot, genes are assigned a color depending on whether they are determined to be significant (adjusted $p < 0.05$) and in which condition they are significantly enriched. Right, heatmap showing scaled VST-normalized counts for the 50 genes most significantly enriched in iASPP KO. In orange: five genes from the GO-BP keratinocyte differentiation pathway.

(C) MA plot showing the average signal (A values) and log_2-transformed fold changes (M values) for each p63 peak. Using MAnorm-derived p with a significance threshold of 5×10^{-3}, we defined each peak as not enriched (gray) or significantly enriched in iASPP KO (orange) or WT (blue).

(D) Dot plot showing the correlation between TF motif scores (FIMO) and enhanced p63 binding in iASPP KO. Among all 579 JASPAR 2018 CORE vertebrate motifs, AP1 (MA0490.1/JUNB) and E2F1 motifs have the highest and lowest association scores, respectively. p53 and NF-κB show intermediate ranking.

(TSS), whereas 40.7% were in regions 2–20 kb from a TSS, and 46.9% were associated with distal elements located over 20 kb from the TSS (Figure 3A). Genes bound by p63 peaks were significantly more likely to be upregulated in iASPP KO cells than the reference set of all expressed genes (odds ratio [OR] = 1.3, $p = 2.67 \times 10^{-6}$), suggesting that p63 targets were more likely to be upregulated by iASPP depletion (Figure S3A). Of these p63-bound genes, those associated with peaks showing significantly increased p63 binding (MA_{norm} $p < 5 \times 10^{-3}$) after iASPP deletion had a particularly high likelihood of upregulation (OR = 1.82, $p = 2.01 \times 10^{-9}$) (Figure S3A). Genes with p63 peaks without differential p63 binding had a weaker, but still significant link with upregulation after iASPP depletion (OR = 1.32, $p = 8.11 \times 10^{-7}$), whereas genes with p63 peaks that increased in iASPP-WT cells were not significantly linked with transcriptional upregulation (OR = 1.17, p = 0.252) (Figure S3A).

Genes with p63 peaks within 20 kb of the TSS that contained an AP1 motif in the peak region were significantly more likely to be upregulated after iASPP KO (OR = 1.73, $p = 4.32 \times 10^{-10}$) (Figure S3B). In addition, genes with p63 peaks within 20 kb of the TSS containing an AP1 motif but no detectable p63 motif were strongly associated with increased gene expression (OR = 2.13, $p = 3.03 \times 10^{-5}$) (Figure S3C). In these regions,

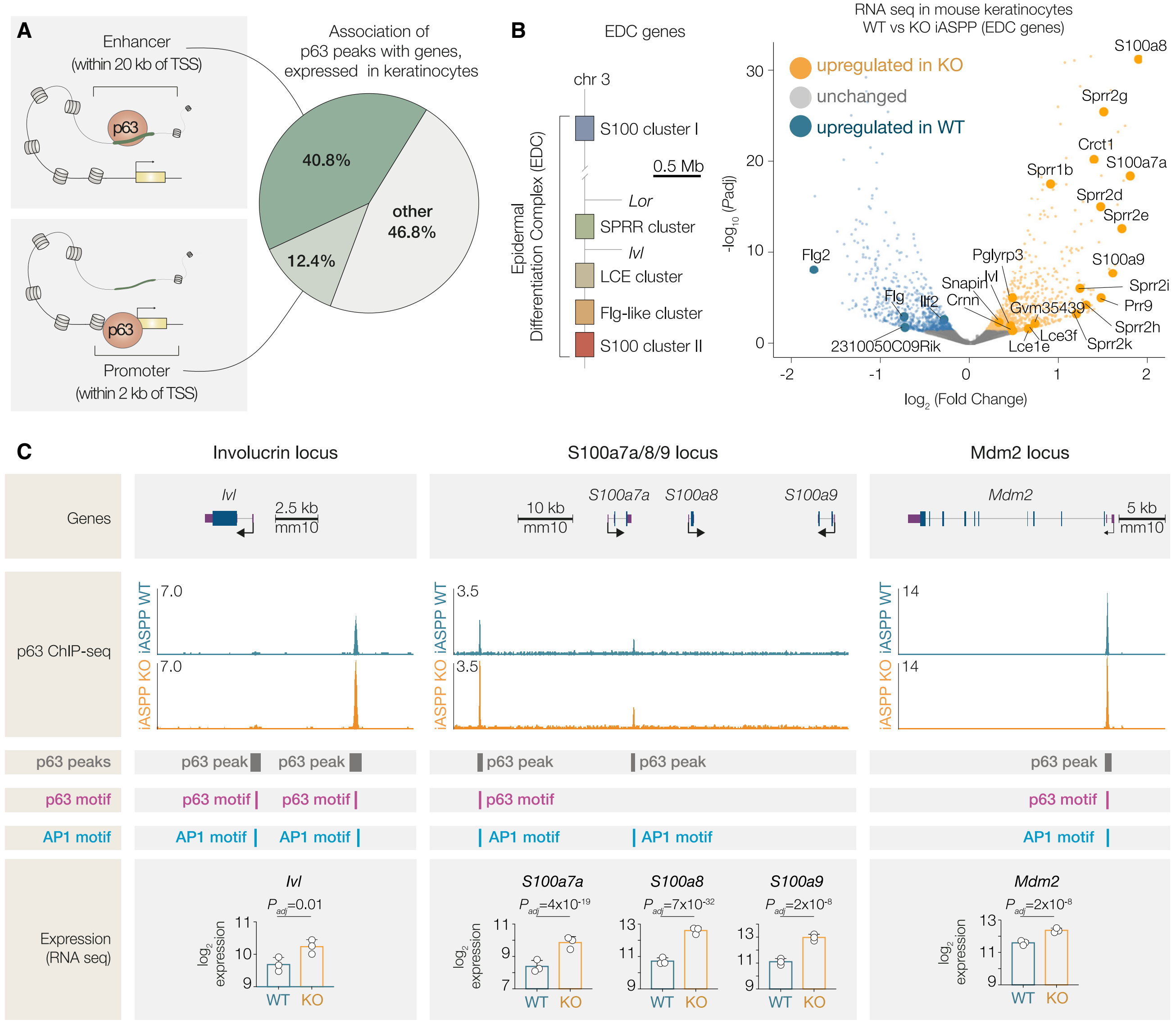

Figure 3. iASPP depletion affects expression of epidermal differentiation genes

(A) Peaks annotated to expressed genes were categorized in regions according to their distance from TSS: promoter region (within 2 kb either side of TSS), enhancer region (outside of promoter but within 20 kb upstream or downstream), or other. Pie chart shows the percentage of peaks in each region.

(B) Left, diagram of the mouse EDC. Right, volcano plot with significantly differentially expressed EDC genes after iASPP KO labeled.

(C) p63 binding (by ChIP-seq) and TF motifs (FIMO) at example peak regions as indicated. ChIP-seq signal values are presented as signal per million reads. VST-transformed RNA-seq expression values for the highlighted genes are shown below the genome schematic. Adjusted p values for expression were calculated by DESeq2 (see STAR Methods for calculation).

p63 may bind to a non-canonical p63 motif that did not meet the significance thresholds used in this study, or p63 binding may be indirect, presumably through interaction with other TFs, such as AP1. Therefore, similarly to the ChIP-seq analysis, the presence of iASPP showed the greatest transcriptional impact on gene targets bound by p63 at sites that contained AP1 motifs but not strong p63 motifs.

Notably, several genes in the epidermal differentiation complex (EDC), a gene-rich locus on chromosome 3 containing a number of genes controlling epidermal development, were strongly upregulated in iASPP-KO keratinocytes (Figure 3B). Genes within the EDC were much more likely to be upregulated upon iASPP depletion compared with all genes (OR = 4.42, $p = 2.42 \times 10^{-12}$) (Figure S3D), and we found similar results for the subset of EDC genes bound by p63 within 20 kb of the TSS (OR = 4.12, $p = 5.51 \times 10^{-4}$; Figure S3D), suggesting that iASPP may interfere in particular with p63 transactivational activity in the EDC. For two exemplar EDC regions bound by p63 (enhancers of genes encoding involucrin [*Ivl*] and the S100-family members *S100a7a/S100a8/S100a9*), iASPP deletion resulted in both significantly increased p63 binding and significantly increased gene expression (Figure 3C). For both targets, AP1

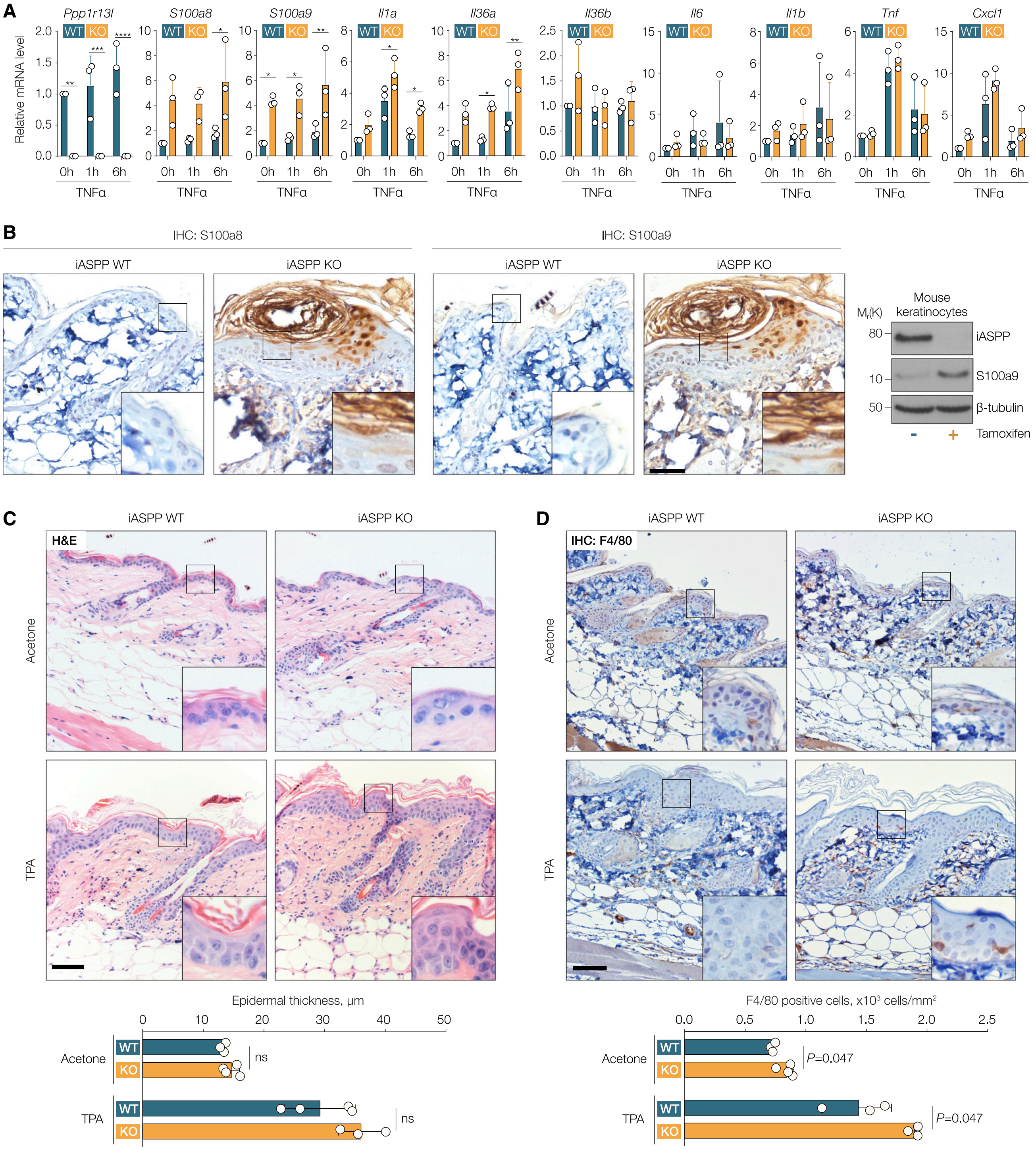

Figure 4. iASPP deficient keratinocytes induce pro-inflammatory gene expression *in vitro* and attract macrophages *in vivo*

(A) qPCR analysis of mRNA expression levels of inflammatory genes (and iASPP gene *Ppp1r13l*) in iASPP WT and KO primary keratinocytes at 0, 1, and 6 h after treatment with TNF-α. Values are mean + SD. *p < 0.05, **p < 0.01, ***p < 0.001, ****p < 0.0001; n = 3 biological replicates.

(B) Left, IHC staining of S100a8 and S100a9 in untreated skin sections from iASPP WT and KO mice (adjacent sections). Scale bar, 50 μm. Right, IB of iASPP and S100a9 expression levels in iASPP WT (−tamoxifen) and KO (+tamoxifen) primary keratinocytes.

(legend continued on next page)

motifs are present in the p63 binding region. By contrast, for *Mdm2*, a target of p53 family members that is not involved in keratinocyte differentiation, no impact on p63 genome occupancy at the *Mdm2* promoter peak was observed, despite upregulation of RNA expression of this gene. To assess whether the AP1 pathway is also activated in the DMBA/TPA model used in our study, we tested expression of AP1 members JunB, cJun, and JunD in papilloma samples by immunohistochemistry (IHC) and confirmed strong nuclear staining of all three proteins (Figure S3E). Together, iASPP depletion generally results in enrichment of p63 genome occupancy at binding sites containing AP1 motifs, leading to increased expression of target genes around these loci. In keratinocytes, this modulation of p63 genome binding by iASPP particularly impacts the EDC, which likely contributes to the previously reported role of iASPP in modulating squamous differentiation.

iASPP-deficient keratinocytes induce pro-inflammatory gene expression *in vitro* and attract macrophages *in vivo*

S100a8 and *S100a9* are well-known pro-inflammatory genes (Wang et al., 2018), and inflammatory gene sets were among the most upregulated following iASPP depletion (Figure S2A). Several genes in inflammatory pathways were among the top 50 upregulated genes following iASPP depletion, including key inflammatory mediators, such as *S100a8*, *Cxcl1*, *Il1a*, and *Il1f6/Il36a* (Figure S4A). To validate a link between iASPP status and inflammation, we treated iASPP-WT and iASPP-KO keratinocytes with TNF-α, a potent pro-inflammatory cytokine, for 1 or 6 h. The levels of mRNA for *Il1a*, *Il36a*, *S100a8*, and *S100a9* were significantly higher in iASPP-KO cells compared with WT at 6 h after TNF-α stimulation, whereas there were no significant changes in expression levels of *Il36b*, *Il6*, *Il1b*, *Tnf*, and *Cxcl1*. In addition, the *S100a9* expression level was significantly increased in iASPP-KO cells, even in the absence of TNF-α treatment (Figure 4A). Greater expression of S100a9 protein in iASPP-KO cells was shown by immunoblotting (IB) of iASPP-WT and iASPP-KO keratinocytes (Figure 4B). IHC staining of adjacent sections from untreated iASPP-KO skin sections with anti-S100a8 and anti-S100a9 antibodies showed increased expression in certain skin regions. In contrast, S100a8 and S100a9 were hardly detectable in iASPP-WT mouse skin sections (Figure 4B). These data support the idea that iASPP impedes the expression of a subset of pro-inflammatory genes, including S100a8 and S100a9, in mouse keratinocytes *in vitro* and *in vivo*.

S100a8 and S100a9 affect keratinocyte proliferation and influence the skin microenvironment *in vivo* when secreted by keratinocytes (Nukui et al., 2008). In our model, iASPP is only deleted in keratin-14-expressing keratinocytes, thus we can examine how iASPP may influence skin homeostasis and inflammatory responses in a cell-intrinsic or -extrinsic manner. iASPP-WT and iASPP-KO mice were treated with TPA or acetone (solvent control for TPA) (Figure S4B). Histological examination of H&E-stained mouse skin sections showed no significant difference in dermal cellularity and interfollicular epidermis (IFE) thickness between acetone-treated iASPP-WT and iASPP-KO skin (Figure 4C). In the TPA-treated cohort, epidermal hyperplasia was clearly visible, indicating cutaneous inflammation. A small increase in IFE thickness was observed in TPA-treated iASPP-KO skin versus TPA-treated iASPP-WT mouse skin. However, the difference was not statistically significant (iASPP-WT mean = 29.3 μm versus iASPP-KO mean = 36.0 μm, $p = 0.145$, Figure 4C). To measure the impact of iASPP depletion on skin cell proliferation *in vivo*, BrdU was injected into acetone- or TPA-treated iASPP-WT and iASPP-KO mice 1 h before termination. IF staining of BrdU on skin sections showed similar numbers of BrdU-positive cells per length of the keratin-14-postive IFE (number/mm) in acetone-treated iASPP-WT and iASPP-KO mice. An increase in BrdU incorporation within the IFE was detected upon TPA treatment. TPA-treated iASPP-deficient IFE showed a slightly higher, but not statistically significant, frequency of BrdU-positive cells per length of basal IFE than the WT (Figure S4C). Terminal deoxynucleotidyl transferase dUTP nick end labeling to detect apoptotic events on these skin sections (Figure S4C) showed no significant difference per length of IFE (number/mm) in WT versus iASPP-KO in acetone- or TPA-treated groups.

To investigate whether iASPP status in keratin-14-expressing keratinocytes influences the tissue microenvironment, mouse skin sections after TPA treatment were stained with a panel of immune cell markers. Short-term TPA treatment of iASPP-WT mice dorsal skin induced an infiltration of macrophages (F4/80) and neutrophils (MPO) (Figures 4D and S4D), and a smaller increase in the number of mast cells (toluidine blue) and blood vasculature (CD31) (Figures S4E and S4F). iASPP-KO mice showed increased infiltrating F4/80+ macrophages in both acetone- and TPA-treated groups (Figure 4D). iASPP status had no significant effect on neutrophil infiltration or distribution of blood vasculature. IF staining to examine the infiltration of adaptive immune cell populations showed that iASPP-deficient skin contained an increased number of CD45R+ B cells per skin area (number/mm^2) relative to WT acetone-treated skin, and a smaller increase relative to WT in TPA-treated skin (Figure S4G). iASPP status did not seem to affect numbers of infiltrating CD3+ T or CD8+ T cells per skin area in acetone- or TPA-treated skin (Figures S4H and S4I). Together, this suggests that iASPP depletion leads to upregulation of a subset of pro-inflammatory genes, such as S100a8 and S100a9, *in vitro* and *in vivo*. iASPP status alone has minimal impact on skin homeostasis. However, in response to inflammatory stimuli, iASPP deficiency in keratinocytes significantly enhances infiltrating innate immune cells, especially macrophages. These data are consistent with TPA as a potent inducer of AP1 and *S100a8* and *S100a9* being transcriptional targets of AP1 (Zenz et al., 2007).

(C) H&E analysis of acetone- or TPA-treated skin sections from iASPP WT and KO mice. Scale bar, 50 μm. Histograms below show epidermal thickness in the same samples. Values are mean ± SD. n = 3 (WT) and n = 4 (KO) mice in acetone cohort; n = 4 (WT), n = 3 (KO) mice in TPA cohort.

(D) IHC staining of F4/80-positive macrophages in acetone- or TPA-treated skin sections from iASPP WT and KO mice. Scale bar, 50 μm. Histograms below show quantification of the same samples. Values are mean + SD. Same cohort as in (C).

See STAR Methods for p calculations for (A), (C), and (D).

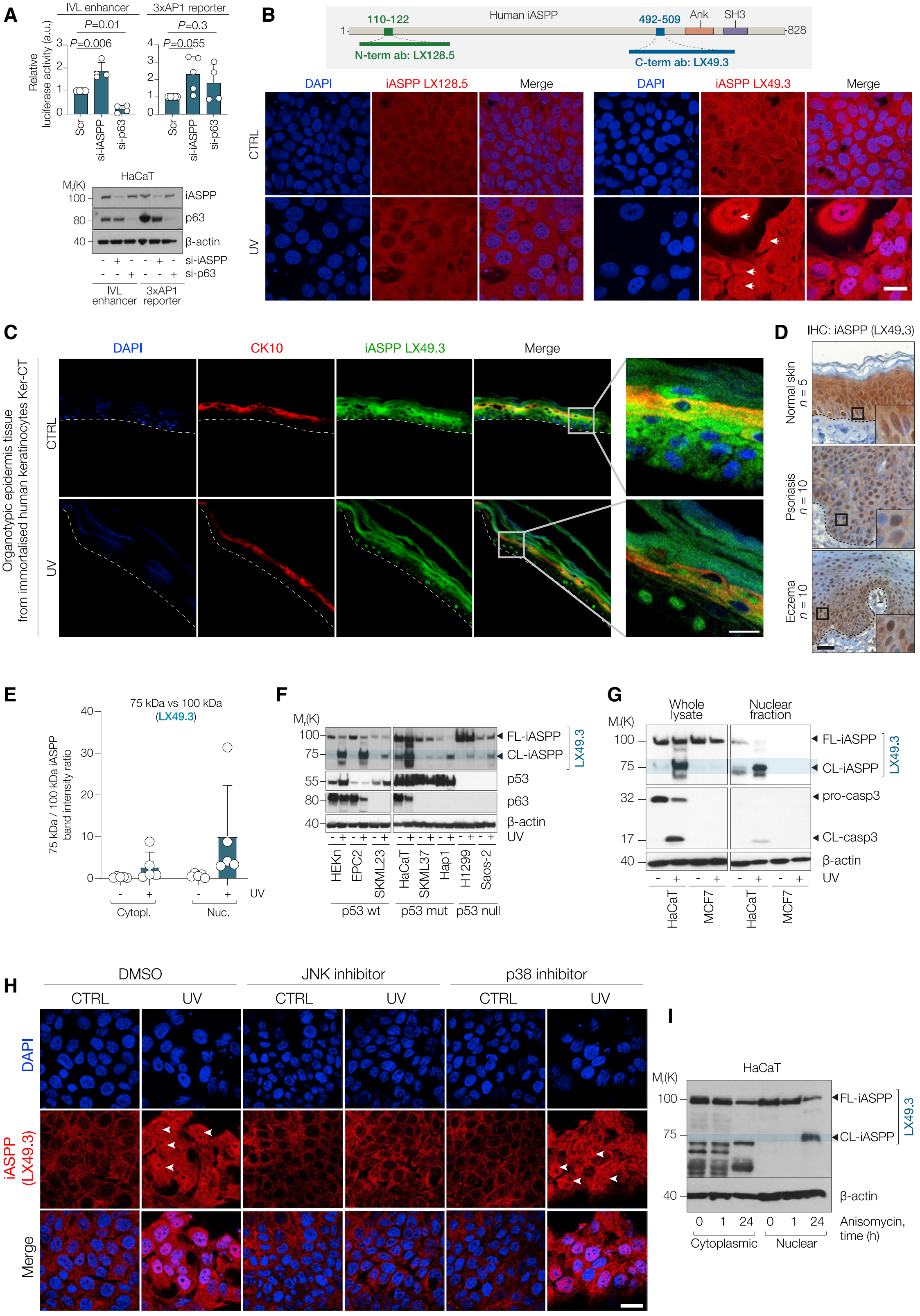

(legend on next page)

CellPress
OPEN ACCESS

Hence, iASPP may influence skin inflammatory responses via alteration of the microenvironment *in vivo*.

Cross-regulation between iASPP and JNK/AP1

To investigate how iASPP influences p63 and AP1 transcriptional activity at their co-regulated targets, we used a p63-bound region from the enhancer of the involucrin gene (*Ivl*) containing both a p63 and an AP1 motif. We subcloned the 600 bp region around the p63 peak, located 5.8 kb upstream of the *Ivl* TSS, into a pGL3-promoter luciferase vector containing a minimal SV40 promoter, generating a reporter to measure enhancer activity at this region upstream of involucrin (IVL-luc). In a human keratinocyte cell line, HaCaT, iASPP depletion by RNAi enhanced IVL-luc luciferase activity; whereas, under the same conditions, knockdown of p63 resulted in repressed IVL-luc activity (Figure 5A). Further, using a well-known AP1 reporter containing three repeated AP1 binding motifs (3xAP1-luc) in HaCaT cells, we observed a 2.3-fold increase in 3xAP1-luc luciferase after iASPP knockdown compared with scrambled control on average, although this increase was not significant after correction for multiple testing (adjusted p = 0.055). Under the same conditions, si-p63 showed a less significant increase in 3xAP1-luc activity (Figure 5A).

In both mouse and human keratinocytes, iASPP is mainly detected in the cytoplasm. Our previous studies showed that N-terminal truncated iASPP enters the nucleus via the RaDAR nuclear import pathway (Lu et al., 2014; Slee et al., 2004) and that nuclear iASPP is associated with metastasis (Lu et al., 2013). We thus tested whether AP1 signaling pathways influence iASPP's cellular localization and function. UV is one of the most important natural inducers of N-terminal Jun kinase (JNK) and AP1 signaling and a potent skin carcinogen. We treated HaCaT cells with increasing doses of UV and examined iASPP expression by IF staining at various time points (Figures S5A and S5B). Nuclear iASPP was detected by LX49.3 anti-iASPP antibody, which recognizes amino acids 492–509 near the iASPP C terminus. A dose-response and kinetics study showed that nuclear iASPP is present in most cells 24 h post UV exposure at 5 mJ/cm^2 or above (Figures S5A and S5B). Interestingly, an anti-iASPP antibody, LX128.5, which recognizes a dephosphorylated region within the 110–122 aa epitope at the N terminus of iASPP (Lu et al., 2013), mainly detected cytoplasmic iASPP 24 h after 5 mJ/cm^2 UV exposure (Figure 5B). Similar results were found in primary human keratinocytes (HEKn) and two p53 null human cancer cell lines, H1299 (non-small cell lung carcinoma) and Saos-2 (osteosarcoma) (Figure S5C). UV also induced nuclear localization of iASPP in basal layer cells of organotypic epidermal tissue derived from immortalized keratinocytes (Ker-CT) (Figure 5C) and HaCaT cells (Figure S5D). We also analyzed iASPP localization in human keratinocytes affected by inflammation using skin sections from healthy donors, or psoriasis or eczema patients. While high levels of iASPP expression were detected in both the cytoplasm and nucleus of keratinocytes in all normal skin sections, keratinocytes in psoriasis and eczema samples mainly expressed nuclear iASPP. This expression pattern was common to all affected skin biopsies examined (Figures 5D and S7).

To understand how UV induces nuclear iASPP, we subfractionated HaCaT cells with or without the indicated treatments and immunoblotted them with LX128.5 and LX49.3 antibodies (Figure S5E). UV only had a small impact on the level of full-length iASPP (~100 kDa, detected by both LX128.5 and LX49.3 antibodies). UV mostly induced the accumulation of a shorter 75 kDa fragment, detected by LX49.3 but not LX128.5. In all nuclear fractions tested, the iASPP 75 kDa/100 kDa ratio is >1, often >5, after UV irradiation (Figure 5E). In contrast, in cytoplasmic fractions, the 75/100 kDa ratio is close to 1 or less. IB of lysates of HaCaT cells depleted of iASPP by RNAi confirmed the specificity of both antibodies (Figure S5E). UV can induce 75 kDa nuclear iASPP in a panel of eight cell lines, irrespective of their p63 expression level and p53 mutation status (Figure 5F) indicating that UV induces 75 kDa nuclear iASPP independent of p63 and p53.

iASPP is a known substrate of effector caspases, such as caspase-3, with a molecular weight of around 75 kDa for cleaved iASPP product (Hu et al., 2015). In addition, UV activates caspase-3 activity (Park and Jang, 2014). HaCaT cells were thus treated with ZVAD, a pan-caspase inhibitor, or VX-765, a selective inhibitor of caspase-1/4, in the presence or absence of UV. ZVAD but not VX-765 was able to prevent UV from inducing 75 kDa iASPP. Detection of 75 kDa iASPP is associated with the intensity of cleaved activated caspase-3, and is

Figure 5. Cross-regulation between iASPP and JNK/AP1

(A) Upper, luciferase assay in HaCaT cells using involucrin enhancer reporter and 3xAP1 reporter after knockdown of iASPP or p63. Luciferase luminescence was normalized over renilla luminescence. Values are mean fold change over scramble + SD. For p calculation, see STAR Methods. Lower, IB of iASPP and p63 expression in the same samples.

(B) Upper, diagram of iASPP structure and epitopes of anti-iASPP antibodies LX49.3 and LX128.5 as indicated. Lower, IF staining of iASPP in HaCaT cells ± 5 mJ/cm^2 UV irradiation, stained with either LX49.3 or LX128.5 as indicated. Arrow indicates nuclear iASPP. Scale bar, 10 μm.

(C) IF staining of iASPP (LX49.3) and cytokeratin 10 (CK10) in organotypic epidermis tissue from immortalized Ker-CT cells. Tissues were collected 24 h post 150 mJ/cm^2 UV irradiation. Scale bar, 25 μm.

(D) IHC analysis of iASPP expression in a sample of human skin from healthy donors or patients with psoriasis or eczema. Scale bar, 50 μm.

(E) Barplot showing ratio of intensity of 75/100 kDa iASPP bands determined by IB using LX49.3 antibody in HaCaT cells upon UV irradiation and subjected to subcellular fractionation. Values are mean ratio + SD, n = 5 biological replicates.

(F) IB of iASPP, p53, and p63 expression levels in p53 wild-type (HEKn, EPC2, SKML23), p53 mutant (HaCaT, SKML37, Hap1), or p53 null (H1299, Saos-2) cell lines upon UV irradiation. FL-iASPP and CL-iASPP refer to full-length and cleaved iASPP, respectively.

(G) IB of iASPP and caspase-3 expression levels in whole lysates or nuclear fractions of HaCaT and MCF7 cells 24 h ± 5 mJ/cm^2 UV irradiation.

(H) IF staining of iASPP localization in HaCaT cells pre-treated with either JNK inhibitor or p38 inhibitor for 1 h and collected 24 h ± 5 mJ/cm^2 UV irradiation as indicated. Arrow heads indicate nuclear iASPP. Scale bar, 10 μm.

(I) IB of iASPP expression in cytoplasmic and nuclear fractions of HaCaT cells treated with JNK activator anisomycin for 1 or 24 h.

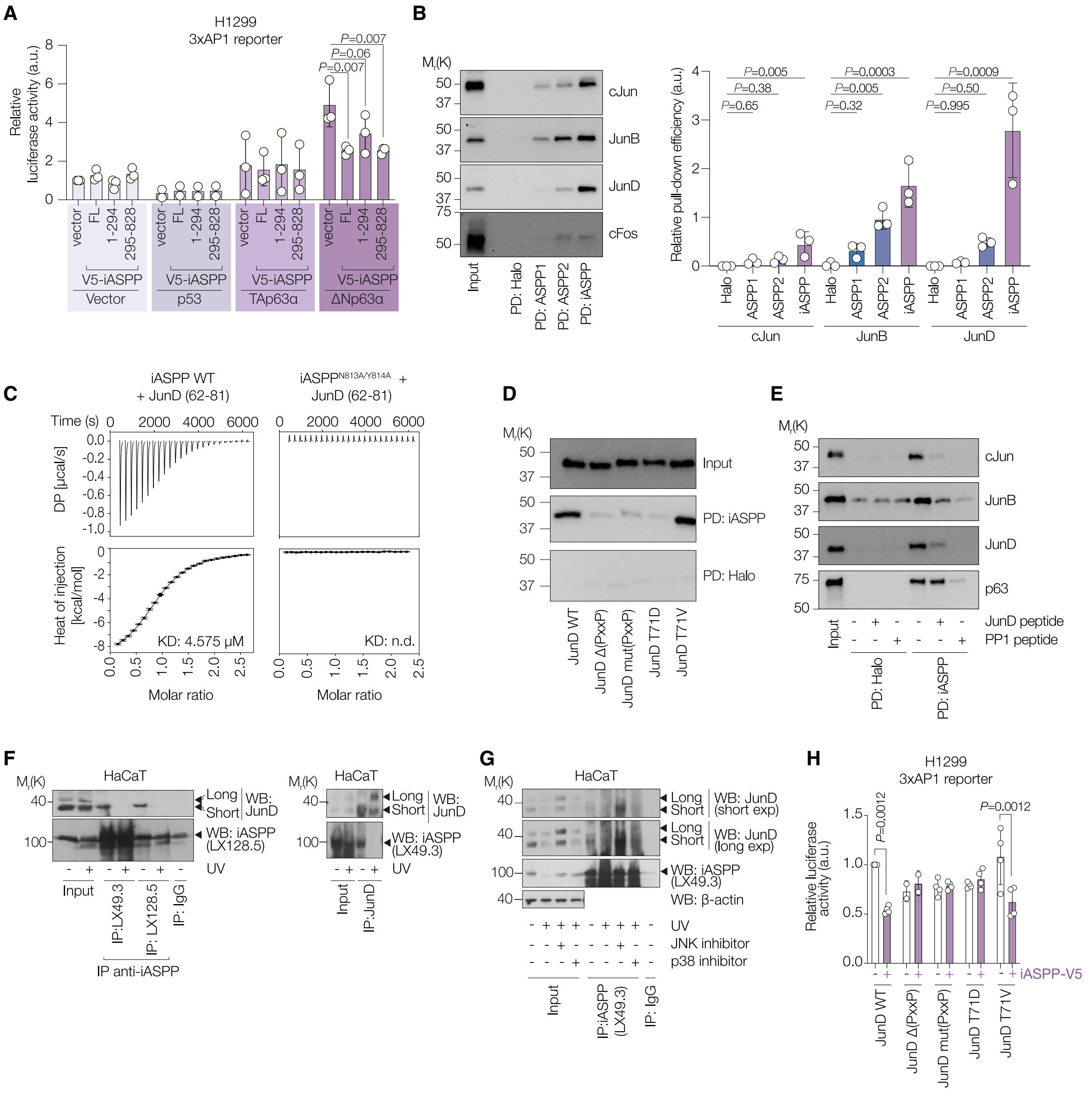

Figure 6. iASPP SH3 domain interacts with JunD N-terminal PxxP motif to inhibit AP1 transcriptional activity

(A) Luciferase activity assay in H1299 cells using 3xAP1 reporter after overexpression of FL-tagged (1–295) or V5-tagged (295–828) iASPP combined with p53, HA-tagged TAp63α, or HA-tagged ΔNp63α. Luciferase activity was normalized over renilla activity. Values are mean fold change over empty vector ± SD.

(B) Left, IB of pull down assay using immobilized Halo-tagged ASPP CTDs to pull down *in vitro* translated (IVT) Myc-tagged AP1 proteins. PD, pull down. Right, barplot showing the mean PD efficiency relative to input determined by densitometric quantification of the IB from left panel. Values are mean ± SD. n = 3 biological replicates.

(C) Isothermal titration calorimetry (ITC) results of JunD peptide binding to WT iASPP CTD (left) or iASPP CTD N813A/Y814A mutant (right). Top diagrams show raw titration profiles and bottom diagrams show integrated heat. Best fit of single-site binding model is shown as a solid black line with the resulting equilibrium binding constant (KD). Fit parameters are found in Table S2.

(D) IB of pull down assay using Halo-tagged iASPP CTD to pull down IVT Myc-tagged JunD WT and mutants.

(E) IB of pull down assay using Halo-tagged iASPP CTD to pull down IVT Myc-tagged cJun, JunB, JunD, or p63 ± of either JunD peptide or PP1 peptide.

(F) Left, co-IP using anti-iASPP antibodies in lysates of control or UV-irradiated HaCaT cells. IB for JunD. Right, co-IP using anti-JunD antibody in lysates of control or UV-irradiated HaCaT cells. IB for iASPP (LX49.3). Short and Long refer to short and long forms of JunD, respectively.

(legend continued on next page)

amplified in the presence of proteosome inhibitor MG132 (Figure S5F). Unlike HaCaT and other cell lines examined above, UV did not lead to the accumulation of 75 kDa iASPP in cytoplasmic or nuclear fractions in MCF7 cells, a caspase-3-deficient human breast cancer cell line (Figure 5G). Expression of myc-tagged WT caspase-3 but not the catalytically dead caspase-3 (C163A) mutant in MCF7 cells produced detectable amounts of 75 kDa iASPP (Figure S5G). These data demonstrate that UV induces a caspase cleaved 75 kDa product, which we call CL-iASPP.

To test the potential involvement of JNK activity in UV-induced nuclear accumulation of cleaved CL-iASPP, HaCaT cells were pre-treated with DMSO, a JNK inhibitor (JNK inhibitor VIII), or p38 kinase inhibitor (SB-202190) before UV exposure. Treatment with the JNK inhibitor but not p38 inhibitor prevented UV-induced nuclear iASPP (Figure 5H). IB confirmed that pre-treatment with the JNK but not p38 inhibitor significantly impaired UV induction of 75 kDa CL-iASPP (Figure S5H). A JNK activator, anisomycin, induced the accumulation of 75 kDa CL-iASPP (Figure 5I). JNK-induced iASPP cleavage is not human specific as similar results were also obtained in a mouse keratinocyte cell line XB2 (Figure S5I). These results suggest a regulation loop between iASPP and the JNK/AP1 pathway.

iASPP SH3 domain interacts with JunD N-terminal PxxP motif to inhibit AP1 transcriptional activity

iASPP may inhibit the transcriptional activity of AP1 directly by binding to AP1 components or indirectly via iASPP's ability to bind and inhibit the transcriptional activity of p63, since a p63-JunB complex has been reported previously (Sundqvist et al., 2020). Full-length p63 (TAp63α), expressed in skin at low levels, is inactive due to its dimeric structure and closed conformation (Deutsch et al., 2011), whereas the most expressed p63 isoform is an active N-truncated ΔNp63α, featuring an alternative transactivation domain to TAp63α. We previously showed that D294 is the caspase-3 cleavage site that generates a 75 kDa iASPP fragment (Hu et al., 2015). We tested the ability of overexpressed full-length iASPP or iASPP fragments containing amino acids 1–294 or 295–828 to inhibit AP1 activity in H1299 cells using a 3xAP1 luciferase reporter. H1299 is null for p53 and the p63 expression level is low (Sethi et al., 2015) or undetectable (Figure 5F), but it has WT endogenous iASPP (note: it is not possible to generate iASPP null cell lines). Expression of full-length or fragments of iASPP, p53, or TAp63α had minimal impact on 3xAP1 luciferase reporter activity, while expression of ΔNp63a-induced 3xAP1 luciferase activity. Co-expression of full-length or truncated iASPP reduced ΔNp63α-induced 3xAP1 activity (Figures 6A and S6A). These observations suggest that iASPP may directly inhibit AP1 activity.

Several AP1 components, such as JunD, contain a PxxP motif at their N terminus and iASPP contains a classic SH3 domain in its C-terminal domain (CTD) that interacts with PxxP motifs. We incubated recombinant iASPP CTD fused to Halo-Tag with *in vitro* translated JunD, JunB, c-Jun, and c-Fos (Figure 6B). All three Jun family members but not c-Fos were pulled down with iASPP CTD. Based on the amount of input, the proportion of JunD in complex with iASPP was higher compared with JunB and c-Jun. Under the same conditions, ASPP1 and ASPP2 CTD were less effective at binding to Jun family members (Figure 6B).

The JunD N terminus contains a classic PxxP motif, whereas JunB and c-Jun N-terminal regions do not. Unlike iASPP, ASPP1 and ASPP2 contain an atypical SH3 domain (Bergamaschi et al., 2006), suggesting that iASPP may form a complex with JunD via classic SH3/PxxP motif binding. To test this, we purified the isolated CTDs of ASPP1, ASPP2, and iASPP, including their SH3 domains, and synthesized a peptide with the PxxP motif of JunD (62–81 aa). A direct interaction between iASPP and JunD was confirmed by isothermal titration calorimetry (Figure 6C), whereas ASPP1 or ASPP2 CTDs failed to bind JunD (Figure S6B). Y814 in the iASPP SH3 domain is a highly conserved classic SH3 domain residue known to contact proline in PxxP motifs, and is absent in ASPP1 and ASPP2 (Bergamaschi et al., 2006). Mutation of Y814 and its adjacent residue N813 prevented iASPP-JunD binding (Figure 6C), confirming the importance of the SH3 domain. To investigate the specificity of the interaction for the JunD PxxP motif, we generated a series of full-length JunD mutants: deleted PxxP motif (69–74 aa; Δ-xxP); mutated PxxP (P69A, P72A, R74A; mutPxxP); phosphomimic T71D; and a non-phosphorylatable T71V mutation, as T71 is within the PxxP motif and is predicted to be a potential JNK site (Figure 6D). The PxxP and phosphomimic JunD mutants failed to bind iASPP, whereas JunD T71V bound as effectively as the WT, supporting a direct SH3-PxxP binding between iASPP and non-phosphorylated JunD. We also tested whether the iASPP-JunD interaction can be competed with either a JunD peptide or a well-characterized PxxP-containing high-affinity binding peptide derived from the phosphatase PP1, able to bind iASPP SH3 domain (Bertran et al., 2019). The PP1 peptide effectively inhibited interactions between iASPP and p63, JunD, JunB, or c-Jun (Figure 6E). The JunD peptide significantly reduced iASPP binding to JunD, JunB, and c-Jun but not to p63 (Figure 6E), suggesting that iASPP may have a higher binding affinity for p63 than Jun family members, or have a second interaction site with p63.

The iASPP/JunD complex was detected in untreated HaCaT cells using two different anti-iASPP antibodies, LX49.3 and LX128.5 (Figure 6F, left). Importantly, iASPP failed to complex with JunD in UV-treated HaCaT cells, and this was not due to reduced iASPP or JunD expression (Figure 6F). Similarly, using an anti-JunD antibody, the iASPP/JunD complex was only detected in untreated HaCaT cells (Figure 6F, right). Both anti-iASPP antibodies pulled down JunB in untreated HaCaT cells,

(G) Co-IP using LX49.3 in lysates of control or UV-irradiated HaCaT cells treated with DMSO, JNK inhibitor, or p38 inhibitor. IB for JunD. Short and Long refer to short and long forms of JunD respectively.
(H) Luciferase activity of WT JunD and indicated JunD mutants ± iASPP in H1299 cells using 3xAP1 reporter as a readout. Luciferase activity was normalized over renilla activity. Values are mean fold change over empty vector ± SD. n = 4 biological replicates.
For p calculation for (A), (B), and (H), see STAR Methods.

while less JunB was pulled down in UV-treated cells, possibly due to a lower level of JunB or a change in interactions. UV caused a large increase in levels of c-Jun and c-Fos (Figure S6C). In the same experiment as that shown for iASPP/JunB and iASPP/JunD, the anti-iASPP antibody LX49.3 but not LX128.5 pulled down some c-Jun protein in UV-treated cells. However, c-Jun pull down was highly variable among experiments (see the table in Figure S6D for a summary). c-Fos failed to co-immunoprecipitate with iASPP under all conditions tested (Figure S6C). Lysates from subcellular fractionation of both human and mouse keratinocytes, HaCaT and IMOK, respectively, were used to immunoprecipitate iASPP using LX49.3 and LX128.5. LX128.5 robustly co-immunoprecipitated JunD or JunB in the nuclear fractionation lysates in both cell lines, whereas LX49.3 was less effective (Figure S6E), in agreement with our result from unfractionated lysates (Figure 6F). The specificity of JunD binding was confirmed by JunD knockdown (Figure S6F).

To test whether JNK activation may inhibit the iASPP/JunD complex, we treated HaCaT cells with a JNK inhibitor or a p38 inhibitor with or without UV treatment. iASPP failed to pull down JunD in UV-treated cells in the presence of p38 kinase inhibitor or the control agent DMSO. Importantly, addition of JNK inhibitor enabled detection of the iASPP/JunD complex in UV-treated cells (Figure 6G). These data demonstrate that JNK activation prevents iASPP/JunD interaction, explaining why the iASPP/JunD complex is not detected in UV-irradiated cells.

We tested the ability of iASPP to influence the transcriptional activities of JunD, JunB, and c-Jun using a 3xAP1 luciferase reporter in H1299 cells (Figure S6G). Consistent with our biochemical findings, iASPP had a greater inhibitory effect on the transcriptional activity of JunD compared with JunB and c-Jun, whereas the transcriptional activity of JunD with a mutated PxxP motif or phosphomimic T71D (as used in Figure 6D) was not repressed by iASPP (Figures 6H and S6H). Activity of JunD with the T71V mutation was repressed by iASPP. Together, our results suggest that iASPP inhibits AP1 activity directly by forming a complex with JunD through SH3/PxxP motif binding.

DISCUSSION

Our findings reveal two key functions for iASPP: as a tumor suppressor and as an AP1 coregulator (Figures 7A and 7B). The discovery that iASPP can act as a tumor suppressor is in contrast to previous findings that iASPP is an oncogene: (1) iASPP is an inhibitor of p53-induced apoptosis (Bergamaschi et al., 2003); (2) iASPP is required for cell proliferation as iASPP deficiency causes cellular senescence (Chikh et al., 2011); (3) iASPP inhibits keratinocyte differentiation (Notari et al., 2011); (4) iASPP is overexpressed in various cancer cells *in vitro* and *in vivo*, and nuclear iASPP associates with cancer metastasis (Lu et al., 2013); and (5) iASPP deletion induces p53-mediated cell death in cancer cells *in vitro* or reduced tumor growth *in vivo* in a xenograft mouse model (Lin et al., 2011; Wang et al., 2015).

Prior studies were mainly focused on cell-intrinsic roles of iASPP. In the *in vivo* skin tumor model here, interactions between keratinocytes and the tissue microenvironment are either tumor promoting or tumor suppressive. Skin has been called a second immune system, as keratinocytes can secrete cytokines, such as IL1-a and S100a8/S100a9 (Jiang et al., 2020). A subset of iASPP-repressed targets are pro-inflammatory genes, such as *Il1a*, *Il1f6*, *S100a8*, and *S100a9*, so, compared with iASPP WT, iASPP KO keratinocytes may secrete more pro-inflammatory cytokines, such as S100a8 and S100a9, attracting immune cells, including myeloid-derived suppressor cells, to form a tumor-promoting microenvironment (Cheng et al., 2008). Thus, iASPP depletion in keratinocytes may influence the microenvironment, which in turn has an effect on keratinocyte growth (e.g., promoting papilloma formation) (Figure 7A). While the loss of iASPP increased the papilloma onset and burden, iASPP-KO papillomas showed higher expression of differentiation markers (e.g., involucrin), suggesting a more "benign" tumor phenotype. This can be partially explained by activation of the p53 pathway in the absence of iASPP. Future studies are needed to establish whether the tumor-suppressive function of iASPP is p53 dependent or not.

AP1 was first identified as a TPA-activated transcription factor (Angel et al., 1987) and is also an important regulator of epidermal differentiation and skin tumor development (Eckert et al., 2013), consistent with iASPP being an inhibitor of AP1 and a suppressor of DMBA/TPA-induced tumorigenesis in mouse skin (Figure 7B). Given that NF-κB is also a key regulator in skin homeostasis and inflammation, and that iASPP was firstly identified as a binding partner of RelA and an inhibitor of NF-κB (Yang et al., 1999), it is interesting that, in our analysis, AP1 target genes were more affected than NF-κB targets by iASPP depletion and that iASPP affects p63 genome occupancy surrounding AP1 motifs rather than NF-κB targets. These findings suggest that iASPP-AP1 interactions may have distinct importance in p63-regulated squamous lineages, whereas iASPP-NF-κB may be important in other contexts.

Several AP1 components are expressed in the skin. For example, in epidermis, JunD is expressed in all cell layers, whereas c-Jun and JunB are more restricted, with expression levels changing during differentiation (Mehic et al., 2005). AP1 complexes, particularly those involving c-Jun or cFos, influence the transcriptional activity and function of p53 and its family members (Schreiber et al., 1999; Subramanian et al., 2015). Previous analysis of ChIP and expression data predicted that JunD cooperates with p63, modulating its ability to bind transcriptional targets in human keratinocytes (Sethi et al., 2014) by an unknown mechanism. Our findings that JunD is the Jun member that iASPP binds most robustly in human keratinocytes and that iASPP deficiency results in profound upregulation of a subset of p63 and AP1 target genes, suggest that iASPP may be a missing link connecting p63 and JunD (Figure 7B). p63 was recently reported to form a complex with JunB (Sundqvist et al., 2020), and we also observed that iASPP can bind JunB. Hence, iASPP may alter multiple p63/AP1 co-regulatory complexes.

Mechanistically, our evidence suggests that iASPP regulates expression of a subset of AP1 target genes via direct binding to AP1 components, particularly to JunD. Interaction is via a type II SH3 domain interacting peptide (PxxP) in an AP1 subunit

CellPress
OPEN ACCESS

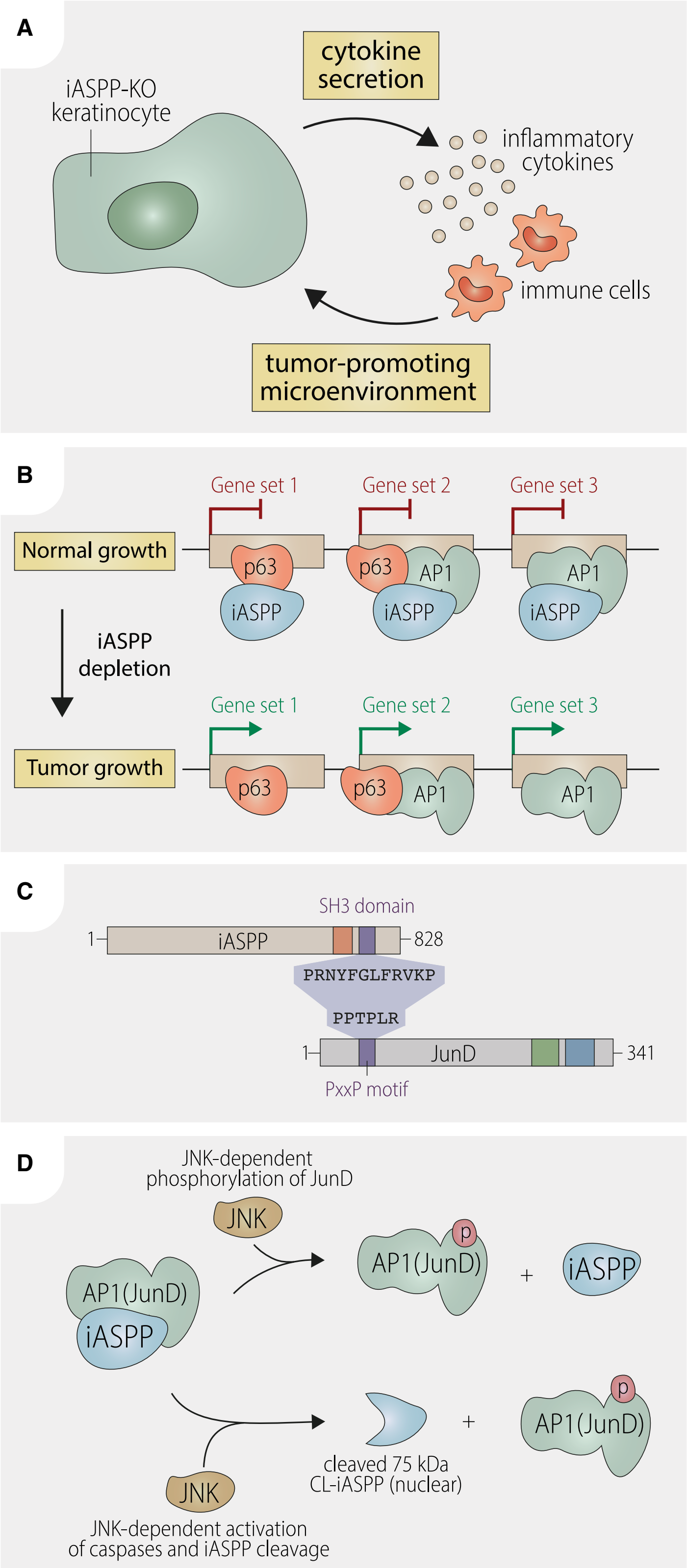

Figure 7. Proposed models of iASPP-mediated regulation of tumorigenesis and the JNK-iASPP-AP1 axis

Diagrams to show (A) how loss of iASPP in keratinocytes may enhance inflammation and create a pro-tumorigenic microenvironment; (B) how iASPP may regulate selective transcription of p63 and AP1 targets either alone or in combination; (C) iASPP SH3 domain and JunD PxxP motif mediate iASPP-JunD interaction; (D) regulatory loop of JNK/iASPP/AP1 axis.

and the SH3 domain of iASPP (Figure 7C). In JunD, the PxxP sequence is located N-terminal to the transactivation domain (Hirai et al., 1989). As the DNA binding domain of JunD and the leucine zipper dimerization domains are at the C-terminal end, transcriptional inhibition by iASPP is unlikely to be via block of DNA binding or dimerization. Instead, the proximity of iASPP binding to the transactivation domain suggests that steric hindrance of the transcriptional machinery or cofactors may be the relevant mechanism.

JunD is expressed in a wide range of cell types in addition to keratinocytes, so the iASPP/JunD interaction is likely to influence the target selectivity of other transcription factors that cooperate with JunD. Interestingly, JunD is generally considered a tumor suppressor, but has been reported as an inhibitor of p53-induced cellular senescence *in vitro* (Weitzman et al., 2000) and as an oncogene *in vivo* through its ability to cooperate with mutant RAS to drive lung tumorigenesis (Ruiz et al., 2021). Thus, the ability of iASPP to bind and inhibit JunD activity may contribute to iASPP's tumor-suppressive function in mutant RAS and inflammation-driven tumorigenesis. Environmental stimuli that induce JNK/AP1 and inflammation stimulate a tumor-promoting microenvironment and JNK is required for mutant RAS-driven tumorigenesis (Cellurale et al., 2011). Our finding that JNK activation can inhibit the iASPP/JunD interaction (Figure 7D) suggests that part of the tumor-promoting function of these stimuli and JNK activation may be achieved via the JNK-iASPP-AP1 axis, by preventing iASPP binding to JunD to suppress tumor growth.

In untreated keratinocytes, most iASPP is cytoplasmic but some can be detected in the nucleus. Shuttling of iASPP to the nucleus can be increased by cyclin B/CDK1 at G2M under normal physiological conditions (Lu et al., 2013). JNK activation can induce caspase-mediated cleavage of iASPP, and CL-iASPP enters the nucleus. Consistent with a role of iASPP in transcriptional regulation, our cellular fractionation data showed that iASPP/JunD and iASPP/JunB complexes can occur in the nucleus in untreated human and mouse keratinocytes. Phosphorylation of JunD mediated by JNK may prevent iASPP binding (Figure 7D), but further work is required to assess the extent to which this modulates transcriptional inhibition *in vivo*. We cannot exclude the possibility that a small amount of JunB and JunD may form complexes with iASPP in the cytoplasm, possibly being retained there. Monomeric Jun proteins can shuttle between the nucleus and cytoplasm (Malnou et al., 2007).

Finally, our discovery of a connection between iASPP and the JNK/AP1 pathways explains the previous observation that two spontaneous iASPP mutant mice (Wa3 and Woe2) (Herron et al., 2005; Toonen et al., 2012) have highly similar phenotypic features (eyelids open at birth, wavy hair) to TGF-α (Wa1), EGFR (Wa2), and JNK1/2 double-deficient mice (Luetteke et al., 1993, 1994; Weston et al., 2004). Since TGFα is the ligand of EGFR, and that JNK1/2 proteins are the downstream effectors of TGF-α/EGFR/RAS signaling, it is understandable that deficiency in any pathway component may result in overlapping phenotypes, and our results now link iASPP to this axis. The physical and functional interactions between p63 and the JNK/iASPP/AP1 axis are of great biological importance in tissue homeostasis and skin inflammation. Dysregulation of the JNK/iASPP/AP1 axis is likely to impact on many other pathological conditions beyond the skin.

Limitations of the study

This study has several limitations. First, due to restrictions of permitted animal experiments, it was impossible to assess the impact of iASPP on the conversion from benign papilloma to malignant cutaneous carcinomas. Second, some *in vivo* observations in the DMBA/TPA model may not reflect a human skin tumor setting, as mouse and human skin are not identical. Third, some of the iASPP/p63 targets identified in this study may be mouse specific, as only 40% of the human genome aligns to the mouse genome. Furthermore, promoter and enhancer regions are more diverse than coding regions. Combined RNA-seq and p63 ChIP-seq studies performed in human keratinocytes with or without iASPP expression will help identify evolutionarily conserved p63/iASPP targets between mouse and human.

STAR★METHODS

Detailed methods are provided in the online version of this paper and include the following:

- KEY RESOURCES TABLE
- RESOURCE AVAILABILITY
  - Lead contact
  - Materials availability
  - Data and code availability
- EXPERIMENTAL MODEL AND SUBJECT DETAILS
  - Mouse experiments and genotyping
  - DMBA/TPA treatment
  - Primary keratinocytes culture
  - Cell culture and treatments
  - Human epidermal organotypic culture
  - Human samples
- METHOD DETAILS
  - Western blot (IB)
  - IF and confocal microscopy
  - Luciferase activity assay
  - RNA interference
  - cDNA overexpression
  - Immunohistochemistry
  - Haematoxylin and eosin (H&E) staining
  - Toluidine blue staining for mast cells
  - mRNA extraction and qPCR
  - Library preparation and sequencing
  - Cloning and mutagenesis
  - IP
  - Protein expression and purification
  - Halo pulldown
  - Isothermal calorimetry
  - Bioinformatic analyses
- QUANTIFICATION AND STATISTICAL ANALYSIS

SUPPLEMENTAL INFORMATION

Supplemental information can be found online at https://doi.org/10.1016/j.celrep.2022.111503.

CellPress
OPEN ACCESS

ACKNOWLEDGMENTS

This research was mainly funded by the Ludwig Institute for Cancer Research. X.L. is partly funded by NIHR-Oxford Biomedical Research Centre (BRC). Deutsche Forschungsgemeinschaft, the Netherlands Organisation for Scientific Research, and the Wellcome Trust funded V.D., H.Z., and S.M., respectively. The views expressed are those of the author(s) and not necessarily those of the NHS, the NIHR, or the Department of Health. For the purpose of Open Access, the author has applied for a CC BY public copyright license to any Author Accepted Manuscript version arising from this submission.

AUTHOR CONTRIBUTIONS

K.C., Z.D., P.M., and S.Z. carried out animal experiments, K.A.M. carried out cell line experiments. A.S. performed organotypic tissue experiments, C.O. and R.L. performed biochemical experiments. Z.D., K.C., M.O., and E.N.K. performed RNA-seq and ChIP-seq experiments. T.M.C. and M.T. did bioinformatics analysis. R.A. sourced human skin samples. K.C. and R.G. analyzed histopathology. H.Z. and S.M. supervised E.N.K. and M.T., respectively. X.L. supervised the study. A.S., C.O., K.A.M., T.M.C., M.T., V.D., and X.L. wrote the manuscript and all authors read and approved the manuscript.

DECLARATION OF INTERESTS

X.L. is a scientific advisory board member of Oxford SimCell. T.M.C. is a founder, employee, and shareholder of a diagnostics company (Cleancard).

Received: December 26, 2021
Revised: June 29, 2022
Accepted: September 22, 2022
Published: October 18, 2022

REFERENCES

Amemiya, H.M., Kundaje, A., and Boyle, A.P. (2019). The ENCODE blacklist: identification of problematic regions of the genome. Sci. Rep. *9*, 9354. https://doi.org/10.1038/s41598-019-45839-z.

Andrews, S. (2010). FastQC: A Quality Control Tool for High Throughput Sequence Data. https://GitHub.com/s-andrews/FastQC.

Angel, P., Imagawa, M., Chiu, R., Stein, B., Imbra, R.J., Rahmsdorf, H.J., Jonat, C., Herrlich, P., and Karin, M. (1987). Phorbol ester-inducible genes contain a common cis element recognized by a TPA-modulated trans-acting factor. Cell *49*, 729–739. https://doi.org/10.1016/0092-8674(87)90611-8.

Arcoleo, J.P., and Weinstein, I.B. (1985). Activation of protein kinase C by tumor promoting phorbol esters, teleocidin and aplysiatoxin in the absence of added calcium. Carcinogenesis *6*, 213–217. https://doi.org/10.1093/carcin/6.2.213.

Bailey, T.L., and Birol, I. (2021). STREME: accurate and versatile sequence motif discovery. Bioinformatics *37*, 2834–2840. https://doi.org/10.1093/bioinformatics/btab203.

Benjamini, Y., and Hochberg, Y. (1995). Controlling the false discovery rate: a practical and powerful approach to multiple testing. J. Roy. Stat. Soc. B *57*, 289–300.

Bergamaschi, D., Samuels, Y., Jin, B., Duraisingham, S., Crook, T., and Lu, X. (2004). ASPP1 and ASPP2: common activators of p53 family members. Mol. Cell Biol. *24*, 1341–1350. https://doi.org/10.1128/mcb.24.3.1341-1350.2004.

Bergamaschi, D., Samuels, Y., O'Neil, N.J., Trigiante, G., Crook, T., Hsieh, J.-K., O'Connor, D.J., Zhong, S., Campargue, I., Tomlinson, M.L., et al. (2003). iASPP oncoprotein is a key inhibitor of p53 conserved from worm to human. Nat. Genet. *33*, 162–167. https://doi.org/10.1038/ng1070.

Bergamaschi, D., Samuels, Y., Sullivan, A., Zvelebil, M., Breyssens, H., Bisso, A., Del Sal, G., Syed, N., Smith, P., Gasco, M., et al. (2006). iASPP preferentially binds p53 proline-rich region and modulates apoptotic function of codon 72–polymorphic p53. Nat. Genet. *38*, 1133–1141. https://doi.org/10.1038/ng1879.

Bertran, M.T., Mouilleron, S., Zhou, Y., Bajaj, R., Uliana, F., Kumar, G.S., van Drogen, A., Lee, R., Banerjee, J.J., Hauri, S., et al. (2019). ASPP proteins discriminate between PP1 catalytic subunits through their SH3 domain and the PP1 C-tail. Nat. Commun. *10*, 771. https://doi.org/10.1038/s41467-019-08686-0.

Blighe, K., Rana, S., and Lewis, M. (2018). EnhancedVolcano: publication-ready volcano plots with enhanced colouring and labeling. https://github.com/kevinblighe/EnhancedVolcano.

Brautigam, C.A. (2015). Calculations and publication-quality illustrations for analytical ultracentrifugation data. In Analytical Ultracentrifugation, pp. 109–133. https://doi.org/10.1016/bs.mie.2015.05.001.

Brunner, H.G., Hamel, B.C.J., and Van Bokhoven, H. (2002). The p63 gene in EEC and other syndromes. J. Med. Genet. *39*, 377–381. https://doi.org/10.1136/jmg.39.6.377.

Buske, F.A., Bodén, M., Bauer, D.C., and Bailey, T.L. (2010). Assigning roles to DNA regulatory motifs using comparative genomics. Bioinformatics *26*, 860–866. https://doi.org/10.1093/bioinformatics/btq049.

Cellurale, C., Sabio, G., Kennedy, N.J., Das, M., Barlow, M., Sandy, P., Jacks, T., and Davis, R.J. (2011). Requirement of c-Jun NH2-terminal kinase for Ras-initiated tumor formation. Mol. Cell Biol. *31*, 1565–1576. https://doi.org/10.1128/mcb.01122-10.

Cheng, P., Corzo, C.A., Luetteke, N., Yu, B., Nagaraj, S., Bui, M.M., Ortiz, M., Nacken, W., Sorg, C., Vogl, T., et al. (2008). Inhibition of dendritic cell differentiation and accumulation of myeloid-derived suppressor cells in cancer is regulated by S100A9 protein. J. Exp. Med. *205*, 2235–2249. https://doi.org/10.1084/jem.20080132.

Chikh, A., Matin, R.N.H., Senatore, V., Hufbauer, M., Lavery, D., Raimondi, C., Ostano, P., Mello-Grand, M., Ghimenti, C., Bahta, A., et al. (2011). iASPP/p63 autoregulatory feedback loop is required for the homeostasis of stratified epithelia. EMBO J. *30*, 4261–4273. https://doi.org/10.1038/emboj.2011.302.

Coutandin, D., Osterburg, C., Srivastav, R.K., Sumyk, M., Kehrloesser, S., Gebel, J., Tuppi, M., Hannewald, J., Schäfer, B., Salah, E., et al. (2016). Quality control in oocytes by p63 is based on a spring-loaded activation mechanism on the molecular and cellular level. Elife *5*, e13909. https://doi.org/10.7554/eLife.13909.

Dajee, M., Lazarov, M., Zhang, J.Y., Cai, T., Green, C.L., Russell, A.J., Marinkovich, M.P., Tao, S., Lin, Q., Kubo, Y., and Khavari, P.A. (2003). NF-κB blockade and oncogenic Ras trigger invasive human epidermal neoplasia. Nature *421*, 639–643. https://doi.org/10.1038/nature01283.

Levrero, M., De Laurenzi, V., Costanzo, A., Gong, J., Wang, J.Y., and Melino, G. (2000). The p53/p63/p73 family of transcription factors: overlapping and distinct functions. J. Cell Sci. *113*, 1661–1670.

Dedeić, Z., Sutendra, G., Hu, Y., Chung, K., Slee, E.A., White, M.J., Zhou, F.Y., Goldin, R.D., Ferguson, D.J.P., McAndrew, D., et al. (2018). Cell autonomous role of iASPP deficiency in causing cardiocutaneous disorders. Cell Death Differ. *25*, 1289–1303. https://doi.org/10.1038/s41418-017-0039-6.

Deutsch, G.B., Zielonka, E.M., Coutandin, D., Weber, T.A., Schäfer, B., Hannewald, J., Luh, L.M., Durst, F.G., Ibrahim, M., Hoffmann, J., et al. (2011). DNA damage in oocytes induces a switch of the quality control factor TAp63α from dimer to tetramer. Cell *144*, 566–576. https://doi.org/10.1016/j.cell.2011.01.013.

Dobin, A., Davis, C.A., Schlesinger, F., Drenkow, J., Zaleski, C., Jha, S., Batut, P., Chaisson, M., and Gingeras, T.R. (2013). STAR: ultrafast universal RNA-seq aligner. Bioinformatics *29*, 15–21. https://doi.org/10.1093/bioinformatics/bts635.

Eckert, R.L., Adhikary, G., Young, C.A., Jans, R., Crish, J.F., Xu, W., and Rorke, E.A. (2013). AP1 transcription factors in epidermal differentiation and skin cancer. J Skin Cancer, 537028. https://doi.org/10.1155/2013/537028.

Falik-Zaccai, T.C., Barsheshet, Y., Mandel, H., Segev, M., Lorber, A., Gelberg, S., Kalfon, L., Ben Haroush, S., Shalata, A., Gelernter-Yaniv, L., et al. (2017). Sequence variation in PPP1R13L results in a novel form of cardio-cutaneous syndrome. EMBO Mol. Med. *9*, 319–336. https://doi.org/10.15252/emmm.201606523.

Frankish, A., Diekhans, M., Ferreira, A.-M., Johnson, R., Jungreis, I., Loveland, J., Mudge, J.M., Sisu, C., Wright, J., Armstrong, J., et al. (2019). GENCODE reference annotation for the human and mouse genomes. Nucleic Acids Res. *47*, D766–D773. https://doi.org/10.1093/nar/gky955.

Ge, W., Wang, Y., Zheng, S., Zhao, D., Wang, X., Zhang, X., and Hu, Y. (2021). Nuclear iASPP determines cell fate by selectively inhibiting either p53 or NF-κB. Cell Death Discov. *7*, 195. https://doi.org/10.1038/s41420-021-00582-1.

Goujon, M., McWilliam, H., Li, W., Valentin, F., Squizzato, S., Paern, J., and Lopez, R. (2010). A new bioinformatics analysis tools framework at EMBL-EBI. Nucleic Acids Res *38*. W695–699.

Herron, B.J., Rao, C., Liu, S., Laprade, L., Richardson, J.A., Olivieri, E., Semsarian, C., Millar, S.E., Stubbs, L., and Beier, D.R. (2005). A mutation in NFkB interacting protein 1 results in cardiomyopathy and abnormal skin development in wa3 mice. Hum. Mol. Genet. *14*, 667–677. https://doi.org/10.1093/hmg/ddi063.

Hirai, S.I., Ryseck, R.P., Mechta, F., Bravo, R., and Yaniv, M. (1989). Characterization of JunD: a new member of the Jun proto-oncogene family. EMBO J. *8*, 1433–1439. https://doi.org/10.1002/j.1460-2075.1989.tb03525.x.

Houtman, J.C.D., Brown, P.H., Bowden, B., Yamaguchi, H., Appella, E., Samelson, L.E., and Schuck, P. (2007). Studying multisite binary and ternary protein interactions by global analysis of isothermal titration calorimetry data in SEDPHAT: application to adaptor protein complexes in cell signaling. Protein Sci. *16*, 30–42. https://doi.org/10.1110/ps.062558507.

Hu, Y., Ge, W., Wang, X., Sutendra, G., Zhao, K., Dedeić, Z., Slee, E.A., Baer, C., and Lu, X. (2015). Caspase cleavage of iASPP potentiates its ability to inhibit p53 and NF-κB. Oncotarget *6*, 42478–42490. https://doi.org/10.18632/oncotarget.6478.

Jiang, Y., Tsoi, L.C., Billi, A.C., Ward, N.L., Harms, P.W., Zeng, C., Maverakis, E., Kahlenberg, J.M., and Gudjonsson, J.E. (2020). Cytokinocytes: the diverse contribution of keratinocytes to immune responses in skin. JCI Insight *5*, e142067. https://doi.org/10.1172/jci.insight.142067.

Keller, S., Vargas, C., Zhao, H., Piszczek, G., Brautigam, C.A., and Schuck, P. (2012). High-precision isothermal titration calorimetry with automated peak-shape analysis. Anal. Chem. *84*, 5066–5073. https://doi.org/10.1021/ac3007522.

Kemp, C.J., Donehower, L.A., Bradley, A., and Balmain, A. (1993). Reduction of p53 gene dosage does not increase initiation or promotion but enhances malignant progression of chemically induced skin tumors. Cell *74*, 813–822. https://doi.org/10.1016/0092-8674(93)90461-x.

Khan, A., Fornes, O., Stigliani, A., Gheorghe, M., Castro-Mondragon, J.A., van der Lee, R., Bessy, A., Chèneby, J., Kulkarni, S.R., Tan, G., et al. (2018). JASPAR 2018: update of the open-access database of transcription factor binding profiles and its web framework. Nucleic Acids Res. *46*, D260–D266. https://doi.org/10.1093/nar/gkx1126.

Kolde, R. (2012). Pheatmap: Pretty Heatmaps. R Package Version, 1.0.2. https://mran.microsoft.com/snapshot/2015-05-22/web/packages/pheatmap/index.html.

Korotkevich, G., Sukhov, V., and Sergushichev, A. (2019). Fast gene set enrichment analysis. Preprint at bioRxiv. https://doi.org/10.1101/060012.

Kouwenhoven, E.N., Oti, M., Niehues, H., van Heeringen, S.J., Schalkwijk, J., Stunnenberg, H.G., van Bokhoven, H., and Zhou, H. (2015). Transcription factor p63 bookmarks and regulates dynamic enhancers during epidermal differentiation. EMBO Rep. *16*, 863–878. https://doi.org/10.15252/embr.201439941.

Li, H., and Durbin, R. (2009). Fast and accurate short read alignment with Burrows-Wheeler transform. Bioinformatics *25*, 1754–1760. https://doi.org/10.1093/bioinformatics/btp324.

Li, H., Handsaker, B., Wysoker, A., Fennell, T., Ruan, J., Homer, N., Marth, G., Abecasis, G., and Durbin, R.; 1000 Genome Project Data Processing Subgroup (2009). The sequence alignment/map format and SAMtools. Bioinformatics *25*, 2078–2079. https://doi.org/10.1093/bioinformatics/btp352.

Liao, Y., Smyth, G.K., and Shi, W. (2013). featureCounts: an efficient general purpose program for assigning sequence reads to genomic features. Bioinformatics *30*, 923–930. https://doi.org/10.1093/bioinformatics/btt656.

Liberzon, A., Birger, C., Thorvaldsdóttir, H., Ghandi, M., Mesirov, J.P., and Tamayo, P. (2015). The molecular signatures database Hallmark gene set collection. Cell Syst. *1*, 417–425. https://doi.org/10.1016/j.cels.2015.12.004.

Lichti, U., Anders, J., and Yuspa, S.H. (2008). Isolation and short-term culture of primary keratinocytes, hair follicle populations and dermal cells from newborn mice and keratinocytes from adult mice for in vitro analysis and for grafting to immunodeficient mice. Nat. Protoc. *3*, 799–810. https://doi.org/10.1038/nprot.2008.50.

Lin, B.L., Xie, D.Y., Xie, S.B., Xie, J.Q., Zhang, X.H., Zhang, Y.F., and Gao, Z.L. (2011). Down-regulation of iASPP in human hepatocellular carcinoma cells inhibits cell proliferation and tumor growth. Neoplasma *58*, 205–210. https://doi.org/10.4149/neo_2011_03_205.

Lo Muzio, L., Santarelli, A., Caltabiano, R., Rubini, C., Pieramici, T., Trevisiol, L., Carinci, F., Leonardi, R., De Lillo, A., Lanzafame, S., et al. (2005). p63 overexpression associates with poor prognosis in head and neck squamous cell carcinoma. Hum. Pathol. *36*, 187–194. https://doi.org/10.1016/j.humpath.2004.12.003.

Love, M.I., Huber, W., and Anders, S. (2014). Moderated estimation of fold change and dispersion for RNA-seq data with DESeq2. Genome Biol. *15*, 550. https://doi.org/10.1186/s13059-014-0550-8.

Lu, M., Breyssens, H., Salter, V., Zhong, S., Hu, Y., Baer, C., Ratnayaka, I., Sullivan, A., Brown, N.R., Endicott, J., et al. (2013). Restoring p53 function in human melanoma cells by inhibiting MDM2 and cyclin B1/CDK1-phosphorylated nuclear iASPP. Cancer Cell *23*, 618–633. https://doi.org/10.1016/j.ccr.2013.03.013.

Lu, M., Zak, J., Chen, S., Sanchez-Pulido, L., Severson, D.T., Endicott, J., Ponting, C.P., Schofield, C.J., and Lu, X. (2014). A code for RanGDP binding in ankyrin repeats defines a nuclear import pathway. Cell *157*, 1130–1145. https://doi.org/10.1016/j.cell.2014.05.006.

Luetteke, N.C., Phillips, H.K., Qiu, T.H., Copeland, N.G., Earp, H.S., Jenkins, N.A., and Lee, D.C. (1994). The mouse waved-2 phenotype results from a point mutation in the EGF receptor tyrosine kinase. Genes Dev. *8*, 399–413. https://doi.org/10.1101/gad.8.4.399.

Luetteke, N.C., Qiu, T.H., Peiffer, R.L., Oliver, P., Smithies, O., and Lee, D.C. (1993). TGFα deficiency results in hair follicle and eye abnormalities in targeted and waved-1 mice. Cell *73*, 263–278. https://doi.org/10.1016/0092-8674(93)90228-i.

Malnou, C.E., Salem, T., Brockly, F., Wodrich, H., Piechaczyk, M., and Jariel-Encontre, I. (2007). Heterodimerization with Jun family members regulates c-Fos nucleocytoplasmic traffic. J. Biol. Chem. *282*, 31046–31059. https://doi.org/10.1074/jbc.M702833200.

Martin, M. (2011). Cutadapt removes adapter sequences from high-throughput sequencing reads. EMBnet. J. *17*, 10. https://doi.org/10.14806/ej.17.1.200.

Mehic, D., Bakiri, L., Ghannadan, M., Wagner, E.F., and Tschachler, E. (2005). Fos and Jun proteins are specifically expressed during differentiation of human keratinocytes. J. Invest. Dermatol. *124*, 212–220. https://doi.org/10.1111/j.0022-202X.2004.23558.x.

Notari, M., Hu, Y., Koch, S., Lu, M., Ratnayaka, I., Zhong, S., Baer, C., Pagotto, A., Goldin, R., Salter, V., et al. (2011). Inhibitor of apoptosis-stimulating protein of p53 (iASPP) prevents senescence and is required for epithelial stratification. Proc. Natl. Acad. Sci. USA *108*, 16645–16650. https://doi.org/10.1073/pnas.1102292108.

Nukui, T., Ehama, R., Sakaguchi, M., Sonegawa, H., Katagiri, C., Hibino, T., and Huh, N.-H. (2008). S100A8/A9, a key mediator for positive feedback growth stimulation of normal human keratinocytes. J. Cell. Biochem. *104*, 453–464. https://doi.org/10.1002/jcb.21639.

Osada, M., Park, H.L., Nagakawa, Y., Yamashita, K., Fomenkov, A., Kim, M.S., Wu, G., Nomoto, S., Trink, B., and Sidransky, D. (2005). Differential recognition

of response elements determines target gene specificity forp53 and p63. Mol. Cell Biol. *25*, 6077–6089. https://doi.org/10.1128/mcb.25.14.6077-6089.2005.

Park, Y.-K., and Jang, B.-C. (2014). UVB-induced anti-survival and pro-apoptotic effects on HaCaT human keratinocytes via caspase- and PKC-dependent downregulation of PKB, HIAP-1, Mcl-1, XIAP and ER stress. Int. J. Mol. Med. *33*, 695–702. https://doi.org/10.3892/ijmm.2013.1595.

R Core Team (2019). R: A Language and Environment for Statistical Computing (R Foundation for Statistical Computing).

Ritchie, M.E., Phipson, B., Wu, D., Hu, Y., Law, C.W., Shi, W., and Smyth, G.K. (2015). limma powers differential expression analyses for RNA-sequencing and microarray studies. Nucleic Acids Res. *43*, e47. https://doi.org/10.1093/nar/gkv007.

Robinson, D.J., Patel, A., Purdie, K.J., Wang, J., Rizvi, H., Hufbauer, M., Ostano, P., Akgül, B., Chiorino, G., Harwood, C.A., and Bergamaschi, D. (2019). Epigenetic regulation of iASPP-p63 feedback loop in cutaneous squamous cell carcinoma. J. Invest. Dermatol. *139*, 1658–1671.e8. https://doi.org/10.1016/j.jid.2019.01.020.

Robinson, R.A., Lu, X., Jones, E.Y., and Siebold, C. (2008). Biochemical and structural studies of ASPP proteins reveal differential binding to p53, p63, and p73. Structure *16*, 259–268. https://doi.org/10.1016/j.str.2007.11.012.

Ruiz, E.J., Lan, L., Diefenbacher, M.E., Riising, E.M., Da Costa, C., Chakraborty, A., Hoeck, J.D., Spencer-Dene, B., Kelly, G., David, J.-P., et al. (2021). JunD, not c-Jun, is the AP-1 transcription factor required for Ras-induced lung cancer. JCI Insight *6*, e124985. https://doi.org/10.1172/jci.insight.124985.

Samuels-Lev, Y., O'Connor, D.J., Bergamaschi, D., Trigiante, G., Hsieh, J.-K., Zhong, S., Campargue, I., Naumovski, L., Crook, T., and Lu, X. (2001). ASPP proteins specifically stimulate the apoptotic function of p53. Mol. Cell *8*, 781–794. https://doi.org/10.1016/s1097-2765(01)00367-7.

Scheuermann, T.H., and Brautigam, C.A. (2015). High-precision, automated integration of multiple isothermal titration calorimetric thermograms: new features of NITPIC. Methods *76*, 87–98. https://doi.org/10.1016/j.ymeth.2014.11.024.

Schneider, C.A., Rasband, W.S., and Eliceiri, K.W. (2012). NIH Image to ImageJ: 25 years of image analysis. Nat Methods *9*, 671–675.

Schreiber, M., Kolbus, A., Piu, F., Szabowski, A., Möhle-Steinlein, U., Tian, J., Karin, M., Angel, P., and Wagner, E.F. (1999). Control of cell cycle progression by c-Jun is p53 dependent. Genes Dev *13 (5)*, 607–619.

Sethi, I., Romano, R.-A., Gluck, C., Smalley, K., Vojtesek, B., Buck, M.J., and Sinha, S. (2015). A global analysis of the complex landscape of isoforms and regulatory networks of p63 in human cells and tissues. BMC Genom. *16*, 584. https://doi.org/10.1186/s12864-015-1793-9.

Sethi, I., Sinha, S., and Buck, M.J. (2014). Role of chromatin and transcriptional co-regulators in mediating p63-genome interactions in keratinocytes. BMC Genom. *15*, 1042. https://doi.org/10.1186/1471-2164-15-1042.

Shao, Z., Zhang, Y., Yuan, G.-C., Orkin, S.H., and Waxman, D.J. (2012). MAnorm: a robust model for quantitative comparison of ChIP-Seq data sets. Genome Biol. *13*, R16. https://doi.org/10.1186/gb-2012-13-3-r16.

Sievers, F., Wilm, A., Dineen, D., Gibson, T.J., Karplus, K., Li, W., Lopez, R., McWilliam, H., Remmert, M., Soding, J., et al. (2011). Fast, scalable generation of high-quality protein multiple sequence alignments using Clustal Omega. Mol Syst Biol *7*, 539.

Simpson, M.A., Cook, R.W., Solanki, P., Patton, M.A., Dennis, J.A., and Crosby, A.H. (2009). A mutation inNFκB interacting protein 1causes cardiomyopathy and woolly haircoat syndrome of Poll Hereford cattle. Anim. Genet. *40*, 42–46. https://doi.org/10.1111/j.1365-2052.2008.01796.x.

Slee, E.A., Gillotin, S., Bergamaschi, D., Royer, C., Llanos, S., Ali, S., Jin, B., Trigiante, G., and Lu, X. (2004). The N-terminus of a novel isoform of human iASPP is required for its cytoplasmic localization. Oncogene *23*, 9007–9016. https://doi.org/10.1038/sj.onc.1208088.

Smirnov, A., Anemona, L., Novelli, F., Piro, C.M., Annicchiarico-Petruzzelli, M., Melino, G., and Candi, E. (2019). p63 is a promising marker in the diagnosis of unusual skin cancer. Int. J. Mol. Sci. *20*, 5781. https://doi.org/10.3390/ijms20225781.

Stephens, M. (2016). False discovery rates: a new deal. Biostatistics *18*, 275–294. https://doi.org/10.1093/biostatistics/kxw041.

Subramanian, D., Bunjobpol, W., and Sabapathy, K. (2015). Interplay between TAp73 Protein and Selected Activator Protein-1 (AP-1) Family Members Promotes AP-1 Target Gene Activation and Cellular Growth. J. Biol. Chem. *290*, 18636–18649.

Sundqvist, A., Vasilaki, E., Voytyuk, O., Bai, Y., Morikawa, M., Moustakas, A., Miyazono, K., Heldin, C.-H., ten Dijke, P., and van Dam, H. (2020). TGFβ and EGF signaling orchestrates the AP-1- and p63 transcriptional regulation of breast cancer invasiveness. Oncogene *39*, 4436–4449. https://doi.org/10.1038/s41388-020-1299-z.

Testoni, B., Borrelli, S., Tenedini, E., Alotto, D., Castagnoli, C., Piccolo, S., Tagliafico, E., Ferrari, S., Viganò, M.A., and Mantovani, R. (2006). Identification of new p63 targets in human keratinocytes. Cell Cycle *5*, 2805–2811. https://doi.org/10.4161/cc.5.23.3525.

The Gene Ontology Consortium (2019). The gene ontology resource: 20 years and still GOing strong. Nucleic Acids Res. *47*, D330–D338. https://doi.org/10.1093/nar/gky1055.

Toonen, J., Liang, L., and Sidjanin, D.J. (2012). Waved with open eyelids 2 (woe2) is a novel spontaneous mouse mutation in the protein phosphatase 1, regulatory (inhibitor) subunit 13 like (Ppp1r13l) gene. BMC Genet. *13*, 76. https://doi.org/10.1186/1471-2156-13-76.

Wang, L.-L., Xu, Z., Peng, Y., Li, L.-C., and Wu, X.-L. (2015). Downregulation of inhibitor of apoptosis-stimulating protein of p53 inhibits proliferation and promotes apoptosis of gastric cancer cells. Mol. Med. Rep. *12*, 1653–1658. https://doi.org/10.3892/mmr.2015.3587.

Wang, S., Song, R., Wang, Z., Jing, Z., Wang, S., and Ma, J. (2018). S100A8/A9 in inflammation. Front. Immunol. *9*, 1298. https://doi.org/10.3389/fimmu.2018.01298.

Weitzman, J.B., Fiette, L., Matsuo, K., and Yaniv, M. (2000). JunD protects cells from p53-dependent senescence and apoptosis. Mol. Cell *6*, 1109–1119. https://doi.org/10.1016/s1097-2765(00)00109-x.

Weston, C.R., Wong, A., Hall, J.P., Goad, M.E.P., Flavell, R.A., and Davis, R.J. (2004). The c-Jun NH2-terminal kinase is essential for epidermal growth factor expression during epidermal morphogenesis. Proc. Natl. Acad. Sci. USA *101*, 14114–14119. https://doi.org/10.1073/pnas.0406061101.

Wickham, H., Averick, M., Bryan, J., Chang, W., McGowan, L., François, R., Grolemund, G., Hayes, A., Henry, L., Hester, J., et al. (2019). Welcome to the tidyverse. J. Open Source Softw. *4*, 1686. https://doi.org/10.21105/joss.01686.

Xie, X., Lu, J., Kulbokas, E.J., Golub, T.R., Mootha, V., Lindblad-Toh, K., Lander, E.S., and Kellis, M. (2005). Systematic discovery of regulatory motifs in human promoters and 3′ UTRs by comparison of several mammals. Nature *434*, 338–345. https://doi.org/10.1038/nature03441.

Yang, J.-P., Hori, M., Sanda, T., and Okamoto, T. (1999). Identification of a novel inhibitor of nuclear factor-κb, RelA-associated inhibitor. J. Biol. Chem. *274*, 15662–15670. https://doi.org/10.1074/jbc.274.22.15662.

Zenz, R., Eferl, R., Scheinecker, C., Redlich, K., Smolen, J., Schonthaler, H.B., Kenner, L., Tschachler, E., and Wagner, E.F. (2007). Activator protein 1 (Fos/Jun) functions in inflammatory bone and skin disease. Arthritis Res. Ther. *10*, 201. https://doi.org/10.1186/ar2338.

Zhang, Y., Liu, T., Meyer, C.A., Eeckhoute, J., Johnson, D.S., Bernstein, B.E., Nusbaum, C., Myers, R.M., Brown, M., Li, W., and Liu, X.S. (2008). Model-based analysis of ChIP-seq (MACS). Genome Biol. *9*, R137. https://doi.org/10.1186/gb-2008-9-9-r137.

Zhu, L.J., Gazin, C., Lawson, N.D., Pagès, H., Lin, S.M., Lapointe, D.S., and Green, M.R. (2010). ChIPpeakAnno: a bioconductor package to annotate ChIP-seq and ChIP-chip data. BMC Bioinf. *11*, 237. https://doi.org/10.1186/1471-2105-11-237.

STAR★METHODS

KEY RESOURCES TABLE

REAGENT	SOURCE	IDENTIFIER
Antibodies		
iASPP	Ascites	LX049.3
iASPP	Ascites	LX128.5
p53	Ascites	DO-1
p53	Leica Biosystems	Cat# P53-CM5P-L, RRID: AB_2895247
p63α (H-129)	Santa Cruz	Cat# sc-8344, RRID: AB_653766
p63 (4A4)	Santa Cruz	Cat# sc-8431, RRID: AB_628091
Caspase-3 (E87)	Abcam	Cat# ab32351, RRID: AB_725946
Lamin A/C (636)	Santa Cruz	Cat# sc-7292, RRID: AB_627875
Phospho-p38 (D-8)	Santa Cruz	Cat# sc-7973, RRID: AB_670359
Phospho-JNK (G-7)	Santa Cruz	Cat# sc-6254, RRID: AB_628232
CK10	Bio Legend	Cat# 905403, RRID: AB_2749902
BrdU (BU1/75)	Abcam	Cat# ab6326, RRID: AB_305426
CD3	Abcam	Cat# ab5690, RRID: AB_305055
CD31	Abcam	Cat# ab28364, RRID: AB_726362
CD45R	BD Biosciences	Cat# 550286, RRID: AB_393581
CD8	Stratech	Cat# bs-0648R, RRID: AB_10857537
F4/80	AbD Serotec	Cat# MCA497R, RRID: AB_323279
CK1 (AF87)	Covance	Cat# PRB-149P-100, RRID: AB_291572
CK6	Covance	Cat# PRB-169P-100, RRID: AB_10063923
CK13 (EPR3671)	Abcam	Cat# ab92551, RRID: AB_2134681
CK14 (AF64)	Covance	Cat# PRB-155P, RRID: AB_292096
Loricrin (AF62)	Covance	Cat# PRB-145P-100, RRID: AB_10064155
Involucrin	Covance	Cat# PRB-140C, RRID: AB_291569
MPO (E15)	Santa Cruz	Cat# sc-34159, RRID: AB_2282186
S100a8	R&D	Cat# AF3059, RRID: AB_2184254
S100a9	R&D	Cat# AF2065, RRID: AB_2184263
HA-tag	Santa Cruz	Cat# sc-7392 HRP, RRID: AB_2894930
Mouse IgG	Ascites	N/A
JunD	Santa Cruz	Cat# sc-271938, RRID: AB_10650101
JunB	Santa Cruz	Cat# sc-8051, RRID: AB_2130023
cJun	Santa Cruz	Cat# sc-1694, RRID: AB_631263
cFos	Cell Signalling	Cat# 2250, RRID: AB_2247211
β-tubulin	Abcam	Cat# ab11308, RRID: AB_297911
β-actin (C4) HRP	Santa Cruz	Cat# sc-47778 HRP, RRID: AB_2714189
Myc-tag (4A6)	Merck Millipore	Cat# 05-724, RRID: AB_309938
Myc-Tag (9B11)	Cell Signaling	Cat# 2276, RRID: AB_331783
V5-tag (SV5-Pk1)	Bio Rad	Cat# MCA1360, RRID: AB_322378
Anti-Mouse immunoglobulins/HRP	Dako	Cat# P0161, RRID: AB_2687969
Anti-Rabbit immunoglobulins/HRP	Dako	Cat# P0217, RRID: AB_2728719
Anti-Goat immunoglobulins/HRP	Dako	Cat# P0449, RRID: AB_2617143
Anti-Mouse IgG (Fab Fragment) HRP	Sigma-Aldrich	Cat# A9917, RRID: AB_258476
AlexaFluor488 anti-Mouse IgG (H+L)	Invitrogen	Cat# A-11001, RRID: AB_2534069
AlexaFluor546 anti-Mouse IgG (H+L)	Invitrogen	Cat# A-11003, RRID: AB_141370
AlexaFluor488 anti-Rabbit IgG (H+L)	Invitrogen	Cat# A-11008, RRID: AB_143165

(*Continued on next page*)

CellPress
OPEN ACCESS

Continued		
REAGENT	SOURCE	IDENTIFIER
AlexaFluor546 anti-Rabbit IgG (H+L)	Invitrogen	Cat# A-11035, RRID: AB_143051
AlexaFluor546 anti-Rat IgG (H+L)	Invitrogen	Cat# A-11081, RRID: AB_141738
Biotinylated Anti-Mouse antibody	Vector Labs	Cat# BA-9200, RRID: AB_2336171
Biotinylated Anti-Rabbit antibody	Vector Labs	Cat# BA-1000, RRID: AB_2313606
Biotinylated Anti-Goat antibody	Vector Labs	Cat# BA-9500, RRID: AB_2336123
Bacterial and virus strains		
Escherichia coli Rosetta (DE3) Competent Cells	Merck Millipore	Cat# 70954
Escherichia coli NEB 5-alpha Competent Cells	NEB	Cat# C2987
Chemicals, peptides, and recombinant proteins		
PP1alpha peptide (WNPGGRPITPPRNSA)	TUCF	custom made
JunD peptide (WLKPAAAPPPT PLRADGAPSA)	TUCF	custom made
JunD pT71 peptide (WLKPAAAPPP[pT] PLRADGAPSA)	TUCF	custom made
NuPAGE LDS Sample Buffer (4X)	Thermo Fisher	Cat# NP0008
Benzonase Nuclease	Merck Millipore	Cat# 70746
DAPI	Sigma-Aldrich	Cat# D8417-5MG
Z-VAD-FMK	Santa Cruz	Cat# sc-3067
Z-VAD(Ome)-FMK	Santa Cruz	Cat# sc-311561A
VX-765	Sigma-Aldrich	Cat# 5313720001
JNK Inhibitor VIII	Santa Cruz Biotecnology	Cat# sc-202673
P38 MAPK Inhibitor	Sigma-Aldrich	Cat# SB 202190
MG132	Sigma-Aldrich	Cat# M7449
DMBA	Sigma-Aldrich	Cat# D3254
TPA	Sigma-Aldrich	Cat# 79346
Anisomycin	Sigma-Aldrich	Cat# A9789
Critical commercial assays		
TnT Quick Coupled Transcription/ Translation System T7	Promega	Cat# L1170
NE-PER Nuclear and Cytoplasmic Extraction Reagent	Thermo Fisher	Cat# 78835
ECL Prime Western Blotting System	Sigma-Aldrich	Cat# RPN2232
Dual luciferase Reporter assay system	Promega	Cat# E1960
Lipofectamine RNAiMAX	ThermoFisher	Cat# 13778150
Fugene 6	Promega	Cat# E2691
Deposited data		
Mouse keratinocyte RNA-seq (iASPP KO/WT)	this study	GEO: GSE188371
Mouse keratinocyte p63 ChIP-seq (iASPP KO/WT)	this study	GEO: GSE188447
Sequencing data	this study	GEO: GSE188448
Western blot and microscopy images	this study	Mendeley Data: https://doi.org/10.17632/23yddhm5sn.1
Original code	this study	Zenodo: https://doi.org/10.5281/zenodo.6887187
Original code for analysis	this study	GitHub: https://github.com/t-carroll/iASPP_keratinocyte_ChIPseq_RNAseq
Experimental models: Cell lines		
HaCaT	this study	N/A
MCF-7	this study	N/A

(*Continued on next page*)

Continued

REAGENT	SOURCE	IDENTIFIER
H1299	this study	N/A
HEKn	Promocell	Cat# C-12001
EPC-2	this study	N/A
IMOK	this study	N/A
Ker-CT	ATCC	Cat# CRL-4048, RRID: CVCL_S877
Hap1	this study	N/A
Saos-2	this study	N/A
SKML23	this study	N/A
SKML37	this study	N/A
XB2	this study	N/A
Experimental models: Organisms/strains		
Krt14-Cre^{+};*Ppp1r13l*$^{-/-}$	this study	N/A
Ppp1r13l$^{flox/flox}$;Cre^{+}ER^{T}	this study	N/A
Tg(*Krt14*-Cre)1Amc	The Jackson Lab.	RRID: IMSR_JAX:004782
Ppp1r13l$^{loxP/loxP}$	(Notari et al., 2011)	N/A
Gt(ROSA)26Sor<tm1(cre/ERT)Brn>	(Notari et al., 2011)	N/A
Oligonucleotides		
qPCR primers	this study	(see Table S1)
PCR primers for mice genotyping	this study	(see Table S1)
PCR primers for detection of mutant Ras	this study	(see Table S1)
PCR primers for cloning	this study	(see Table S1)
siGENOME Human PPP1R13L siRNA	Dharmacon	Cat# M-003815-01-0005
siGENOME Human TP63 siRNA	Dharmacon	Cat# MQ-003330-01-0002
ON-TARGETplus Human JunD siRNA	Dharmacon	Cat# LQ-003900-00-0002
RISC-free control siRNA	Dharmacon	Cat# D-001220-01-20
Recombinant DNA		
pcDNA3.1(+)-Myc-ΔNp63α	Coutandin et al. (2016)	N/A
pET-15b-His10-TEV-ASPP1 CTD (887–1090)	this study	N/A
pET-15b-His10-TEV-ASPP2 CTD (925–1128)	this study	N/A
pET-15b-His10-TEV-iASPP CTD (625–828)	this study	N/A
pET-15b-His10-TEV-iASPP CTD N813A Y814A (625–828)	this study	N/A
pCDNA3.1-iASPP-v5	Hu et al., 2015	N/A
pCDNA3.1-iASPP (1–294)-v5	Hu et al., 2015	N/A
pCDNA3.1-iASPP (295–828)-v5	Hu et al., 2015	N/A
pCDNA3.1-p53	this study	N/A
pCDNA3.1-TAp63α-HA	this study	N/A
pCDNA3.1-ΔNp63α-HA	this study	N/A
pGL3-promoter-IVL	this study	N/A
pGL3-basic-3xAP1	Addgene	Cat# 40342
pGL3-IVL-Luc	this study	N/A
pcDNA3-caspase3-WT-Myc	Addgene	Cat# 11813
pcDNA3-caspase3-C163A-Myc	Addgene	Cat# 11814
pRL-TK (Renilla)	Invitrogen	Cat# E2241
pcDNA3.1(+)-Myc-c-Fos	this study	N/A
pcDNA3.1(+)-Myc- c-Jun	this study	N/A
pcDNA3.1(+)-Myc-JunB	this study	N/A

(*Continued on next page*)

Continued

REAGENT	SOURCE	IDENTIFIER
pcDNA3.1(+)-Myc-JunD	this study	N/A
pcDNA3.1(+)-Myc-JunD ΔPxxP	this study	N/A
pcDNA3.1(+)-Myc-JunD mutPxxP	this study	N/A
pcDNA3.1(+)-Myc-JunD T71D	this study	N/A
pcDNA3.1(+)-Myc-JunD T71V	this study	N/A
pLX304-FOS-V5	Addgene	Cat# 59140
pMIEG3-c-Jun	Addgene	Cat# 40348
pCS2 Flag-JunB	Addgene	Cat# 29687
pBabe JUND-HA neo	Addgene	Cat# 58489
pENTR4-HaloTag (w876-1)	Addgene	Cat# 29644
pET-15b-His10-Halo-TEV-3xGS	this study	N/A
pET-15b-His10-Halo-TEV-3xGS_ASPP1 CTD (887–1090)	this study	N/A
pET-15b-His10-Halo-TEV-3xGS_ASPP2 CTD (925–1128)	this study	N/A
pET-15b-His10-Halo-TEV-3xGS_iASPP CTD (625–828)	this study	N/A
Software and algorithms		
GraphPad Prism 8	GraphPad Software	https://www.graphpad.com/scientific-software/prism/
Clustal Omega	Goujon et al. (2010); Sievers et al. (2011)	https://www.ebi.ac.uk/Tools/msa/clustalo/
NITPIC v1.2.7	Keller et al. (2012); Scheuermann and Brautigam (2015)	https://www.utsouthwestern.edu/labs/mbr/software/
SEDPHAT v15.2b	Houtman et al. (2007)	http://www.analyticalultracentrifugation.com/sedphat/
GUSSI v1.4.2	Brautigam (2015)	https://www.utsouthwestern.edu/labs/mbr/software/
UCSF Sparky 3.114	T. D. Goddard and D. G. Kneller, SPARKY 3, University of California, San Francisco	https://www.cgl.ucsf.edu/home/sparky/
ImageJ 1.52a	Schneider et al. (2012)	https://imagej.net/
FastQC v0.11.9	Andrews (2010)	https://www.bioinformatics.babraham.ac.uk/projects/fastqc/
Cutadapt v2.10	Martin (2011)	https://cutadapt.readthedocs.io/en/stable/
bwa v0.7.17-r1188	Li and Durbin (2009)	https://github.com/lh3/bwa
FIMO v5.0.2	Buske et al. (2010)	https://meme-suite.org/meme/doc/download.html
MAnorm v1.3.0	Shao et al. (2012)	https://manorm.readthedocs.io
SAMtools v1.10	Li et al. (2009)	http://samtools.sourceforge.net/
MACS2 v2.2.7.1	Zhang et al. (2008)	https://github.com/macs3-project/MACS/releases/tag/v2.2.7.1
ChIPpeakAnno v3.20.1	Zhu et al. (2010)	https://bioconductor.org/packages/release/bioc/html/ChIPpeakAnno.html
STAR v2.7.3a	Dobin et al. (2013)	https://github.com/alexdobin/STAR
DESeq2 v1.26.0	Love et al., 2014	https://bioconductor.org/packages/release/bioc/html/DESeq2.html
featureCounts v2.0.0	Liao et al. (2013)	http://subread.sourceforge.net/
FGSEA v1.19.2	Korotkevich et al. (2019)	https://github.com/ctlab/fgsea
limma v342.2	Ritchie et al. (2015)	https://bioconductor.org/packages/release/bioc/html/limma.html
pheatmap v1.0.12	Kolde (2012)	https://cran.r-project.org/package=pheatmap
EnhancedVolcano v1.4.0	Blighe et al. (2018)	https://bioconductor.org/packages/release/bioc/html/EnhancedVolcano.html
tidyverse v1.3.0	Wickham et al. (2019)	https://www.tidyverse.org/

(*Continued on next page*)

Continued		
REAGENT	SOURCE	IDENTIFIER
R v3.6.1	R Core Team	https://www.r-project.org/
STREME v5.4.1	Bailey and Birol, 2021	https://meme-suite.org/meme/tools/streme
Other		
Magne® HaloTag® Beads	Promega	Cat# G7282
Protein G agarose beads	Roche	Cat# 5015952001

RESOURCE AVAILABILITY

Lead contact

Further information and requests for resources and reagents should be directed to and will be fulfilled by the Lead Contact, Xin Lu (xin.lu@ludwig.ox.ac.uk).

Materials availability

All plasmids and cell lines generated in this study will be made available on request.

Data and code availability

- The sequencing data generated during this study is available at GEO under SuperSeries (GEO: GSE188448). A publicly accessible UCSC genome browser session to assist visualization of ChIP-seq results from this study is available at http://genome.ucsc.edu/s/CarrollTM/iASPP_keratinocyte_p63. Original western blots and microscopy images have been deposited to Mendeley (Mendeley Data: https://doi.org/10.17632/23yddhm5sn.1) and are publicly available as of the date of publication.
- All original code has been deposited at Zenodo and is publicly available as of the date of publication (Zenodo: https://doi.org/10.5281/zenodo.7023228). Code to reproduce this analysis can be also found at GitHub: github.com/t-carroll/iASPP_keratinocyte_ChIPseq_RNAseq.
- Any additional information required to reanalyse the data reported in this paper is available from the lead contact upon request.

EXPERIMENTAL MODEL AND SUBJECT DETAILS

Mouse experiments and genotyping

All animal procedures were approved by local ethical review and licensed by the UK Home Office (PPL: 30/2862). Animals were kept in individually ventilated cages (IVCs) at the Wellcome Trust Centre for Human Genetics, Oxford. *Ppp1r13l*$^{flox/flox}$;Cre^{+}ER^{T} mouse colonies with 4-hydroxytamoxifen (4OHT) inducible recombinase expression were generated by crossing *Ppp1r13l*$^{loxP/loxP}$ mice with Gt(ROSA)26Sor<tm1(cre/ERT)Brn> mice in a mixed genetic background of C57BL/6 and 129/Sv, as described previously (Notari et al., 2011). C57BL/6 transgenic mice containing loxP sites flanking *Ppp1r13l* exon8 were generated following 9 generations of back crossing with C57BL6 mice. They were further crossed with Tg(KRT14-cre)1Amc mice that contain transgene expressing recombinase under *Krt14* promoter (The Jackson Laboratory) in a C57BL/6 background to generate *Krt14*-Cre^{+};*Ppp1r13l*$^{-/-}$ mutant mice. The resulting mutant would have specific deletion of *Ppp1r13l* exon8 in CK14-positive basal epithelial cells only. Genotyping of *Krt14*-Cre^{+};*Ppp1r13l*$^{-/-}$ mutants and *Ppp1r13l*$^{flox/flox}$;Cre^{+}ER^{T} mouse colonies was performed using the primers listed in Table S1. For characterisation of *HRas* mutational status, a nested PCR and Restriction Fragment Length Polymorphism (RFLP) analysis of DNA samples extracted from mouse tissues was carried out. The *HRas* 61st codon was amplified using PCR1_F and PCR1_R primers on 500 ng of the extracted DNA sample to give a 267 bp fragment. Nested PCR was carried out on the PCR product with primers PCR2_F and PCR2_R to give a 176 bp product. Primers used for the analysis are listed in the Table S1. For 5-bromo-2-deoxyuridine (BrdU) incorporation, 1 mg/kg BrdU solution (Sigma-Aldrich) was injected 1 h prior to sacrificing the animals.

DMBA/TPA treatment

Dorsal hair of 8–10 week-old *Krt14*-Cre^{+};*Ppp1r13l*$^{-/-}$ C57BL/6 female mice was carefully removed with a shaver one day before the application of chemicals. A single dose of 25 μg 7,12-Dimethylbenzanthracene (DMBA) in 200 μL acetone, or acetone alone for the 12-O-tetradecanoyl-13-phorbol acetate (TPA) control group, was applied on shaved dorsal skin. This was followed by twice weekly application of 4 μg TPA in 200 μL acetone for 15 weeks. Animals were monitored closely throughout the assay in terms of weight, activity and tumour development. Mice could be observed for up to 1 year or when endpoints were reached requiring immediate termination by the Schedule 1 method as listed on the project license. For the induction of inflammation in the skin by short-term TPA treatments, 4 μg TPA in 200 μL acetone, or acetone only for negative control, was applied on days 0 and 4. Mice were sacrificed 24 h after the last TPA application and injected with BrdU 1 h prior to Schedule 1. All epidermal growths were referred to as papilloma.

Primary keratinocytes culture

Primary mouse keratinocytes were isolated as previously described (Lichti et al., 2008). Briefly, skin derived from day 2 pups was floated on 0.25% Trypsin-EDTA (Gibco) overnight at 4°C, and keratinocytes were plated on rat collagen type I (BD Bioscience) coated dishes in low-calcium medium (EMEM (Gibco), 8% calcium-stripped FBS, 50 μg/mL gentamicin, 0.05 mM calcium chloride and pen/strep mix) supplemented with 0.4 μg/mL hydrocortisone, 5 μg/mL insulin, 10 ng/mL EGF and 0.1 nM cholera toxin. Media was replaced after 24 h with or without 1 μM tamoxifen, and cells were maintained at 35°C and 7% CO_2 for 4 days. For TNFα stimulation, mouse TNFα (Peprotech) cytokine was used at a final concentration of 10 ng/mL in cell cultures after incubation in serum-free low calcium Dulbecco Modified Eagle's Medium (DMEM, Gibco) for 16 h before cytokine assays.

Cell culture and treatments

HaCaT, MCF-7, H1299 and Saos-2 cell lines were cultured in DMEM, SKML23 and SKML37 - in EMEM, primary human epidermal keratinocytes HEKn - in EpiLife (ThermoFisher), EPC2 cells - in Keratinocyte SFM medium (ThermoFisher). DMEM and EMEM media were complemented with 10% (v/v) foetal bovine serum (FBS) (Biosera), 2 mM L-glutamine (ThermoFisher), and 100 units/mL penicillin and 100 μg/mL streptomycin (ThermoFisher). Cells were grown in a humidified incubator at 37°C with 5% CO_2. Cell lines were monthly tested for mycoplasma using MycoAlert Kit (Lonza). For induction of apoptosis, cells were exposed to UV (UVP CL-1000 ultraviolet crosslinker). For inhibition of caspase activity, cells were pre-incubated with 20 μM Z-VAD-FMK, VX-765, MG132, JNK inhibitor or p38 MAPK inhibitor for 1 h before exposure to UV irradiation. For JNK activation, cells were treated with 200 ng/mL anisomycin (Sigma) for 1 h or 24 h before UV exposure. Cell lines and compounds used in this study are listed in key resources table.

Human epidermal organotypic culture

Ker-CT cells or HaCaT cells (3×10^5) were resuspended in CnT-PR keratinocyte medium (Cell-n-Tech) and seeded onto MilliCell inserts (MilliPore). Inserts were immersed in growth medium in 60 mm culture dishes. After 3 days, the medium was substituted with CnT-3D Barrier medium (Cell-n-Tech) overnight (16 h). The day after, the medium was removed, and the inserts were put in the low level CnT-3D Barrier medium to induce air-lifting. The Barrier medium was changed every two days for three weeks. Inserts with epidermal organotypic cultures were fixed in 10% formalin buffered solution for 24 h and then processed and embedded in paraffin.

Human samples

Paraffin sections of human skin samples from normal skin (n = 5), psoriasis (n = 10) and eczema (n = 10) patients were obtained from the Oxford Centre for Histopathology Research (OCHRe) under ethical approval (NRES approval: 09/H0606/78) and in collaboration with Dr. R. Asher, consultant dermatopathologist at the Oxford John Radcliffe Hospital.

METHOD DETAILS

Western blot (IB)

Cell pellets were lysed with 8 M urea buffer on ice for 30 min and lysates were then cleared by centrifugation at 13,000 g for 20 min at 4°C. For subcellular fractionation, cytoplasmic and nuclear fractions were separated using NE-PER Nuclear and Cytoplasmic Extraction kit (ThermoFisher). Bradford protein assay (BioRad) was used to measure protein concentration. Proteins were separated on SDS-PAGE and transferred onto nitrocellulose membranes, blocked at room temperature for 1 h in 5% non-fat dry milk in TBS buffer with 0.1% Tween-20 (TBST) and incubated with primary antibodies overnight at 4°C. Membranes were then washed for 30 min in TBST, incubated with horseradish peroxidase-coupled secondary antibodies (Dako) at 1:4,000 in TBST at room temperature for 1 h, and visualised with ECL Prime Western Blotting System reagents. Antibodies and reagents used in this study are listed in key resources table.

IF and confocal microscopy

Cells grown on coverslips in a 24 well plate were fixed in 4% paraformaldehyde solution for 10 min, followed by two washes in PBS and fixation in methanol for 1 min. Coverslips were washed in PBS and cells were permeabilised with 0.2% Triton X-100 solution for 10 min. Coverslips were washed in PBS and subsequently blocked with 1% fish gelatin (Sigma-Aldrich) for 1 h at room temperature followed by incubation with primary antibodies overnight at 4°C. The day after, coverslips were washed 3 times with PBS for 15 min each. Cells were then incubated with the fluorescently labelled secondary antibodies (1:1,000) and 1 μg/mL DAPI for 1 h. Samples were washed with PBS, rinsed in water and mounted using Mowiol® onto slides. Images were captured using LSM710 confocal microscope. Antibodies used in this study are listed in key resources table.

Luciferase activity assay

H1299 cells were plated on 24 well clear-bottom plates (Corning) and the day after transfected with renilla, reporter plasmids, and expression plasmids using Fugene 6 (Promega). At 24 h after transfection, the luciferase activity in cell lysates was measured using Dual luciferase Reporter assay system (Promega). Plasmids used in this study are listed in key resources table.

RNA interference

For iASPP or p63 knock-down, cells were transfected with siGENOME Human PPP1R13L, TP63 or JUND siRNAs (Dharmacon) using Lipofectamine RNAiMAX (Invitrogen) for 48 h or 72 h. siRNAs used in this study are listed in key resources table.

cDNA overexpression

Cells were plated at 70% confluency. The day after, cells were transfected using Fugene 6 transfection reagent. Cells were collected or subjected to analysis 24 h or 48 h after transfection. Plasmids used in this study are listed in key resources table.

Immunohistochemistry

Paraffin sections of mouse tissues of 4 μm thickness were deparaffinised and hydrated. For 3,3′-diaminobenzidine (DAB) staining, endogenous peroxidases were inactivated by incubating in 3% (v/v) hydrogen peroxide in methanol for 10 min at RT. Heat-induced antigen retrieval was performed in 0.01 M sodium citrate pH 6 buffer at 100°C for 4 minutes. Slides were left in the buffer to cool, and samples were blocked with 5% normal goat/donkey serum (NGS/NDS) in PBS for 1 h at room temperature. Sections were incubated in primary antibody diluted in 5% NGS/NDS overnight at 4°C inside a humidified chamber. Slides were washed with PBS, and secondary biotinylated antibodies diluted in 5% NGS/NDS were added for 1 h in the dark at RT. For DAB staining, biotinylated secondary antibodies were used, and slides were washed in PBS after incubation. Sections were then incubated in avidin-biotin peroxidase solution (VECTASTAIN Elite ABC Reagent, Vector Labs) for 15 min at room temperature. Slides were washed in PBS. Sections were then incubated in HRP substrate solution (DAB substrate kit, Vector Labs) for 10 min at room temperature. Slides were rinsed in water, counterstained in haematoxylin for 5 sec and washed again. The slides were dehydrated through an increasing gradient of ethanol, cleared in two changes of histoclear for 5 min and mounted with mounting medium (Vectamount, Vector Labs) and coverslips. For IF staining, 1 μg/mL DAPI was added along with the appropriate fluorophore conjugated secondary antibodies in the blocking solution. Slides were washed with PBS and mounted with fluoromount-G mounting media (Southern Biotech) and coverslips. For cell counting from DAB/IF stained tissue sections, averaged data for each tissue sample were collected from 3 different tissue sections at least 40 μm apart, from at least 3 different fields of view under the microscope for each section. Positive staining was quantified by automated counting using ImageJ software. Positively stained immune cells were quantitatively analysed using the publicly available Yen thresholding algorithm on ImageJ, with settings of circularity 0.25–1 and size 100–10,000 on all samples. The numbers of positive stained immune cells were then divided by the area of skin section to obtain the tumor of immune cell population within each sample. Antibodies used in this study are listed in key resources table.

Haematoxylin and eosin (H&E) staining

Paraffin sections were deparaffinised, rehydrated in a gradient of ethanol (100%, 90%, 70%, 30%) and washed in water. Slides were incubated in Harris haematoxylin for 3 minutes. Slides were rinsed in running tap water and differentiated in 1% acidic alcohol for 1 sec. Slides were washed again in tap water and immersed in Scott's water for 30 sec. Slides were rinsed in tap water and stained in eosin for 4 min. Slides were then washed in water and dehydrated with an increasing gradient of ethanol solutions. Slides were cleared using histoclear, and mounted with non-aqueous mounting medium and coverslips.

Toluidine blue staining for mast cells

Paraffin sections were deparaffinised and rehydrated as in H&E staining. Slides were incubated in 10% toluidine blue solution for 1–2 minutes and were washed in water. Slides were then dehydrated in ethanol gradients and cleared in histoclear as described in the H&E staining protocol.

mRNA extraction and qPCR

Total RNA was extracted from cells with the RNeasy Mini Kit (Qiagen) following the manufacturer's protocol. On-column DNase I digestion was performed. For cDNA conversion, 500 ng of total RNA sample were retrotranscribed using the SuperScript II First Strand Synthesis System (Invitrogen) with oligo(dT) primers. Quantitative PCR (qPCR) was performed on cDNA samples using the QuantiTect SYBr Green PCR Kit (Qiagen) on the 7500 real time PCR system (Applied Biosystems). Each qPCR reaction was carried out in duplicates. The expression level of target genes was analysed using the comparative C_t method ($\Delta\Delta C_t$) with *GAPDH* as the internal control. The experiment was repeated on three independent cultures of primary mouse keratinocytes. Primers used for the analysis are listed in the Table S1.

Library preparation and sequencing

For RNA sequencing, wild-type and iASPP deficient keratinocytes, prepared as described above, were subjected to RNA extraction protocol using RNAeasy Mini Kit (Qiagen) using DNase I digestion step. One μg of isolated RNA was used for RNA-sequencing, as previously described (Kouwenhoven et al., 2015). In brief, rRNA was depleted using the Ribo-Zero rRNA Removal Kit (Epicentre) according to the manufacturer's instructions, followed by RNA fragmentation. The fragmented rRNA depleted RNA was used for the first strand and second strand reactions. Subsequently, five ng of ds-cDNA was prepared for sequencing. Adaptors were ligated to the DNA fragments, followed by a pre-PCR of 4 cycles, size selection (~300 bp) and subsequently 11 cycles of PCR amplification. Single-read sequencing (1 × 42nt) was then performed on final libraries using the Illumina NextSeq 500. For ChIP sequencing,

primary mouse keratinocytes from WT and iASPP KO mice were subjected to ChIP using the H129 anti-p63 antibody (Santa Cruz), followed by standard Illumina library preparation, as described previously (Kouwenhoven et al., 2015). In brief, a total of 6 ng ChIPped DNA or input control DNA as measured by Qubit (Invitrogen) was prepared for sequencing. Adaptors were ligated to the DNA fragments, followed by a pre-PCR of 4 cycles, size selection (~300 bp) and subsequently 11 cycles of PCR amplification. Final libraries were then sequenced using a paired-end format (2 × 43nt) on the Illumina NextSeq, following standard manufacturer's protocols. Approximately 50 million reads were acquired for ChIP samples, and 30 million reads for input controls.

Cloning and mutagenesis

iASPP (92–828) and iASPP (295–828) were obtained by PCR from pcDNA3.1(+)-iASPP and subsequently subcloned into the pcDNA3.1 vector by TA cloning. pcDNA3.1(+)-Myc-c-Fos, pcDNA3.1(+)-c-Jun, pcDNA3.1(+)-Myc-JunB and pcDNA3.1(+)-Myc-ΔNp63α were generated by subcloning of respective PCR products in a pcDNA3.1(+) vector with an N-terminal Myc-tag using BamHI and XhoI restriction sites. pcDNA3.1(+)-Myc-JunD was generated similarly but instead using BamHI and XbaI restriction sites. For recombinant expression in *E. coli*, respective PCR products were introduced in pET-15b-His10-TEV (N-terminal His_{10}-tag followed by TEV protease cleavage site) or pET-15b-His10-Halo-TEV-3xGS (N-terminal His_{10}-tag followed by Halo-tag, TEV protease cleavage site and GSGSGS linker) by subcloning using BamHI and XhoI restriction sites. The pET-15b-His10-Halo-TEV-3xGS vector was generated by subcloning Halo in pET-15b vector using NcoI and XhoI restriction sites. The His_{10}-tag was added by the 5′ oligo and the TEV protease cleavage site, linker, BamHI site and stop codon by the 3′ oligo. All successive constructs carrying mutations were produced by site-directed mutagenesis. Primers used for cloning are listed in Table S1.

IP

Cell pellets were lysed in lysis buffer from NE-PER Nuclear and Cytoplasmic Extraction kit (ThermoFisher) following manufacturer's instructions. Then, protein G agarose beads were blocked with 5% FBS for 1 h at 4°C. After blocking, the beads and the antibody were added to the lysates and incubated overnight at 4°C under rotation. The day after, the beads were washed 4 times with NP40 lysis buffer and eluted in 4x Laemmli buffer at 95°C for 10 min. The eluates were separated by SDS-PAGE.

Protein expression and purification

All expression plasmids were transformed in *E. coli* strain Rosetta DE3 (Novagen) for protein production. Cells were grown in 2xYT medium to an OD of ~0.8. Expression was carried out for ~18 h at 16°C. Cells were harvested, resuspended in ice-cold IMAC A buffer (25 mM Tris pH 7.8, 200 mM NaCl, 20 mM ß-ME, 5% Glycerol and 25 mM imidazole) supplemented with lysozyme (Sigma), RNAse (Sigma), DNAse (Sigma) and protease inhibitors (Carl Roth) and lysed by sonification. Lysate was cleared by centrifugation at 4°C. All subsequent purification steps were performed at 4°C using an ÄKTA Purifier chromatography system (Cytiva). All proteins were purified by Ni^{2+}-affinity chromatography in the first step using HisTrap columns (Cytiva). Halo fusion proteins were then subjected to ion-exchange (IEX) chromatography. Before loading them on HiTrap Q columns (Cytiva), salt concentration was reduced to under 100 mM by dilution with IEX A buffer (25 mM Tris pH7.8, 50 mM NaCl; 20 mM ß-ME, 5% Glycerol). Protein bound to the IEX column was eluted with a gradient from 50 mM to 1,000 mM NaCl over 20 column volumes using IEX B buffer (25 mM Tris pH7.8, 1,000 mM NaCl, 20 mM ß-ME, 5% Glycerol). As a final purification step, Halo fusion proteins were loaded on a Superdex 200 size exclusion chromatography (SEC) column (Cytiva) equilibrated in the final storage buffer (25 mM HEPES pH 7.5, 150 mM NaCl; 0.5 mM TCEP; 10% Glycerol). His-tagged proteins were instead supplemented with His-tagged TEV protease for cleavage of the His_{10}-Tag after Ni^{2+}-affinity purification and dialysed over night at 4°C in IMAC A buffer. The cleaved His_{10}-Tag and TEV protease were remove by reverse Ni^{2+}-affinity purification. The flow-through containing the cleaved protein was then further purified by IEX chromatography, as for the Halo fusion proteins. In a final polishing step, proteins were loaded on a Superdex 75 SEC column (Cytiva) equilibrated in the final storage and assay buffer (25 mM HEPES pH 7.5, 150 mM NaCl; 0.5 mM TCEP). All proteins were concentrated by centrifugation, snap frozen in liquid nitrogen and stored at −80°C until use. Plasmids used in this study are listed in key resources table.

Halo pulldown

Myc-tagged AP1 family proteins and p63 were produced by *in vitro* translation (Promega) of pcDNA3.1(+) plasmids for 90 min at 30°C. After, reactions were supplemented with Benzonase (Merck Millipore) and incubated for 30 min at 30°C to digest DNA and RNA, followed by centrifugation for 10 min at 13,000 rpm to remove any aggregates. 100 μg of purified Halo fusion proteins were immobilized on 25 μL magnetic Halo beads slurry in pulldown buffer (50 mM HEPES pH7.5, 200 mM NaCl, 0.5 mM TCEP, 0.1% Surfactant P-20) for at least 4 h at 4°C. After removal of unbound protein, loaded beads were incubated with 20 μL *in vitro* translated protein in pulldown buffer for 3 h at 4°C under rotation. For displacement pulldown assays, JunD or PP1 peptides were added to a final concentration of 25 μM. After incubation, beads were washed four times with 400 μL ice-cold pulldown buffer and bound proteins were eluted by incubation in 1x LDS sample buffer supplement with DTT for 10 min at 70°C. Five μL of *in vitro* translated protein was mixed with 95 μL SDS-PAGE sample buffer serving as input sample. The samples were analysed by western blot using anti-Myc tag antibody. The signal intensities were quantified by densitometric analysis using ImageJ software. Relative pulldown efficiency was calculated by normalizing the pulldown signals to the corresponding input signal. Statistical significance was calculated by RM 1-way ANOVA.

Isothermal calorimetry

All isothermal calorimetry (ITC) experiments were performed on VP-ITC (MicroCal). Dissolved peptides as well as purified proteins were dialysed in ITC buffer (25 mM HEPES pH 7.5, 150 mM NaCl, 500 μM TCEP) overnight at 4°C. All measurements were performed with 25 titration steps of 10 μL each. 450 μM JunD or JunD pT71 peptide were titrated to 30 μM ASPP family CTDs at 12°C. Binding curves were analysed using NITPIC (Keller et al., 2012; Scheuermann and Brautigam, 2015) including subtraction of dilution heat measurement of each ligand. The thermodynamic parameters (ΔH and TΔS), the equilibrium dissociation constant (K_D) and the incompetent fractions of A (incfA) and B (incfB) were determined with SEDPHAT (Houtman et al., 2007) assuming an AB hetero association model. Final ITC figures were generated by GUSSI (Brautigam, 2015).

Bioinformatic analyses

For both RNA-seq and ChIP-seq data, raw FASTQ files were assessed for quality and adapter content using FastQC (Andrews, 2010). Cutadapt (Martin, 2011) was used to remove adapters prior to alignment to the *Mus musculus* reference genome (mm10).

RNA-seq data were aligned using STAR v2.7.3a (Dobin et al., 2013) with the GENCODE vM25 primary assembly comprehensive gene annotation set (Frankish et al., 2019). Gene counts were then generated using featureCounts (Liao et al., 2013). Differential expression was conducted in R (R Core Team, 2019) using DESeq2 (Love et al., 2014). Briefly, DESeq2 was used to identify differentially expressed genes after iASPP knockout, with adjustment for batch-specific effects. Genes having an adjusted $p < 0.05$ were determined to be significant. Moderated $\log_2$ fold changes generated with the 'ashr' method (Stephens, 2016) were used for volcano plots and to rank genes for gene set enrichment analysis (GSEA) using FGSEA (Korotkevich et al., 2019). Gene set collections (Hallmark (Liberzon et al., 2015), Gene Ontology Biologic Process (The Gene Ontology Consortium, 2019), and TFT Legacy (Xie et al., 2005) curated by MSigDB were employed with FGSEA. For heatmaps, VST-normalized counts with batch-specific effects removed by limma (Ritchie et al., 2015) were used as input to the pheatmap package (Kolde, 2012). Further downstream visualization was done using EnhancedVolcano (Blighe et al., 2018) and tidyverse (Wickham et al., 2019).

ChIP-seq reads were aligned to mm10 using BWA-backtrack (Li and Durbin, 2009). Reads aligning to the major autosome and sex chromosome contigs were selected, and duplicates were removed using SAMtools (Li et al., 2009). MACS2 (Zhang et al., 2008) was used to call peaks with a significance threshold of $q < 0.05$, and to generate bigwig files normalized to signal per million reads for visualization. Peaks overlapping with ENCODE Blacklist (Amemiya et al., 2019) regions were discarded for downstream analysis. MAnorm (Shao et al., 2012) was then used to calculate the average amplitude (A) values for each peak across iASPP KO and WT conditions, as well as the normalized log fold-change (M) between the two conditions and the associated p-value of differential enrichment for each peak. A p-value threshold of $5 \cdot 10^{-3}$ was selected to define significantly enriched peaks in either iASPP WT or iASPP KO. Sequences of enriched peak regions were extracted using bedtools getfasta. These sequences were used to generate the average p63 motif in both iASPP WT- and iASPP KO-enriched peaks using STREME (Bailey and Birol, 2021), with default arguments, except for increasing the maximum motif length to 25 and searching for a maximum of one motif.

For correlation of M values with the presence of TF motifs in each peak, FIMO (Buske et al., 2010) was used to find the top hits for each of the 579 TF motifs in the JASPAR 2018 CORE vertebrates non-redundant motif set (Khan et al., 2018). Briefly, options for FIMO were set to output all possible matches for each combination of motifs and peak sequences, and this output was filtered to select only the most significant match for each motif and peak sequence. Within results for each motif, adjustment of FIMO p-values across all peaks was performed using the Benjamini-Hochberg false discovery rate (FDR) procedure (Benjamini and Hochberg, 1995), and a FDR of $1 \cdot 10^{-3}$ was used as a threshold to call the presence or absence of a motif in each peak region. We then calculated the Spearman correlation coefficient between M values and the FIMO score of the top-ranked hit for each of the 579 JASPAR motifs across all peaks, and ranked all motifs by this correlation coefficient.

A UCSC genome browser session was created for the visualization of the ChIP-seq data alongside the results from MACS2, MAnorm, and FIMO (for selected motifs). This genome browser session is publicly available at http://genome.ucsc.edu/s/CarrollTM/iASPP_keratinocyte_p63.

Peaks were then annotated to the transcription start site (TSS) of the nearest feature in the mm10 genome using ChIPpeakAnno (Zhu et al., 2010). For the purposes of this study, we considered all peaks within 2 kb upstream or downstream of the TSS to bind in the promoter region, while all peaks outside the promoter region within 20 kb upstream or downstream were considered to bind in the enhancer region. For purposes of integration with the RNA-seq data, only peaks annotated to genes passing DESeq2's low count expression filter were considered. To give evidence as to whether membership of a gene in various subcategories of interest from this integrated dataset may play a role in its differential expression, we compared the proportions of differentially upregulated and downregulated genes in the subcategory to these proportions calculated from the full integrated dataset. A Pearson's chi-squared test was performed between the two proportions, and the resulting p-value and odds ratio were used to evaluate the significance and magnitude of the effect that membership in these subcategories has on the probability of differential expression.

QUANTIFICATION AND STATISTICAL ANALYSIS

All statistical analyses were performed in Prism 8 (GraphPad), with the exception of RNA-seq and ChIP-seq analyses, which were conducted in R. The logrank (Mantel-Cox) test was performed to determine the statistical significance of tumor-free survival of *Krt14*-iASPP mice in the DMBA/TPA cohort. T-testing was used in data on papilloma number per mouse and to compare epidermal

features and cell composition between two groups (iASPP KO and WT). In case of multiple t-testing, p-values were corrected using the 2-stage Benjamini, Krieger, & Yekutieli (BKY) FDR procedure. For quantification of immunohistochemical staining, qPCR analyses and pulldown experiments, one-way or two-way/mixed ANOVA tests were used to determine significance of differences between multiple groups. Dunnett's multiple-comparison tests (against control column only) were conducted to obtain p values for specific comparisons following one-way ANOVAs, and Šidák's multiple comparison test was used following two-way/mixed-effect ANOVAs. Adjusted p values <0.05 were considered significant. Specific p-values for related figures are listed below: Figures 7C - $P = 2 \times 10^{-6}$ by logrank test test (WT vs KO); Figure 1D - *P* by multiple t-test (between WT and KO for each time point) with false discovery (BKY) correction for multiple testing; Figure 3C - Displayed adjusted *P* for expression were calculated by DESeq2; Figure 4A - *P* were calculated for differences between iASPP genotype at each timepoint by RM two-way ANOVA followed by Šidák's correction for multiple comparison testing; Figure 4C - *P* by t-test with false discovery (BKY) correction for multiple testing; Figure 4D - *P* by t-test with false discovery (BKY) correction for multiple testing; Figure 5A - *P* by RM 1-way ANOVA followed by Dunnett's multiple comparison test; Figure 6A - *P* by RM 1-way ANOVA was performed separately for EV, p53, TAp63, and ΔNp63 groups. *P* for differences between iASPP expression vectors and control were then calculated within each group using Dunnett's multiple comparison test; Figure 6B - *P* by RM 1-way ANOVA (for each of cJun, JunB, and JunD groups). *P* was then calculated for differences in pulldown efficiency between ASPP family members and control using Dunnett's post-hoc test for multiple testing; Figure 6H - *P* by mixed effects 2-way ANOVA, followed by calculation of *P* for differences in luciferase activity with or without iASPP-V5 for each JunD variant using Šidák's correction for multiple comparison testing. Significance tests and thresholds used for RNA-seq and ChIP-seq analyses are stated in the related methods sections.

Cell Reports, Volume *41*

Supplemental information

Mutant Ras and inflammation-driven skin tumorigenesis is suppressed via a JNK-iASPP-AP1 axis

Khatoun Al Moussawi, Kathryn Chung, Thomas M. Carroll, Christian Osterburg, Artem Smirnov, Rebecca Lotz, Paul Miller, Zinaida Dedeić, Shan Zhong, Martin Oti, Evelyn N. Kouwenhoven, Ruth Asher, Robert Goldin, Michael Tellier, Shona Murphy, Huiqing Zhou, Volker Dötsch, and Xin Lu

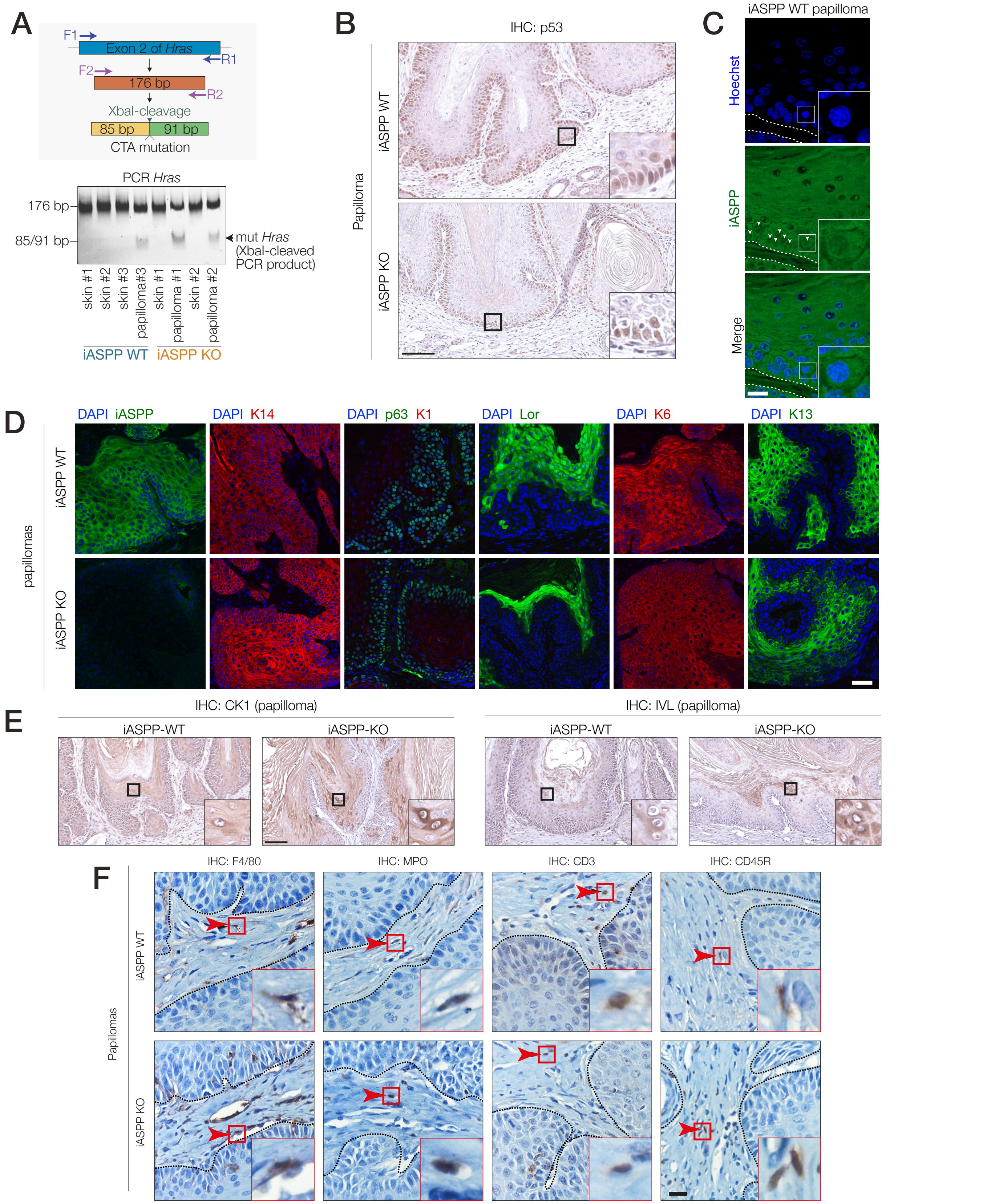

Figure S1. iASPP suppresses skin tumorigenesis driven by chemically-induced mutant Ras and inflammation. Related to Figure 1 **(A)** (Top) Scheme showing the location of primers for detection of Hras mutation. (Bottom) PCR detection of Hras mutation in skin and papilloma samples from WT and iASPP KO mice. **(B)** IHC analysis of p53 expression in WT and iASPP-KO papilloma samples. Scale bar 100 μm. **(C)** IF staining of iASPP expression in a WT papilloma sample. Scale bar 25 μm. **(D)** Immunofluorescence staining of iASPP, cytokeratin 14, p63, cytokeratin 1, loricrin, cytokeratin 6, and cytokeratin 13 in papillomas from WT and KO mice. Scale bar 25 μm. **(E)** IHC analysis of keratin 1 (CK1) and involucrin (IVL) expression in WT and iASPP-KO papilloma samples. Scale bar 100 μ m. **(F)** Immunohistochemical analysis of expression of F4/80 (macrophages), MPO (neutrophils), CD3 (T cells), and CD45R (B cells) in papillomas from WT and KO mice. Arrowheads indicate examples of stained immune cells. Dotted line shows boundary between epidermis and stroma. Scale bar 25 μm.

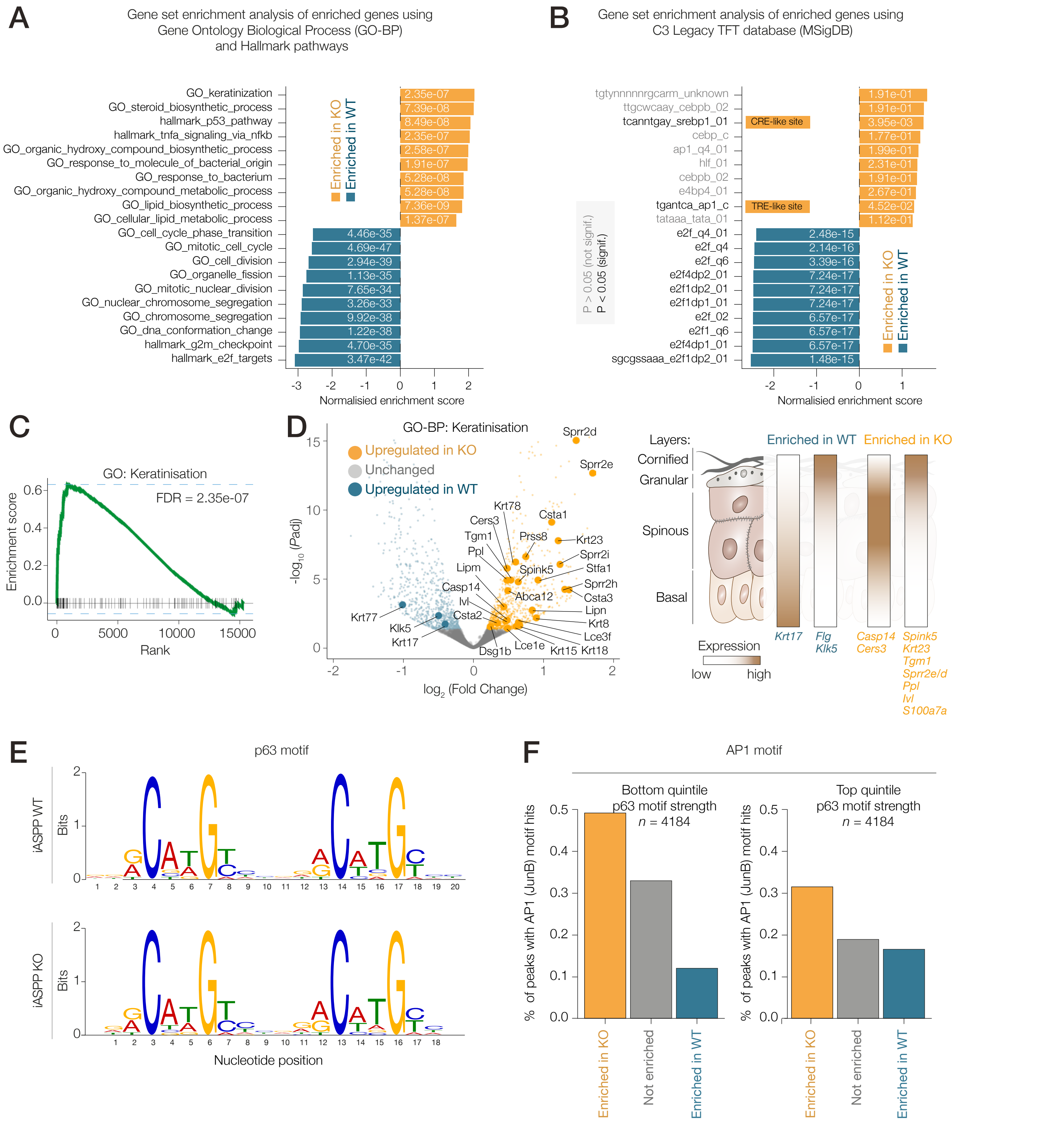

Figure S2. Loss of iASPP selectively enhances p63 binding to regions that often contain AP1 motifs. Related to Figure 2 **(A)** FGSEA pathway analysis using moderated fold changes to identify significantly enriched pathways within the Hallmark and Gene Ontology Biological Process pathway collections. The top 10 significant pathways in iASPP KO and WT were identified by the adjusted p-value, with the magnitude of the normalized enrichment score (NES) as tiebreaker if needed. For each pathway, the NES is represented by the length of the bar along the x-axis, and the adjusted p-value is displayed on top of the bar. **(B)** FGSEA pathway analysis as in A but using the C3 Legacy TFT database (MSigDB), which lists genes that have highly conserved instances of TF motifs in their promoter regions. Only two pathways reach significant enrichment in iASPP KO following p-value adjustment; these are in bold for emphasis. **(C)** FGSEA enrichment plot for GO-BP Keratinization pathway. **(D)** (Left) Genes from the GO Keratinization pathway with an adjusted p-value < 0.05 in differential expression analysis are highlighted on the RNA-seq plot. (Right) Schematic showing expression levels of modulated genes from GO keratinization pathway across the layers of human epidermis. **(E)** Consensus p63 motifs identified in iASPP WT-enriched and KO-enriched p63 peak regions (with MAnorm p < 5e-3), as determined by STREME analysis. **(F)** Peak sequences were scored for the presence or absence of the AP1 (JUNB) motif using an FDR threshold of 1e-3 with the representative MA0490.1/JUNB motif. The percentages of peaks containing this AP1 motif were calculated for the peak enrichment categories defined in (A), and the results are plotted in these barplots. The figure is faceted depending on the quintiles of the score assigned by FIMO to the top hit for a p63 motif in each sequence; on the left facet, the bottom quintile (weakest/absent p63 motifs) subset is shown, while the right facet shows the top quintile of peaks, with the highest similarity to the canonical p63 motif in JASPAR.

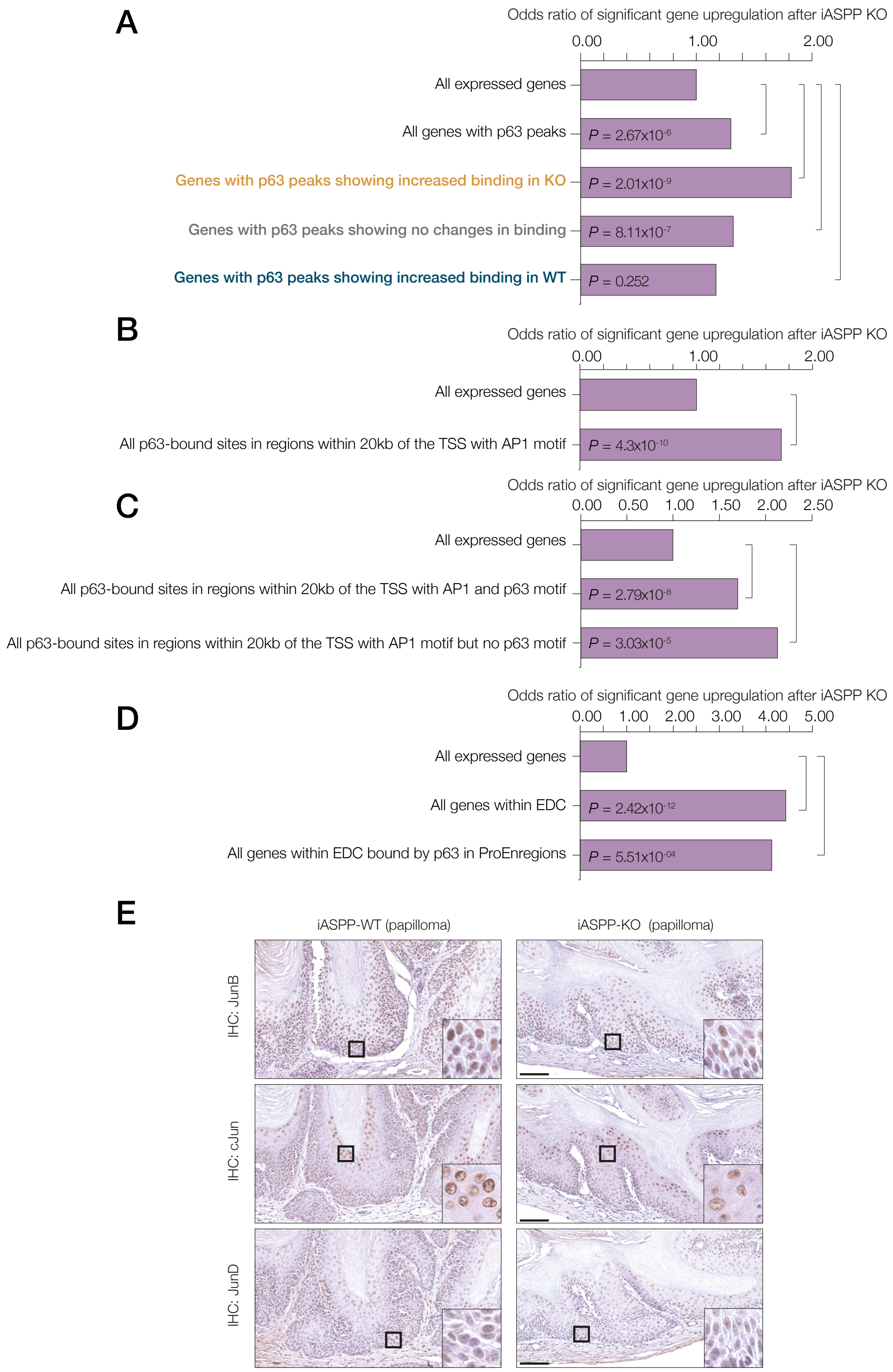

Figure S3. iASPP depletion affects expression of epidermal differentiation genes. Related to Figure 3 **(A)** Barplots show the odds of significant gene upregulation after iASPP KO based on membership in categories defined from ChIP-seq data. In each case, we investigate the defined subsets in relation to the full reference set of 15,320 expressed genes, of which 969 (6.3%) are upregulated. For each bar, we provide the odds ratio, defined as the proportion of upregulated genes in the subset divided by the same proportion in the reference set, and the associated value as calculated by a Pearson's chi-squared test. In the top subplot, subcategories are defined by the ChIP-seq enrichment class defined in 2B; in the bottom, the subcategories are defined by the presence or absence of AP1 or p63 motifs, with a FIMO FDR of 0.001 used as a threshold to call the presence of motifs in each peak region sequence. **(B)** Barplots as in A defined by the presence of AP1 motif. **(C)** Barplots as in A defined by the presence of AP1 motif and presence or absence of p63 motifs. **(D)** Odds ratios for significant gene upregulation and the associated p-value are shown for comparisons between all expressed genes in the EDC (n=68, 27.9% significantly upregulated) and the reference set of all expressed genes (n=15,320, 6.3% significantly upregulated). A comparison is also done with a subset of expressed EDC genes with a p63 binding site within 20kb of the TSS (n=23, 26.1% significantly upregulated). **(E)** IHC analysis of JunB, cJun, and JunD expression in WT and iASPP-KO papilloma samples. Scale bar 100 μm.

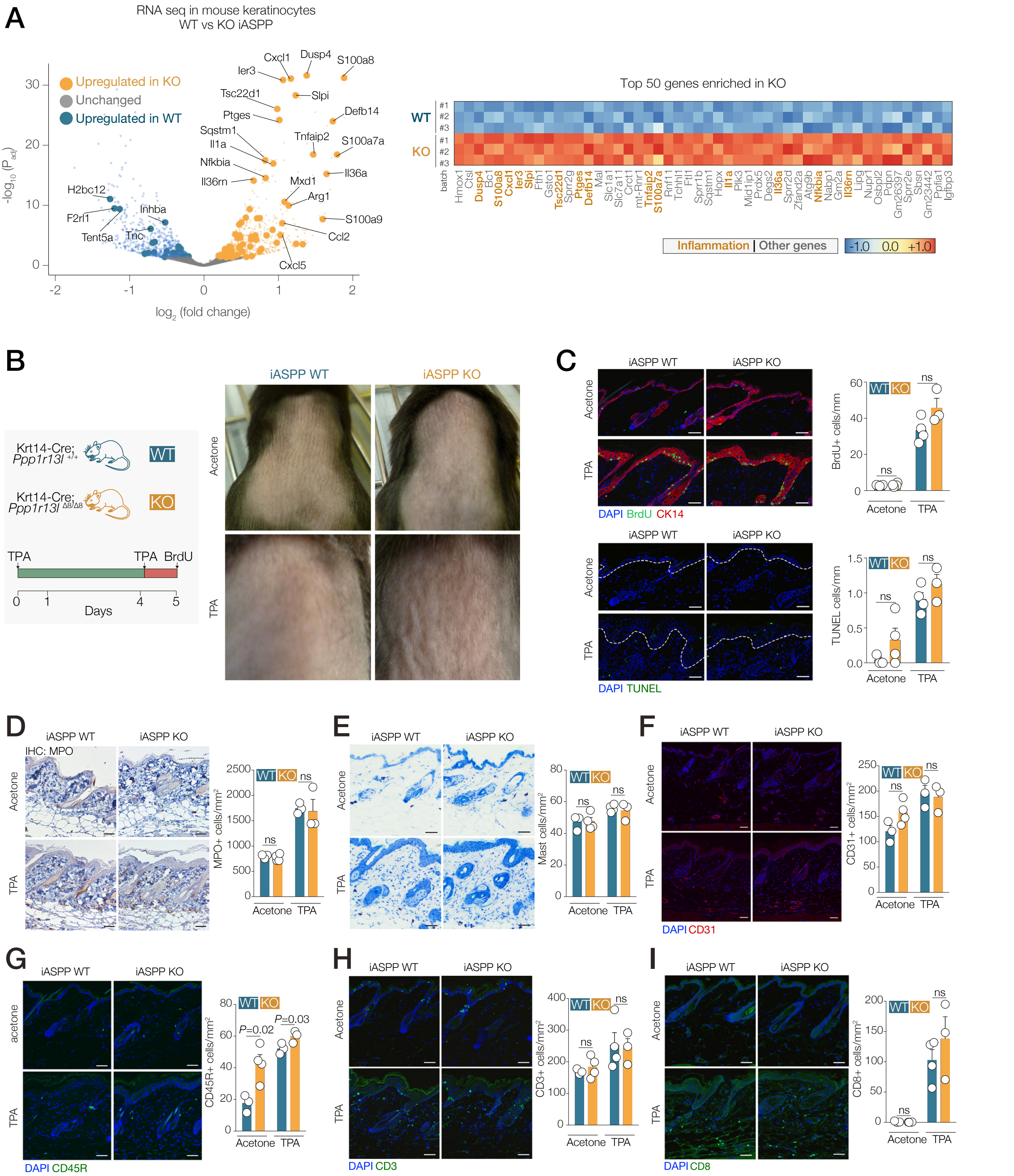

Figure S4. iASPP deficient keratinocytes induce pro-inflammatory gene expression in vitro and attract macrophages in vivo. Related to Figure 4 **(A)** Volcano plot shows the moderated log fold changes and associated adjusted p-value after iASPP knockout. Genes are assigned a colour depending on whether they are determined to be as significant (adjusted p-value < 0.05) and in which condition they are significantly enriched in. Significantly differentially expressed genes in two inflammatory pathways (Hallmark: TNFA_SIGNALING_VIA_NFKB and GO-BP: RESPONSE_TO_BACTERIUM) are labelled. On the right, heatmap showing the 50 genes most significantly enriched in iASPP KO. Genes belonging to these same two pathways are highlighted. **(B)** (Left) Schematic illustration of the TPA inflammation induction protocol. (Right) Representative images of treated skin in WT and iASPP KO mice. **(C)** Immunofluorescence analysis (left) and quantification (right) of BrdU-positive and TUNEL-positive cells in skin samples from WT and iASPP KO mice upon acetone or TPA treatment. Dashed line shows boundary between epidermis and dermis. n=3 (WT, acetone cohort), n=4 (KO, acetone cohort), n=4 (WT, TPA cohort), n=3 (KO, TPA cohort). **(D)** Immunohistochemical analysis (left) and quantification (right) of MPO-positive cells in skin samples from WT and iASPP KO mice upon acetone or TPA treatment. **(E)** Immunohistochemical analysis (left) and quantification (right) of mast cells in skin samples from WT and KO mice upon TPA treatment. **(F)** Immunofluorescence analysis (left) and quantification (right) of CD31-positive cells in skin samples from WT and KO mice upon TPA treatment. **(G)** Immunofluorescence analysis (left) and quantification (right) of CD45R-positive cells in skin samples from WT and KO mice upon TPA treatment. **(H)** Immunofluorescence analysis (left) and quantification (right) of CD3-positive cells in skin samples from WT and KO mice upon TPA treatment. **(I)** Immunofluorescence analysis (left) and quantification (right) of CD8-positive cells in skin samples from WT and KO mice upon TPA treatment. For C-I, *P* value was calculated by t-test with false discovery (BKY) correction for multiple testing. Scale bar 100 µm.

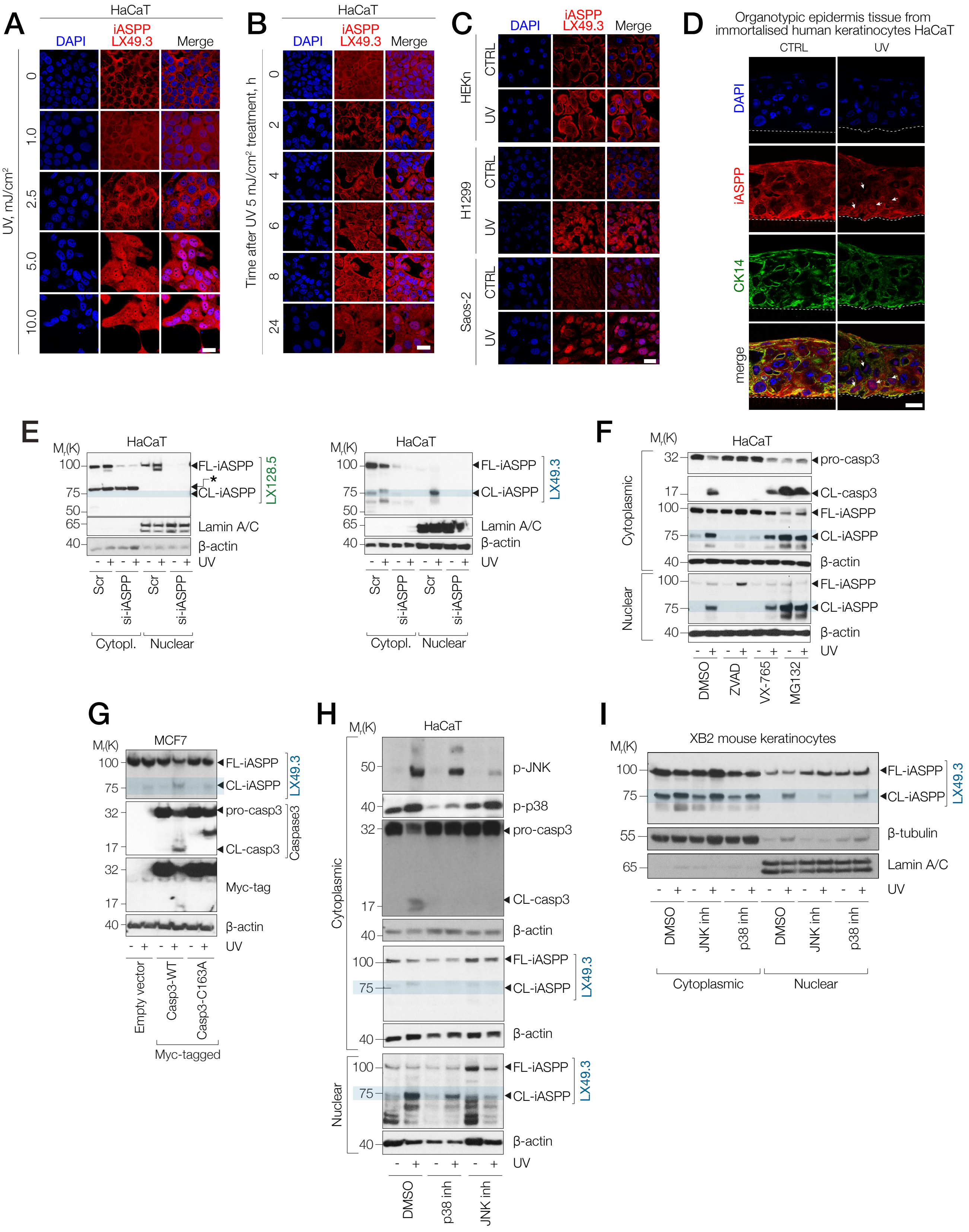

Figure S5. Cross regulation between iASPP and JNK/AP1. Related to Figure 5 **(A)** Immunofluorescence analysis of iASPP colocalisation in HaCaT cells irradiated with increasing doses of UV. Cells were subjected to immunostaining 24h after UV irradiation. Scale bar 10 µm. **(B)** Immunofluorescence analysis of iASPP localisation in HaCaT cells irradiated with 5 mJ/cm^2 UV. Cells were subjected to immunostaining at 2h, 4h, 6h, 8h, and 24h after UV irradiation. Scale bar 10 µm. **(C)** Immunofluorescence analysis of iASPP localisation in HEKn, H1299, and Saos-2 cells irradiated with 5 mJ/cm^2 UV. Cells were subjected to immunostaining 24h after UV irradiation. Scale bar 10 µm. **(D)** Immunofluorescence analysis of iASPP (LX49.3) and cytokeratin 14 (CK14) expression levels in organotypic epidermis tissue from HaCaT cells. Tissues were irradiated with 150 mJ/cm^2 UV. Sections were collected and subjected to immunofluorescence analysis 24h after UV irradiation. Scale bar 25 µm. **(E)** Western blot analysis of iASPP expression levels in cytoplasmic and nuclear fractions of HaCaT cells upon 5 mJ/cm^2 UV irradiation after iASPP knock-down. Lamin A/C was used as a marker of nuclear fraction. iASPP was detected by either LX49.3 or LX128.5 anti-iASPP antibodies. * refers to a non-specific band recognized by LX128.5 at around 78 kDa in the cytoplasmic fraction in the left panel of S5E. **(F)** Western blot analysis of iASPP and caspase-3 expression in cytoplasmic and nuclear fractions of HaCaT cells pre-treated with either pan-caspase inhibitor (ZVAD), caspase-1/4 inhibitor (VX-765) or proteasome inhibitor (MG132) for 1h and exposed to 5 mJ/cm^2 UV irradiation. Cells were collected 24h after UV irradiation. **(G)** Western blot analysis of iASPP, caspase-3, and myc-tag expression levels in MCF7 cells transfected with either wild-type (WT) or mutant (C163A) myc-tagged caspase-3 for 24h and then exposed to 5 mJ/cm^2 UV irradiation. Cells were collected 24h after UV irradiation. **(H)** Western blot analysis of iASPP, p-JNK, p-38, and caspase 3 expression levels in the samples from Figure 5H after subcellular fractionation. **(I)** Western blot analysis of iASPP expression levels in the same treatments after subcellular fractionation of XB2 mouse keratinocytes. β-tubulin and lamin A/C as fractionation controls.

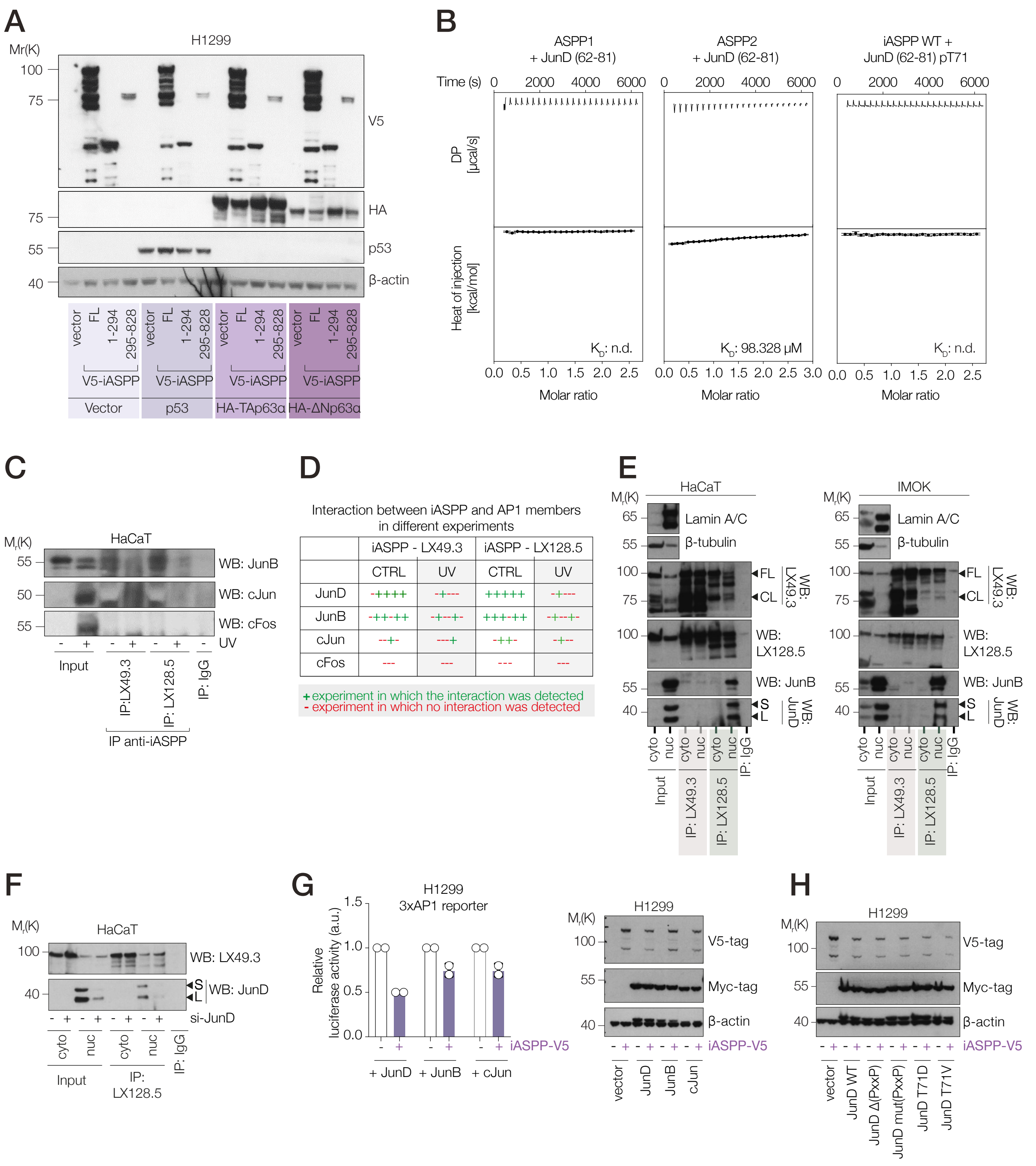

Figure S6. iASPP SH3 domain interacts with JunD N-terminal PxxP motif to inhibit AP1 transcriptional activity. Related to Figure 6 **(A)** Western blot analysis of expression of p63, p53 and iASPP mutants in samples from Figure 6A. **(B)** ITC results of JunD peptide binding to either ASPP1 (left) or ASPP2 CTD (middle) and JunD pT71 peptide binding to iASPP CTD (right). Raw titration profiles are displayed in the top diagrams and integrated heat in the bottom diagram. Best fit of single-site binding model is shown as a solid black line with the resulting equilibrium binding constant (KD). All other fit parameters are provided in Table S2. **(C)** Co-immunoprecipitation using anti-iASPP antibodies in lysates from Figure 6F. Western blot for JunB, c-Jun, and c-Fos. **(D)** Table showing whether AP1 members were co-immunoprecipitated with iASPP using LX49.3 or LX128.5 anti-iASPP antibodies in different experiments (biological replicates). "+" (green) indicates that interaction was detected, while "-" (red) indicates that no interaction was detected. **(E)** Co-immunoprecipitation using anti-iASPP antibodies in lysates of HaCaT or IMOK cells subjected to subcellular fractionation. Western blot for JunB and JunD. β-tubulin and lamin A/C as fractionation controls. **(F)** Co-immunoprecipitation using anti-iASPP antibody LX128.5 in lysates of HaCaT cells subjected to subcellular fractionation after JunD knock-down (si-JunD). Western blot for JunD and iASPP. **(G)** (Left) Luciferase activity assay in H1299 cells using 3xAP1 reporter after overexpression of V5-tagged iASPP combined with Myc-tagged JunD, JunB or c-Jun. Luciferase luminescence was normalised over renilla luminescence. Values shown are mean fold change over empty vector ± SD, n = 2 (biological replicates). (Right) Western blot confirming the overexpression of V5-tagged and Myc-tagged proteins. **(H)** Western blot confirming the overexpression of V5-tagged and Myc-tagged proteins in the samples from the Figure 6H.

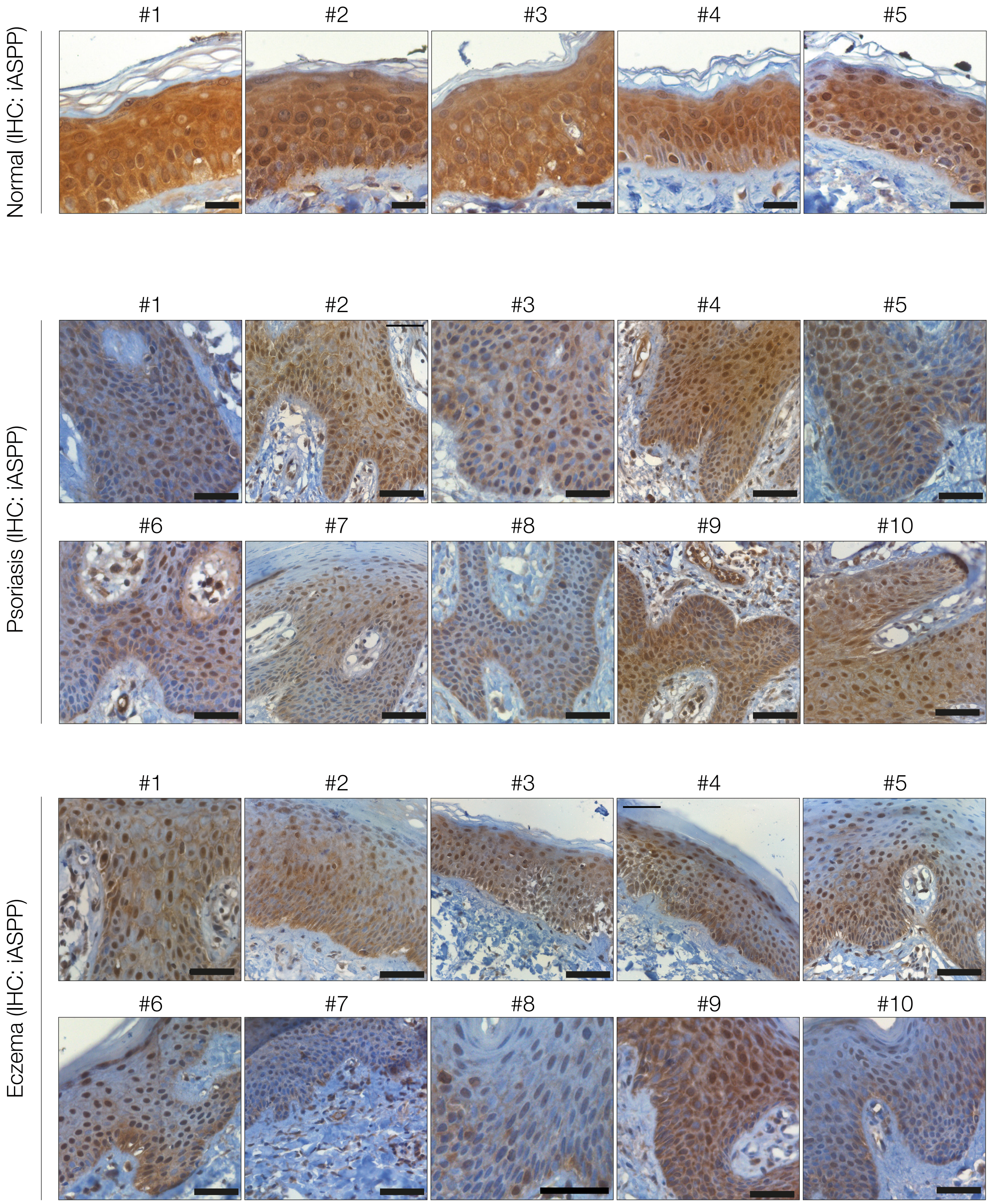

Figure S7. iASPP expression in human skin samples. Related to Figure 5. IHC analysis of iASPP expression on samples of normal human skin or skin from patients affected by psoriasis or eczema. Scale bar 100 μm.

Table S1. List of the primers used in the study, Related to "*Mouse Experiments and Genotyping*", "*mRNA extraction and qPCR*" and "*Cloning and mutagenesis*" sections of the STAR Methods

**qPCR primers**	
Name	Sequence
Ppp1r13l – For	AGACTCCAGCACCTCAACAGA
Ppp1r13l – Rev	GACCGTGCCTTCATCTGC
S100a8 – For	CCTTTGTCAGCTCCGTCTTC
S100a8 – Rev	TAGAGGGCATGGTGATTTCC
S100a9 – For	GCTGCATGAGAACAACCCAC
S100a9 – Rev	TCCCTTTAGACTTGGTTGGGC
IL1a – For	ATCAGCAACGTCAAGCAACG
IL1a – Rev	AGGTGCTGATCTGGGTTGGA
Il36a – For	CCCACTCATTCTGACCCAAG
Il36a – Rev	GAGAGAGTGCCACAGAGCAA
Il36b – For	TGCCTGCTGTCATAACTTCG
Il36b – Rev	CAAGATTGTGGAGGGAAAGC
IL6 – For	AGAAGGAGTGGCTAAGGACCA
IL6 – Rev	AACGCACTAGGTTTGCCGA
IL1b – For	CATCTCGGAGCCTGTAGTGC
IL1b – Rev	CGTGGACCTTCCAGGATGAG
TNF – For	GGCAGGTCTACTTTGGAGTCATTGC
TNF – Rev	ACATTCGAGGCTCCAGTGAATTCGG
CXCL1 – For	CCAGAGCTTGAAGGTGTTGC
CXCL1 – Rev	TCTCCGTTACTTGGGGACAC
GAPDH – For	CAGCAAGGACACTGAGCAAGA
GAPDH – Rev	GGCCCCTCCTGTTATTATGG

**PCR primers for mice genotyping**	
Name	Sequence
iASPP exon8 – FLP2	CCGAATTGGAGAAGTGAAGC
iASPP exon8 – I8-2	CCGAATTGGAGAAGTGAAGC
iASPP exon8 – E8-2	AGAGCAGCCTCAGAGCATGG
loxP – FLP2	CCGAATTGGAGAAGTGAAGC
loxP – FRANT9	GGGTAGGAAAAAGGGCTGAG
K14-Cre – R	ATTCTCCCACCGTCAGTACG
K14-Cre – 1	GCTCTCTGTCACCCTGGCTA
Actin – For	GGTGTCATGGTAGGTATGGGT
Actin – Rev	CGCACAATCTCACGTTCAG
Cre+ERT – F	CATTTGGGCCAGCTAAACAT
Cre+ERT – B	ATTCTCCCACCGTCAGTACG

Table S1. List of the primers used in the study (continued)

**PCR primers for detection of mutant Ras**	
Name	Sequence
Ras-Mut-1 – For	CTGTGAATTCTCTGGTCTGAGGAG
Ras-Mut-1 – Rev	TAGGTGGCTCACCTGTACTG
Ras-Mut-2 – For	CTAAGCCTGTTGTTTTGCAGGAC
Ras-Mut-2 – Rev	GGAACTTGGTGTTGTTGATGGC
**PCR primers for cloning**	
Name	Sequence
ΔNp63 AA2 BamHI – For	TACTTCCAGGGATCCTTGTACCTGGAAAACAATGCCCAG
p63α Stop XhoI – Rev	TACTTCCAGCTCGAGTCACTCCCCCTCCTCTTTGATGC
c-Fos AA3 BamHI – For	TACTTCCAGGGATCCTTCTCGGGCTTCAACGCAGAC
c-Fos Stop XhoI – Rev	TACTTCCAGCTCGAGTCACAGGGCCAGCAGCGTGG
c-Jun AA2 BamHI – For	TACTTCCAGGGATCCACTGCAAAGATGGAAACGACCTTC
c-Jun Stop XhoI – Rev	TACTTCCAGCTCGAGTCAAAATGTTTGCAACTGCTGCGTTAGC
JunB AA2 BamHI – For	TACTTCCAGGGATCCTGCACTAAAATGGAACAGCCCTTC
JunB Stop XhoI – Rev	TACTTCCAGCTCGAGTCAGAAGGCGTGTCCCTTGACC
JunD AA2 BamHI – For	TACTTCCAGGGATCCGAAACACCCTTCTACGGCGATG
JunD Stop XbaI – Rev	TACTTCCAGTCTAGATCAGTACGCGGGCACCTGGTG
JunD ΔPxxP – For	CTGCGGCCGCGCCGGCCGACGGCGCCCC
JunD ΔPxxP – Rev	GGGGCGCCGTCGGCCGGCGCGGCCGCAG
JunD mutPxxP – For	CTGCGGCCGCGCCGGCCCCTACCGCCCTGGCCGCCGACGGCGCCCC
JunD mutPxxP – Rev	GGGGCGCCGTCGGCGGCCAGGGCGGTAGGGGCCGGCGCGGCCGCAG
JunD T71D – For	GGCCGCGCCGCCTCCTGACCCCCTGCGCGCCGACG
JunD T71D – Rev	CGTCGGCGCGCAGGGGGTCAGGAGGCGGCGCGGCC
JunD T71V – For	GGCCGCGCCGCCTCCTGTGCCCCTGCGCGCCGACG
JunD T71V – Rev	CGTCGGCGCGCAGGGGCACAGGAGGCGGCGCGGCC
HisX Halo NcoI – For	TACTTCCAGCCATGGGCCATCATCATCATCATCACCATCACCATCACGGC AGCGCAGAAATCGGTACTGGCTTTC
Halo TEV 3xGS BamHI Stop XhoI – Rev	TACTTCCAGCTCGAGTTAGGATCCGGAACCAGAGCCGGAACCCTGGAAGT ACAGGTTTTCGCTGCCAGTGGTTGGCTCGCCGGAAATCTCCAGCGTCGACAG CCAGCGCG
ASPP1 CTD BamHI – For	TACTTCCAGGGATCCAACCCCCCTGGCACTGCTC
ASPP1 CTD Stop XhoI – Rev	TACTTCCAGCTCGAGTTAGGCGAGTGTTCGCTGTCG
ASPP2 CTD BamHI – For	TACTTCCAGGGATCCAACCCCCCTTGCTTTACTGCTAG
ASPP2 CTD Stop XhoI – Rev	TACTTCCAGCTCGAGTTAGGCCAAGCTCCTTTGTCTTG
iASPP CTD BamHI – For	TACTTCCAGGGATCCCTGAACCCGCTGGTTCTG
iASPP CTD Stop XhoI – Rev	TACTTCCAGCTCGAGTTACTAAACTTTAGAACGCTGCGG
iASPP N813A Y814A – For	GGCTACGTGCCGCGGGCCGCCTTCGGGCTGTTCCCCAGG
iASPP N813A Y814A – Rev	CCTGGGGAACAGCCCGAAGGCGGCCCGCGGCACGTAGCC

Table S2. ITC fit parameters. Related to Figure 6

Component A (cell)	Component B (syringe)	K_D (95% CI) [μM]	ΔH (95% CI) [kcal/mol]	IncfA (95% CI)	IncfB (95% CI)
iASPP CTD	JunD peptide	4.575 (4.353 to 4.808)	-9.277 (-9.443 to -9.120)	0.079 (0.068 to 0.090)	0
ASPP1 CTD	JunD peptide	n.d.	n.d.	n.d.	n.d.
ASPP2 CTD	JunD peptide	98.328 (87.078 to 111.517)	-4.334 (-4.674 to -4.041)	0	0
iASPP CTD N813A Y814A	JunD peptide	n.d.	n.d.	n.d.	n.d.
iASPP CTD	JunD pT71 peptide	n.d.	n.d.	n.d.	n.d.
